# Selective Prebiotic Synthesis of α‐Threofuranosyl Cytidine by Photochemical Anomerization

**DOI:** 10.1002/anie.202101376

**Published:** 2021-03-26

**Authors:** Ben W. F. Colville, Matthew W. Powner

**Affiliations:** ^1^ Department of Chemistry UCL 20 Gordon Street London WC1H 0AJ UK

**Keywords:** nucleotides, origin of life, photochemistry, prebiotic chemistry, TNA

## Abstract

The structure of life's first genetic polymer is a question of intense ongoing debate. The “RNA world theory” suggests RNA was life's first nucleic acid. However, ribonucleotides are complex chemical structures, and simpler nucleic acids, such as threose nucleic acid (TNA), can carry genetic information. In principle, nucleic acids like TNA could have played a vital role in the origins of life. The advent of any genetic polymer in life requires synthesis of its monomers. Here we demonstrate a high‐yielding, stereo‐, regio‐ and furanosyl‐selective prebiotic synthesis of threo‐cytidine **3**, an essential component of TNA. Our synthesis uses key intermediates and reactions previously exploited in the prebiotic synthesis of the canonical pyrimidine ribonucleoside cytidine **1**. Furthermore, we demonstrate that erythro‐specific 2′,3′‐cyclic phosphate synthesis provides a mechanism to photochemically select TNA cytidine. These results suggest that TNA may have coexisted with RNA during the emergence of life.

## Graphical abstract

TNA cytidine, a candidate precursor to RNA cytidine at the origin of life, is synthesized from prebiotic precursors via a high‐yielding, regio‐ and stereo‐selective, protecting‐group free synthesis utilizing an efficient photochemical anomerization. Phosphorylation also enables selective photodestruction of the contaminating *erythro*‐diastereomer enriching any potential pool of TNA monomers.

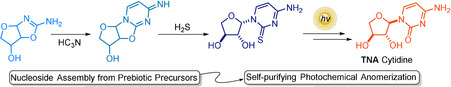

Nucleic acids are essential to modern biology, and several theories for the origin of life rely on the chemical (non‐enzymatic) emergence of nucleic acids.[^1^, ^2^] The “RNA world theory”,^[3]^ for example, which postulates that RNA emerged as life's first genetic polymer, is supported by the universal dual genetic and phenotypic functions of RNA in biology.[^4^, ^5^] It has been suggested that selective chemical synthesis of RNA's monomers may have foreshadowed its selective inclusion into life. However, alternative scenarios have been proposed,^[6]^ and it remains unclear whether RNA was the first genetic polymer to emerge during the evolution of life,^[7]^ therefore alternatives must be evaluated. The structure and composition of a nucleic acid's backbone is a key property that dictates its ability to form a Watson–Crick duplex, and is therefore crucial to heritable information transfer. Threose nucleic acid (TNA), built from a 4‐carbon tetrose sugar, was identified as a potential progenitor to RNA (Figure 1).^[8]^ TNA can form stable duplexes with not only itself but also with RNA and DNA.[^8^, ^9^, ^10^] Although TNA's backbone connectivity results from a truncated 3′‐2′‐phosphodiester linkage (whereas RNA and DNA are linked by 5′‐3′‐phosphodiesters), TNA's *trans*‐vicinal phosphodiesters can stretch into a conformation that can accommodate Watson–Crick base pairing (Figure 1). Homo‐duplexes of TNA are more hydrolytically stable than RNA, instead comparable to DNA.[^11^, ^12^, ^13^, ^14^] Mixed pools of TNA and RNA monomers could form chimeric oligomers, which have been shown to promote the formation of homogeneous oligomers during template‐directed ligation.^[15]^ TNAs can also fold into tertiary structures that bind targets with high affinity and specificity.^[16]^ These properties of TNA have enabled the in vitro evolution of TNAs, which can be synthesized by DNA polymerases,[^10^, ^17^, ^18^, ^19^] RNA‐dependent RNA polymerases,^[20]^ and even TNA polymerases.^[21]^

**Figure 1:**
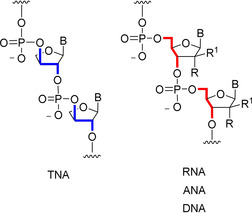
Nucleic acid backbones of TNA, RNA (R=OH, R^1^=H), ANA (R=H, R^1^=OH), and DNA (R=H, R^1^=H). Truncated 3′‐2′‐phosphodiester of TNA (blue) compared to natural 5′‐3′‐phosphodiesters (red). In TNA the α‐anomer of l‐threose nucleic acid is observed to Watson–Crick base pair with RNA/DNA. B=nucleobase.

Despite TNA's remarkable pairing properties and recent progress toward the chemical synthesis of RNA monomers,[^2^, ^22^, ^23^, ^24^, ^25^, ^26^, ^27^, ^28^, ^29^, ^30^] the prebiotic synthesis of TNA's monomers has yet to be thoroughly evaluated. TNA is a simpler structure than RNA, with one fewer chiral carbon atom in its sugar moiety, and conceptually, TNA's sugar moiety can be assembled from two achiral C_2_ building blocks (whereas RNA's sugar moiety requires one C_2_ and one chiral C_3_ building block).^[8]^ TNA nucleosides (adenosine, inosine, uridine) have been observed in glycosylation reactions,[^25^, ^26^] but these glycosylation reactions require preformed nucleobases and invoke formose‐type reactions to deliver separately formed tetrose sugars. We, and others, have recently elucidated prebiotically plausible syntheses of RNA, ANA, and DNA nucleosides which bypass these challenging glycosylation reactions,[^22^, ^27^, ^28^, ^29^, ^30^] and now we extend this work to evaluate this mode of prebiotic synthesis for TNA. A key element of these (RNA, ANA, DNA) syntheses has been common aminooxazoline intermediates, and it is of note that tetrose aminooxazolines (i.e., threose aminooxazoline, **TAO** and erythrose aminooxazoline, **EAO**) can also be efficiently synthesized by the same reactions that yield pentose aminooxazolines (e.g., ribose aminooxazoline, **RAO**) (Figure 2).^[31]^ Moreover, we have previously demonstrated that equimolar reaction of **2AO** with C_2_ and C_3_ sugars (glycolaldehyde **5**, glyceraldehyde, and dihydroxyacetone) significantly favours synthesis of **TAO** over **RAO** (7:1, see ref. [27] and Supplementary Figure 19 & 20 therein). Therefore, we envisaged a prebiotic synthesis that yields **TAO** would in turn provide access to TNA.[^22^, ^32^]

**Figure 2:**
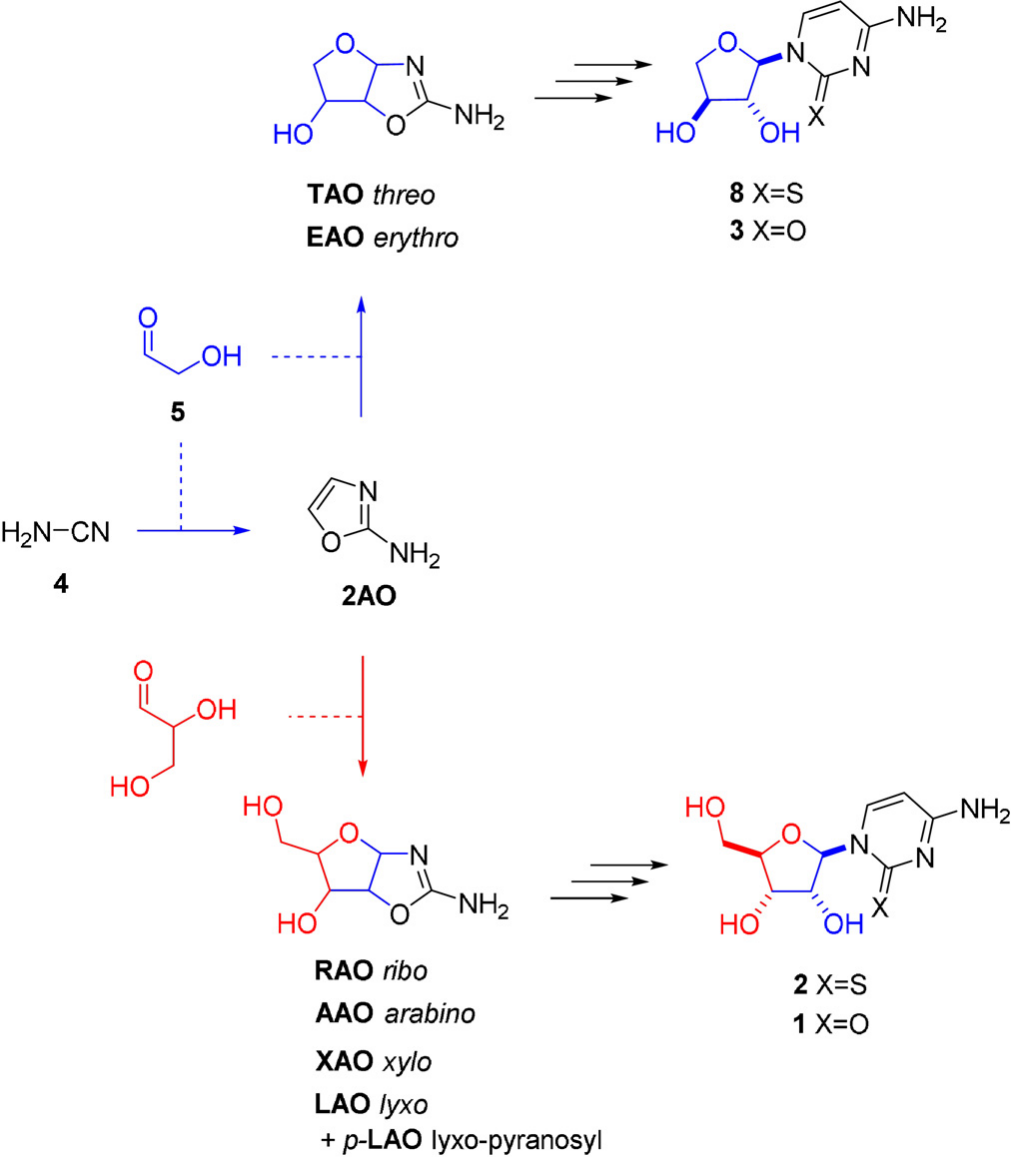
Divergent synthesis of RNA and TNA cytidines through the reaction of cyanamide with prebiotically plausible aldehydes. The five‐carbon RNA backbone requires sequential reaction of glycolaldehyde **5** (blue) and glyceraldehyde (red) generating four furanosyl pentose aminooxazolines (**RAO**, **AAO**, **XAO**, and **LAO**) and one pyranosyl pentose aminooxazolines (p‐**LAO**). The four‐carbon TNA backbone requires only glycolaldehyde **5** (blue) and only generates two furanosyl isomers (**EAO** and **TAO**).

Irradiation with UV‐light provides a powerful mechanism to bring about molecular change in prebiotic chemistry,[^2^, ^22^, ^24^, ^28^, ^30^, ^33^, ^34^] and UV‐light would have been an important energy source on the prebiotic Earth.^[35]^ The prebiotic Earth's atmosphere is predicted to have been significantly more transparent to UV‐light, due to lower O_2_/O_3_ concentration than the modern atmosphere, leading to more UV‐light (*λ*>204 nm) irradiating the surface of Earth.[^36^, ^37^, ^38^, ^39^] Pioneering work by Orgel and co‐workers demonstrated that irradiation of the unnatural α‐anomer of cytidine gave a 4 % yield of the natural β‐anomer **1**.^[40]^ While low yielding, this demonstrated the plausibility of photochemical anomerization for prebiotic cytidine syntheses. Further studies have revealed that the competing cyclization to oxazolidinones, via attack of the C2′‐hydroxy on the C2‐position of the base, was problematic for pyrimidine ribonucleoside anomerization.^[33]^ Sutherland and co‐workers have recently demonstrated that irradiation (254 nm) of α‐*ribo*‐thiocytidine in neutral water with hydrogen sulfide gave a much higher photoanomerization yield of thiocytidine **2** than previously observed for anomerizations of cytidines,^[24]^ even without blocking the 2′‐hydroxy moiety to prevent oxazolidinone formation.[^41^, ^42^] Proposed TNA precursor aminooxazolines (e.g., **TAO**) have been shown to form alongside those aminooxazolines that can yield RNA,^[31]^ but the transformation of **TAO** to TNA has not been reported. Accordingly, we set out to investigate the protecting‐group‐free, prebiotic synthesis of *threo*‐cytidine **3** by UV‐light driven photoanomerization (Figure 2).

Previous work has established that cyanamide **4** and glycolaldehyde **5** react efficiently to form 2‐aminooxazole **2AO** (>80 %)^[22]^ and that tetrose aminooxazolines, **TAO** and **EAO**, can be formed in high yields (>90 %) from the reaction of **2AO** with glycolaldehyde **5** (Figure 2).[^27^, ^31^, ^32^] Here, we also observed the reaction of **TAO** and **EAO**, with cyanoacetylene (HC_3_N),[^43^, ^44^] to give near‐quantitative conversion to their respective anhydronucleosides, **6** and **7** (Figure 3 and Supplementary Figure 1). We next turned our attention to the synthesis of tetrose nucleoside **3**. We envisaged H_2_S addition to tetrose anhydronucleosides **6** and **7** would regioselectively position a sulfur atom at C2 on the nucleobase and enable photochemical anomerization to yield the TNA monomer **3** (via thiocytidine **8**).

**Figure 3:**
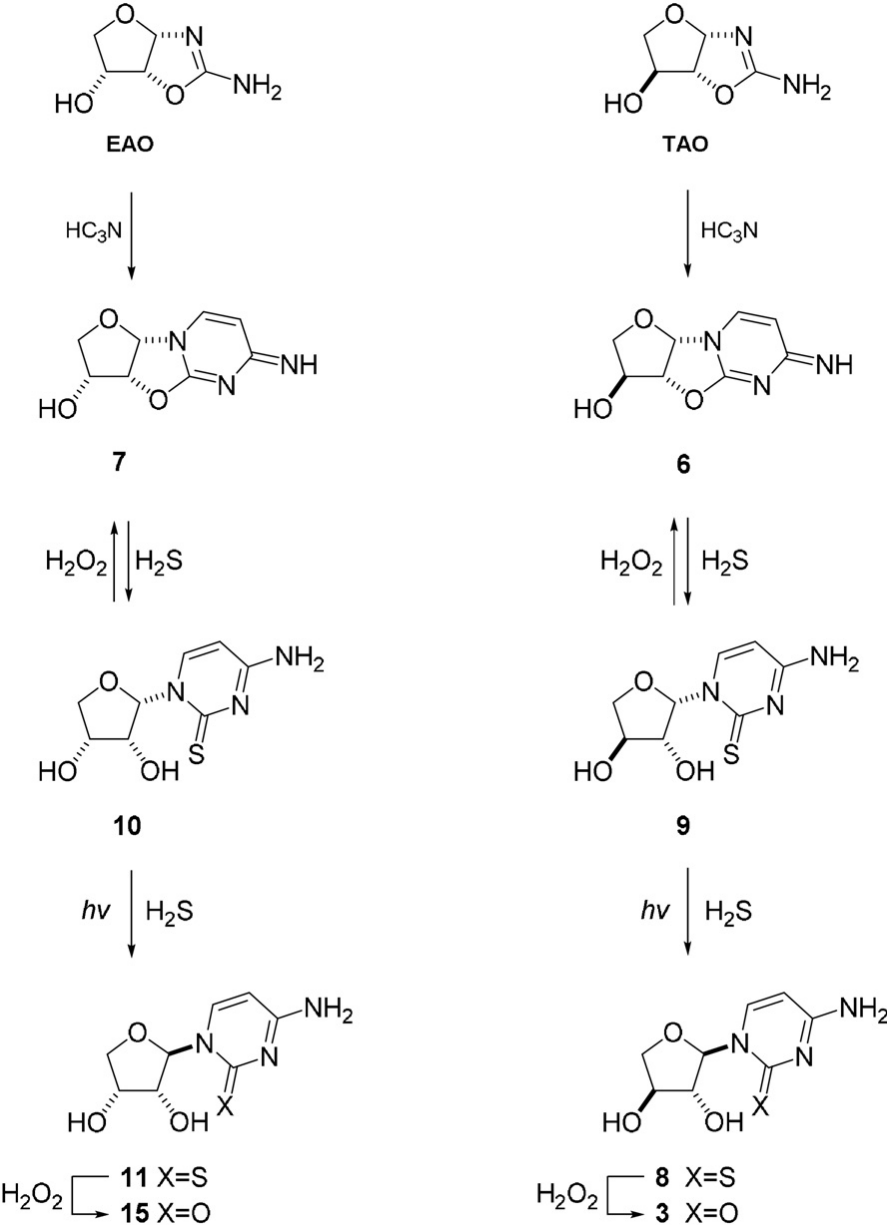
Synthesis of tetrose nucleosides by photochemical anomerization. Tetrose aminooxazolines (**EAO** and **TAO**) react with cyanoacetylene (HC_3_N) to yield tetrose 2,2′‐anhydrocytidines **6** and **7**. Reversible thiolysis of **6** and **7**, followed by selective photoanomerization of the 1′,2′‐*cis*‐nucleosides **9** and **10** yields 1′,2′‐*trans*‐nucleosides **8** and **11**. Oxidative hydrolysis of *trans*‐1′,2′‐thiocytidine **8** and **11** furnishes cytidines **3** and **15**, whereas oxidation of *cis*‐1′,2′‐thiocytidines **9** and **10** furnishes anhydrocytidines **6** and **7**.

As anticipated, we observed smooth thiolysis of **6** and **7** to their corresponding β‐thiocytidines **9** and **10** in formamide (78 %, Figure 3 and Supplementary Figure 2). Therefore, we sought to investigate the stereochemical inversion of the anomeric position for the tetrose series to understand if this would enable delivery of TNA monomers. We then envisaged the C2‐sulfur would be removed by a facile oxidation,^[28]^ or by hydrolysis,^[24]^ to yield the canonical nucleobase.

After 1 day of irradiation with UV‐light (254 nm) in water at pH 7 with H_2_S (26 mm) we observed conversion of β‐*threo*‐thiocytidine **9** (25 mm) to α‐*threo*‐thiocytidine **8** (46 %). After 1.5 days irradiation α‐*threo*‐thiocytidine **8** (51 %) was observed to be the major product (Supplementary Figure 6). Spiking with an authentic standard of **8** (See Supplementary Information for synthesis of authentic **8**) confirmed the effective photochemical anomerization.

Concomitant photoanomerization of *threo*‐ and *erythro*‐ thiocytidine **9** and **10** was observed to occur at a similar rate (Figure 4), to furnish **8** (40 %) and **11** (42 %) after 1 day. Maximum conversion was observed after 2 days (**8** 52 %, **11** 51 %). Conversely, when **8** and **11** were irradiated (254 nm) in neutral water for 3 days with hydrogen sulfide we observed significant formation of 2‐thiocytosine **12** from both α‐isomers, but interestingly little reversion to the β‐anomers (Supplementary Figure 14).

**Figure 4:**
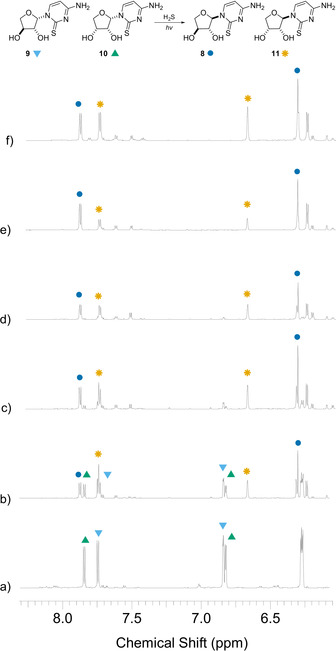
^1^H NMR (700 MHz, noesygppr1d, H_2_O/D_2_O (9:1), 6.1–8.3 ppm) spectra showing the irradiation (254 nm) of **9** (18 mm) and **10** (16 mm) at pH 7 with H_2_S (113 mm): a) before the irradiation; and after b) 1 day; c) 2 days; d) 3 days irradiation. Spectrum e) following addition of authentic **8**. Spectrum f) following addition of authentic **11**. (C6)−H and (C1′)−H resonances are labelled: **9** (▾), **10** (▴), **8** (•), and **11** (✶).

As anticipated the sulfur atom at C2 can be facilely removed, to generate the canonical nucleobase. For example, reaction with hydrogen peroxide^[28]^ rapidly converted **8** to the TNA nucleoside, α‐*threo*‐cytidine **3**, in quantitative yield (Supplementary Figure 4). Interestingly, however, *cis*‐1′,2′‐isomers **9** and **10** were observed to rapidly cyclize to reform their corresponding anhydronucleoside **6** and **7** (Figure 3 & Supplementary Figure 3) upon exposure to hydrogen peroxide, rather than undergo conversion to their respective cytidine hydrolysis products.

Encouraged by the anomerization of tetrose thiocytidines, we next considered the role and timing of phosphorylation in the sequence towards TNA. Phosphorylation of prebiotic nucleosides has been demonstrated in the dry state with urea^[45]^ and we sought to utilize this simple phosphorylation in our work. In the *ribo*‐series, photoanomerization and conversion of thiocytidine **2** to cytidine **1** must occur prior to phosphorylation.^[24]^ These constraints are imposed by the formation of 2′,3′‐cyclic phosphates which, when in a *trans*‐configuration with respect to the 2‐thiocytidine nucleobase, undergo (destructive) cyclization to 2,2′‐thioanhydronucleotides.^[29]^ We envisage that this process, in the tetrose cytidines, could furnish the selectivity required to synthesize the *threo*‐isomer (i.e. TNA cytidine) by selective destruction of the *erythro*‐isomer of thiocytidine.

β‐*erythro*‐thiocytidine **10** underwent highly selective urea‐mediated phosphorylation,[^23^, ^45^] with only one equivalent of ammonium dihydrogen phosphate, to yield 2′,3′‐cyclic phosphate **13** as the major product (Figure 5 A and Supplementary Figure 15). We anticipated that the C2‐sulfur of **13** would, after undergoing photoanomerization from β‐anomer to α‐anomer, attack the C2′‐position of the sugar moiety, leading to nucleotide degradation. Pleasingly, upon irradiation we observed rapid destruction of cyclic phosphate **13**; 2‐thiocytosine **12** was the sole observed cytosine product after irradiating **13** for 2 days (Supplementary Figure 18). We next irradiated a mixture of *erythro*‐cyclic phosphate **13** and *threo*‐**9**; after 3 days **8** was the major nucleoside (47 %), whilst **13** was observed to degrade in only 1 day (Figure 6 and Supplementary Figures 16 and 17), demonstrating that irradiation, when coupled with *erythro*‐specific 2′,3′‐cyclic phosphate synthesis, allows the photochemical selection of *threo*‐thiocytidine **8** from a mixture of *erythro*‐ and *threo*‐thiocytidines. The 2′,3′‐*trans*‐configuration of threose means that TNA nucleosides cannot form 2′,3′‐cyclic phosphates, and therefore TNA thiocytidine is inherently protected from these destructive reactions. Phosphorylation of β‐*threo*‐thiocytidine **9** cannot furnish β‐*threo*‐2′,3′‐cyclic phosphates, but reversibly yields a mixture of nucleoside phosphates (Figure 5 B and Supplementary Figure 20). Irradiation of this mixture of acyclic phosphates leads to anomerization of β‐*threo* to α‐*threo* nucleotides, which was unambiguously confirmed (following treatment with bovine intestinal phosphatase) by sample spiking with α‐*threo*‐thiocytidine **8** (Supplementary Figure 20). However, the high temperature required for urea‐mediated phosphorylation resulted in some conversion of **9** back to anhydrocytidine **6** and concomitant phosphorylation and thermal rearrangement to yield photostable α‐*erythro*‐cytidine‐2′,3′‐cyclic phosphate **14** (Supplementary Figure 20).

**Figure 5:**
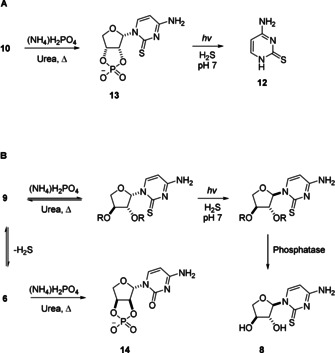
Photochemical selection of *threo*‐thiocytidine induced by *erythro*‐specific 2′,3′‐cyclic phosphate synthesis. A) Urea‐mediated phosphorylation of *erythro*‐thiocytidine **10** yields cyclic phosphate **13** in near quantitative yield. **13** is observed to undergo rapid and complete photodegradation to free base **12**. B) Urea‐mediated phosphorylation of β‐*threo*‐thiocytidine **9** yields a mixture of β‐*threo*‐thiocytidine phosphates that photoanomerize to their α‐anomers. Annulation of **9** is also observed during urea‐mediated phosphorylation to yield anhydronucleoside **6**, which phosphorylates and rearranges to yield photostable *erythro*‐cytidine **14**. R=H or (PO_3_^−^)_*n*_.

**Figure 6:**
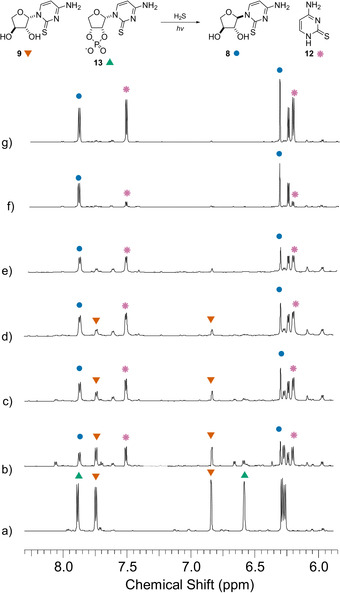
^1^H NMR (700 MHz, noesygppr1d, H_2_O/D_2_O (9:1), 5.85–8.3 ppm) spectra showing the irradiation (254 nm) of **9** (12 mm) and **13** (12 mm) at pH 7 with H_2_S (109 mm): a) before the irradiation; and after b) after 1 day; c) 2 days; d) 3 days; e) 4 days irradiation. Spectrum f) following addition of authentic **8**. Spectrum g) following addition of authentic **12**. (C6)−H and (C1′)−H resonances are labelled for **9** (▾), **13** (▴), and **8** (•). (C6)−H and (C5)−H resonances are labelled for **12** (✶).

Our results demonstrate the TNA monomers can be accessed by the same photochemical strategy as RNA monomers. We also observed that, following phosphorylation and irradiation, threose thiocytidine **8** can be photochemically selected. However, this selection is only feasible in the thiocytidine intermediates, as β‐*erythro*‐cytidines, such as **14**, are photostable. A milder phosphorylation strategy would be required to further enhance the observed selection in the intermediate thiocytidines and avoid conversion of **9** to **14**.^[46]^ However, the *trans*‐disposition of hydroxy groups in cytidine **3** means that phosphorylation cannot yield a 2′,3′‐phosphate, whereas the *cis*‐disposition of hydroxy groups in **11** (and its cytidine congener **15**) mean that 2′,3′‐phosphate formation is inevitable in *erythro*‐nucleotides. Therefore, the sluggish chemical reactivity of 2′,3′‐cyclic phosphates[^47^, ^48^, ^49^, ^50^, ^51^] may provide the final selection required, during polymer synthesis, to selectively incorporate TNA into nucleic acids. Whilst **3** can be chemically activated to polymerisation (for example, as an imidazolide[^52^, ^53^]), activation in the *erythro*‐isomer **15** will be quenched by formation of 2′,3′‐cyclic phosphate **14**. We have demonstrated that prebiotic photoanomerization can selectively furnish TNA cytidine, and our results warrant further investigation of selective TNA synthesis in the context of the origins of life.

## Conflict of interest

The authors declare no conflict of interest.

## Supporting information

As a service to our authors and readers, this journal provides supporting information supplied by the authors. Such materials are peer reviewed and may be re‐organized for online delivery, but are not copy‐edited or typeset. Technical support issues arising from supporting information (other than missing files) should be addressed to the authors.

Supplementary
