# Supplementary material for: Selective Prebiotic Synthesis of α‐Threofuranosyl Cytidine by Photochemical Anomerization
Source: Angew Chem Int Ed Engl. 2021 Mar 26;60(19):10526–30. doi: 10.1002/anie.202101376 (PMC8252090; doi:10.1002/anie.202101376)
Supplement: Supplementary file 1 — Supplementary [file ANIE-60-10526-s001.pdf]

Angewandte Chemie
Eine Zeitschrift der Gesellschaft Deutscher Chemiker

Supporting Information

Selective Prebiotic Synthesis of α-Threofuranosyl Cytidine by Photochemical Anomerization

*Ben W. F. Colville and Matthew W. Powner**

anie_202101376_sm_miscellaneous_information.pdf

Table of Contents

General experimental 4

General Methods 5

Phosphorylation Method 1 5

Phosphorylation Method 2 5

Irradiation Method 1 5

Phosphatase Method 1 5

Supplementary Figures 6

Reaction of TAO and EAO with cyanoacetylene 6

Thiolysis of tetro-2-2′-anhydrocytidine **6** and **7** 7

Reaction of β-*threo* **9** and β-*erythro*-thiocytidine **10** with hydrogen peroxide 8

Hydrogen peroxide oxidation of α-*threo*-**8** 9

Reaction of β-threo-**9** and α-threo-thiocytidine **8** with hydrogen peroxide 10

Photoanomerization of β-*threo*-**9** 11

Photoanomerization of β-*threo*-**9** and β-*erythro*-**10** 13

Photoanomerization of β-*threo*-**9** and β-*erythro*-**10** spiked with authentic α-threo-**8** α-erythro-**11** 14

Photoanomerization of β-*threo*-**9** and β-*erythro*-**10** spiked with authentic α-*threo*-**8** α-*erythro*-**11** 15

Irradiation of α-*threo*-**8** 16

Irradiation of α-*erythro*-**11** 18

Irradiation of α-*threo*-**8** and α-*erythro*-**11** 19

Phosphorylation of β-*erythro*-**10** 20

Irradiation of β-*threo*-**9** and β-*erythro*-**13** 21

Irradiation of β-*erythro*-**13** 23

Phosphorylation and subsequent irradiation of β-*threo*-**9** and treatment by phosphatase 24

Synthesised Compounds 26

Tetrose-aminooxazolines 26

Tetrofuranosyl-2,2′-anhydrocytdine hydrochloride (**6/7**) 29

Synthesis of 2-thiocytidine tetroses **9** and **10** in formamide 32

Two step synthesis of β-*tetro*-thiocytidines **9** and **10** 33

Characterisation data for β-*threo*-**9** 34

Characterisation data for β-*erythro*-**10** 37

Synthetic route for authentic standard of α-*threo*-**8** 40

Synthesis of Calcium L-threonate 41

Synthesis of L-threonolactone 44

Synthesis of 2′,3′-di-*O*-benzoyl-threonolactone 47

Silylation of 2-thiocytosine **12** 50

Synthesis of 2′,3′-di-*O*-benzoyl-2-thio-threocytidine 51

Synthesis of authentic standard of α-*threo*-thiocytidine **8** 55

Synthetic route for authentic standard of α-*erythro*-**11** 58

Synthesis of 2′,3′-di-*O*-benzoyl-erythronolactone 59

Synthesis of 2′,3′-di-*O*-benzoyl-erythrothiocytidine 62

Synthesis of α-*erythro*-thiocytidine **11** 66

Synthesis of β-*erythro*-thiocytidine-2′,3′-cyclic phosphate **13** 69

References 72

General experimental

All compounds were obtained from *Sigma Aldrich*, *Alfa Aesar*, *Hopkins and Williams*, *Fisher Scientific*, *Carbosynth*, *BDH*, *Lancaster* and *VWR* and used without further purification unless specified. Water (H_2O) refers to deionised water produced by an *Elga Option 3* purification system. Flash Column Chromatography (FCC) and Reverse Phase Flash Column Chromatography (RP-FCC) were carried out using either *Biotage* SNAP or *Kinesis* TELOS cartridges in a *Biotage Isolera One* purification system. Sodium hydrogen sulfide (NaSH) was purchased from *Sigma Aldrich* as a hydrate, NaSH.xH_2O, and used without further purification. After reactions were complete, solutions were sparged of H_2S by bubbling argon or nitrogen gas for 1h+ through the solution, whilst maintaining the pH of the solution at acidic pH to ensure efficient degassing and H_2S was quenched with sodium hypochlorite solution in 2 or 3 glass bubblers. Reactions using with anhydrous solvents were carried out in flame dried glassware that was cooled under N_2 with a Schlenk adapter. ^{1}H, ^{13}C and ^{31}P NMR spectra were recorded on Bruker NMR spectrometers AVANCE Neo 700, AVANCE III 600, AVANCE III 400 and AVANCE 300, equipped with a Bruker room temperature 5 mm multinuclear gradient probe (700 MHz), 5 mm DCH cryoprobe (600 MHz) and a gradient probe (400 and 300 MHz). All reported chemical shifts (δ) are given in parts per million (ppm) relative to residual solvent peaks, and ^{1}H and ^{13}C spectra calibrated using the residual solvent peaks relative shift to TMS. Water supressed ^{1}H NMR spectra were obtained using a 1D nuclear Overhauser enhancement spectroscopy (NOESY) pulse sequence (noesygppr1d, *Bruker*) and all spectra were recorded at 298 K. Rapidly exchanging proton (O–H, N–H) resonances are not detected due to signal broadening and coalescence with the HOD signal in 9:1 H_2O/D_2O. Coupling constants (*J*) are given in Hertz (Hz). The following abbreviations refer to spin multiplicities: s (singlet); d (doublet); t (triplet); q (quartet); m (multiplet); br (broad signal); obs. (obscured/coincidental signals), app. (apparent) or any combination of these. Diastereotopic geminal (AB) spin systems coupled to an additional nucleus are reported as ABX. NMR data are stated as follows: chemical shift (multiplicity, coupling constants (*J*), number of protons, nuclear assignment). An electrothermal standard digital apparatus was used to record the melting points for all crystalline solids. Melting points are uncorrected and are quoted to the nearest C. Infrared spectra (IR) were recorded on a *Shimadzu IR Tracer 100* FT-IR spectrometer. Absorption maxima are reported in wavenumber (cm^{-1}). Ultraviolet (UV) spectra were recorded on a *Shimadzu UV-1800* UV spectrophotometer. Absorption maxima are reported in wavelength (nm). *Mettler Toledo Seven Compact* pH meter with a *Mettler Toledo InLab* semi-micro pH probe and a *Corning pH meter 430* with a *Fischerbrand* (FB68801) semi-micro pH probe were used to measure the pH of solutions. UV irradiations were performed using a *Rayonet RPR-200* with *RPR-3000A* lamps or with *RPR-2547A* lamps or using an *Ultra-Violet Products Ltd (UVP, LLC) Pen-Ray* 254 nm mercury lamp in a water-cooled *Ace Glassware* quartz microreactor. Mass spectra and

accurate mass measurements were recorded by *Thermo Finnigan MAT 900XP*, *VG70-SE*, *Waters LCT Premier XE Q-TOF or Thermo Orbitrap Q Exactive Plus* instruments at the Department of Chemistry, University College London.

General Methods

Phosphorylation Method 1

Adapted from *Stairs et al.*[1]

Nucleosides (0.01 mmol), ammonium phosphate (1, 2, 4 equivalents) and urea (1.6 mmol) were heated to 140 °C for the specified time (5, 10 or 30 minutes) and then allowed to cool to room temperature. The resultant solids were dissolved in D_2O (2 mL) and lyophilised. The lyophilisate was dissolved in D_2O and analysed by ^1H and ^{31}P NMR.

Phosphorylation Method 2

Nucleosides (0.01 mmol), ammonium phosphate (1, 2 or 4 equivalents) and urea (1.6 mmol) placed under an inert gas atmosphere (N_2 or Ar) and heated to 140 °C for the specified time (5, 10 or 30 minutes) and then allowed to cool to room temperature. The resultant solids were dissolved in D_2O (2 mL) and lyophilised. The lyophilisate was redissolved in D_2O and analysed by NMR spectroscopy.

Irradiation Method 1

Nucleosides/nucleotides (0.022 mmol) and NaSH (3 mg, 0.03 mmol) were dissolved in degassed water (0.5/1 mL, containing 10% D_2O). The solution was adjusted to pH 6.5 or 7 with HCl, transferred to a quartz NMR tube and irradiated with a mercury lamp (principle emission at 254 nm) for the desired duration. The reaction was periodically monitored by NMR spectroscopy.

Phosphatase Method 1

Nucleotides were dissolved in H_2O/D_2O (0.5/1 mL), then NaCl (5.9 mg) and $MgCl_2$ (2.0 mg) were added and the solution was adjusted to pH 8.0. Bovine intestinal phosphatase (10 µl, *Sigma Aldrich*) was added, and the solution maintained at 37 °C for 24 hours. The reaction was then lyophilized. The lyophilisate was dissolved in D_2O (0.5 mL) and analysed by NMR spectroscopy and sample spiking.

Supplementary Figures

Reaction of **TAO** and **EAO** with cyanoacetylene

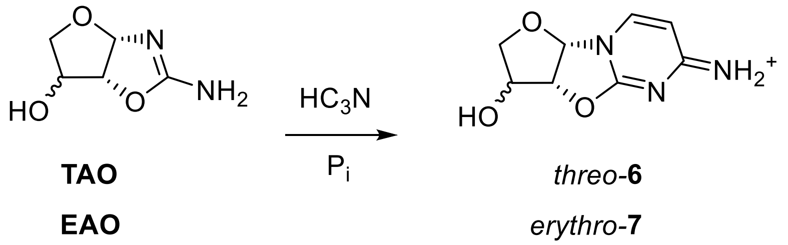

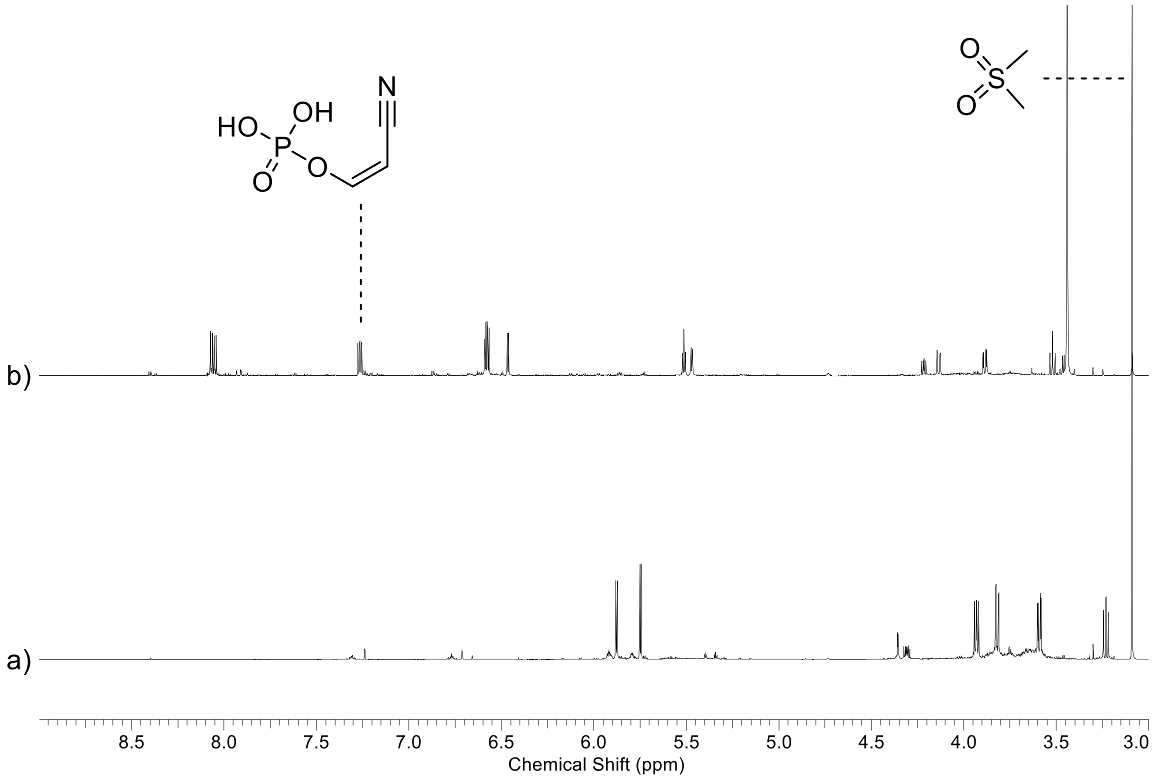

Supplementary Figure 1 *^{1}H NMR (700 MHz, H_2O/D_2O (9:1), noesygppr1d, 3.0-9.0 ppm) spectra to show: a) threose aminooxazoline **TAO** and erythrose aminooxazoline **EAO** (123 mM) with methylsulfonylmethane (MSM, 50 mM) internal standard; b) the reaction of a) with cyanoacetylene (1 M, 1 mL) and sodium phosphate (P_i, 67 mM) at pH 6.5, room temperature after 1 day. The reaction proceeds with near quantitative conversion (98%) by NMR spectroscopy.*

Thiolysis of tetro-2-2′-anhydrocytidine **6** and **7**

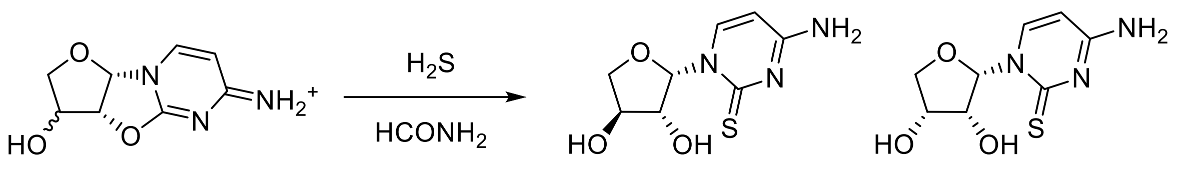

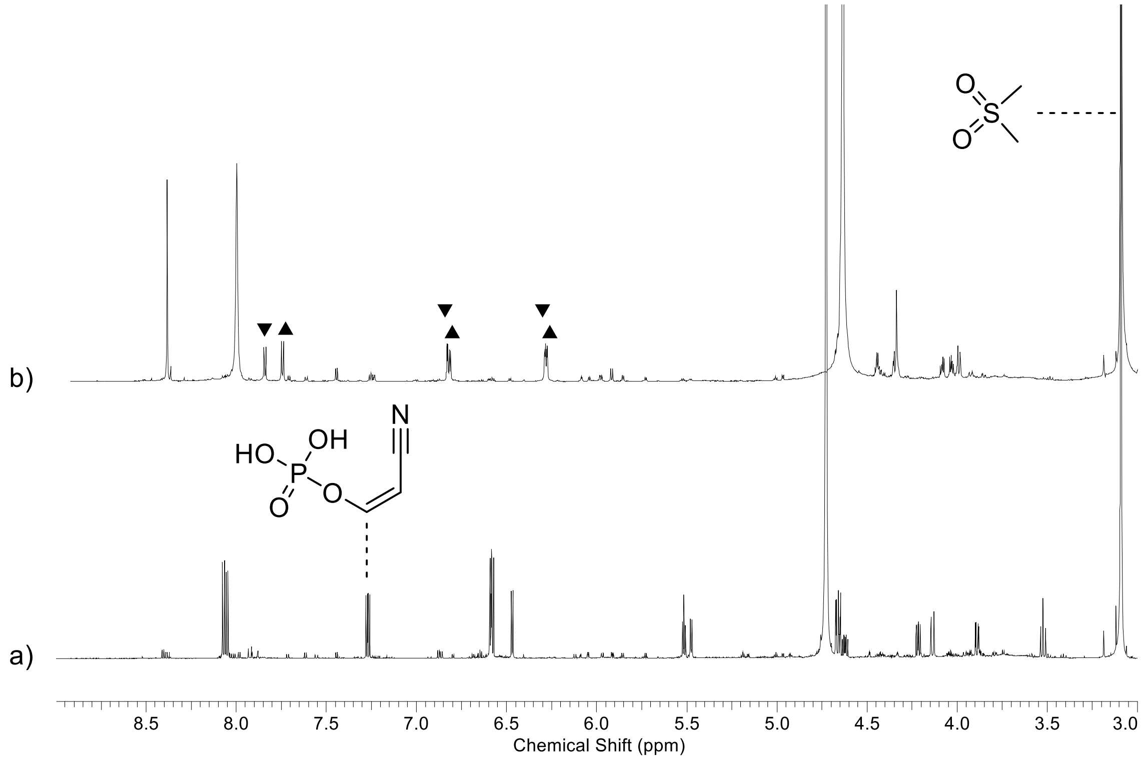

***Supplementary Figure 2** ^{1}H NMR (700 MHz, D_2O, zg30, 3.0-9.0 ppm) spectra to show: a) threo-anhydrocytidine **6** and erythro-anhydrocytidine **7** (49 mM); b) the products of the reaction of a) with NaSH (635 mM) in formamide (1 mL) at room temperature overnight after then being lyophilized and dissolved in D_2O. The products β-threo-2-thiocytidine **9** and β-erythro-2-thiocytidine **10** (78% yield) are labelled, as well as the internal standard methylsulfonylmethane (MSM, 3.09 ppm) which was used to quantify the reaction yield. (C6)–H, (C5)–H and (C1′)–H protons are labelled: β-threo-2-thiocytidine **9** (▼) and β-erythro-2-thiocytidine **10** (▲).*

Reaction of β-*threo* **9** and β-*erythro*-thiocytidine **10** with hydrogen peroxide

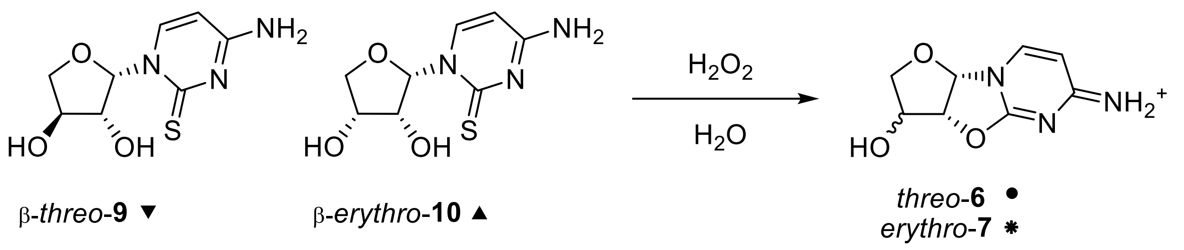

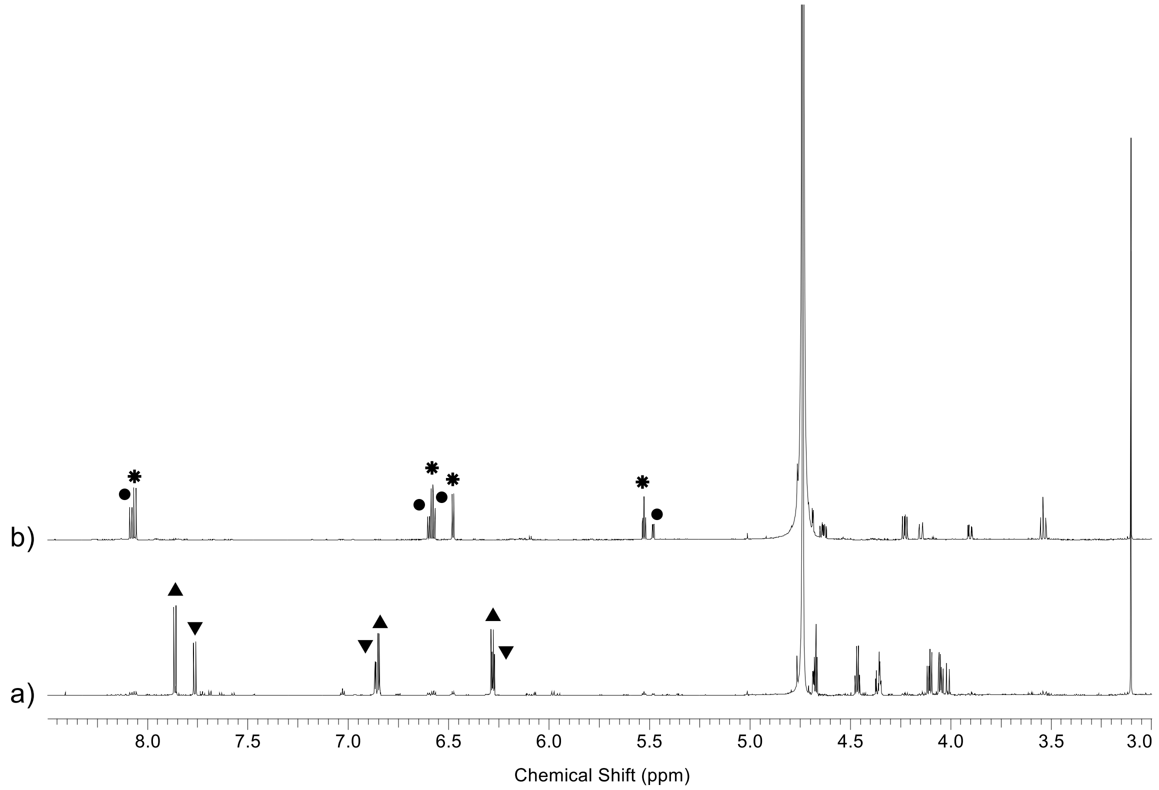

Supplementary Figure 3 *^{1}H NMR (700 MHz, D_2O, zg30, 3.0-8.5 ppm) spectra to show: a) β-threo-**9** and β-erythro-**10** (17 mM); b) the reaction of a) with hydrogen peroxide (98 mM) at room temperature after 10 mins. (C6)–H, (C5)–H and (C1')–H protons for **6, 7, 9** and **10** (and (C2')–H protons for **6** and **7**) are labelled: β-threo-**9** (▼), β-erythro-**10** (▲), threo-**6** (●) and erythro-**7** (✱).*

Hydrogen peroxide oxidation of α-*threo*-**8**

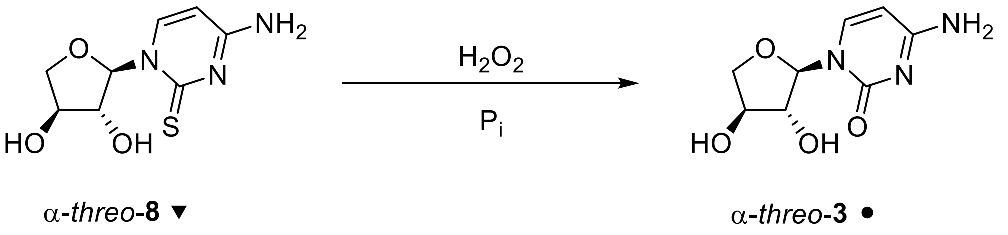

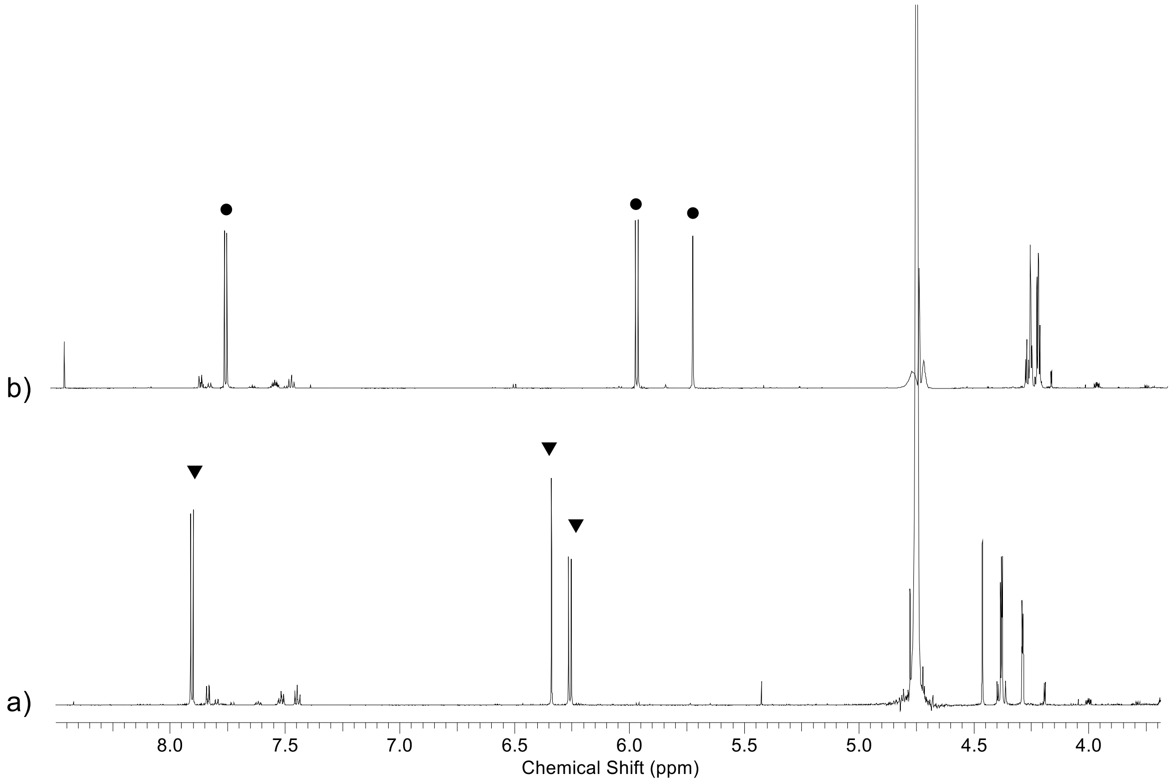

Supplementary Figure 4 *^{1}H NMR (700 MHz, H_2O/D_2O 9:1, (a) zg30, b) noesygppr1d), 3.7-8.5 ppm) spectra to show: a) α-threo-**8** (20 mM); b) the reaction of a) with hydrogen peroxide (59 mM) after 10 mins. (C6)–H, (C5)–H and (C1')–H protons are labelled: α-threo-**8** (▼), α-threo-**3** (●).*

Reaction of β-*threo*-**9** and α-*threo*-thiocytidine **8** with hydrogen peroxide

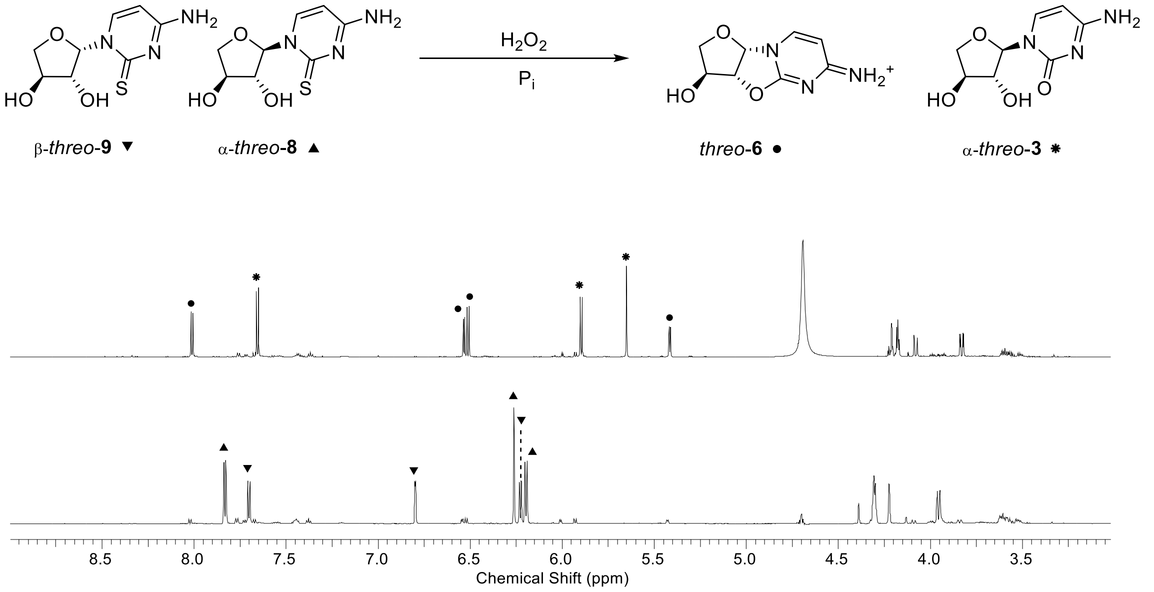

Supplementary Figure 5 *^{1}H NMR (700 MHz, H_2O/D_2O (9:1), Robust5_ver_03b, 3.0-9.0 ppm) spectra to show: a) β-threo-**9** (12.6 mM) and α-threo-**8** (18 mM); b) the reaction of a) with hydrogen peroxide (0.09 mmol, 196 mM) after 12 h. (C6)–H, (C5)–H and (C1')–H protons (and (C2')–H proton for threo-**6**) are labelled: β-threo-**9** (▼), α-threo-**8** (▲), threo-**6** (●), α-threo-**3** (✱).*

Photoanomerization of β-*threo*-**9**

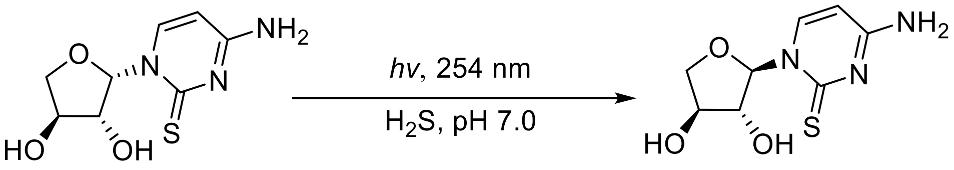

β-*threo*-**9** ▼   α-*threo*-**8** ✱

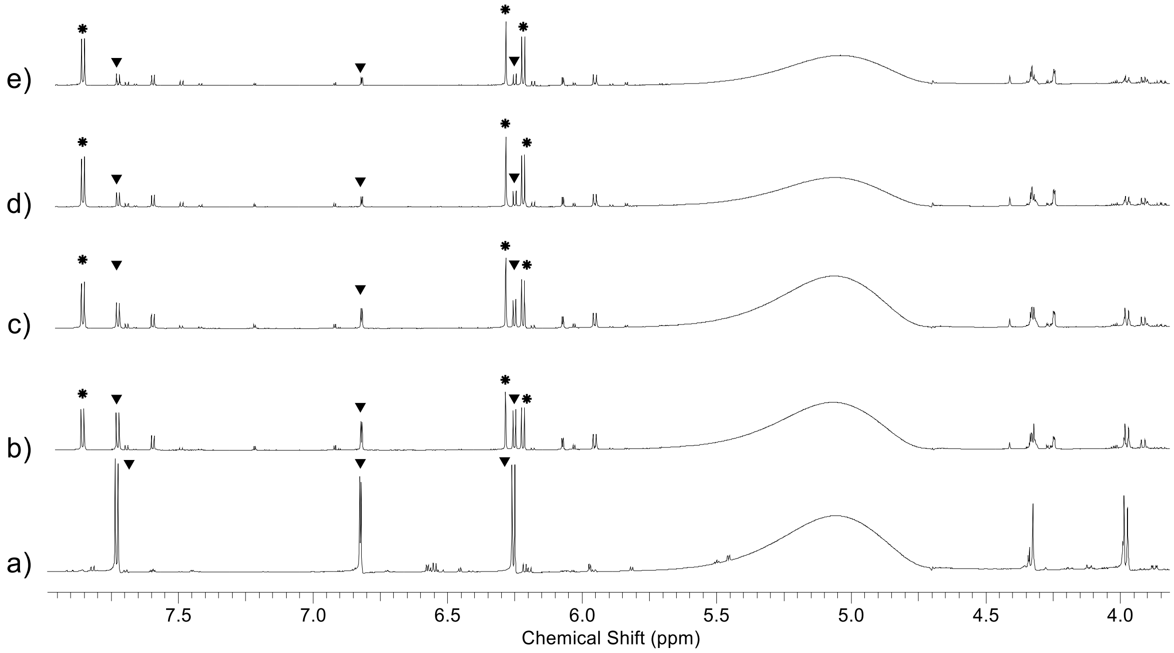

Supplementary Figure 6 *^{1}H NMR (700 MHz, H_2O/D_2O (9:1), Robust5_ver_03b, 3.8-8.0 ppm) spectra to show the irradiation (254 nm) of β-threo-thiocytidine* ***9*** *(25 mM) in H_2O/D_2O (9:1) at pH 7 with NaSH (0.013 mmol, 26 mM), MSM internal standard (73 mM, resonance at 3.09 ppm omitted): a) before irradiation; b) after 16 h of irradiation (43%); c) after 1 day of irradiation (46%); d) after 1.5 days of irradiation (51%); e) after 2 days of irradiation (51%). (C6)–H, (C5)–H and (C1′)–H protons are labelled: β-threo-**9** (▼), α-threo-**8** (✱).*

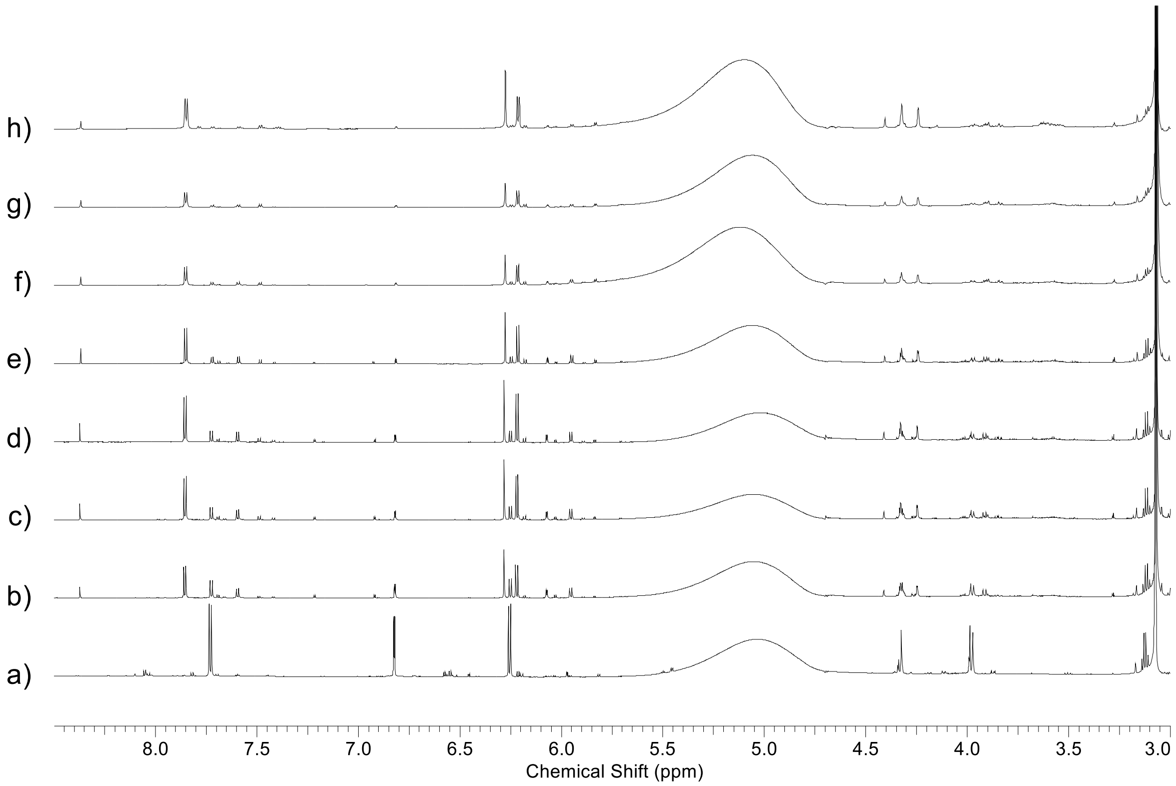

Supplementary Figure 7 *^{1}H NMR (700 MHz, H_2O/D_2O (9:1), Robust5_ver_03b, 3.0-8.5 ppm) spectra to show the irradiation (254 nm) of β-threo-thiocytidine* ***9*** *(25 mM) in H_2O/D_2O (9:1) at pH 7 with NaSH (0.013 mmol, 26 mM), MSM internal standard (73 mM, resonance at 3.09 ppm): a) before irradiation, b) after 1 day of irradiation (46%), c) after 1.5 days of irradiation (51%), d) after 2 days of irradiation (51%), e) after 2.5 days of irradiation (49%), f) after 3 days of irradiation (49%), g) after 3.5 days of irradiation (43%) and h) after spiking g) with authentic α-threo-**8**.*

Photoanomerization of β-*threo*-**9** and β-*erythro*-**10**

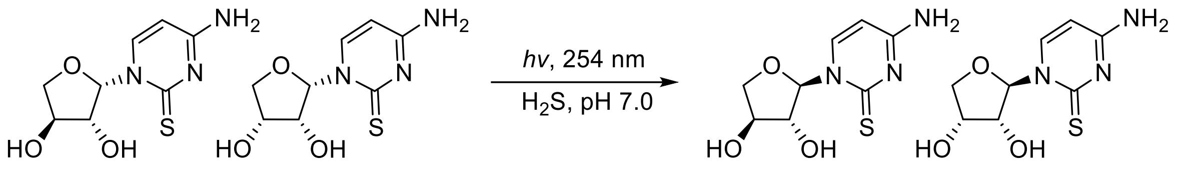

β-*threo*-**9** ▼ β-*erythro*-**10** ▲ α-*threo*-**8** ● α-*erythro*-**11** ❋

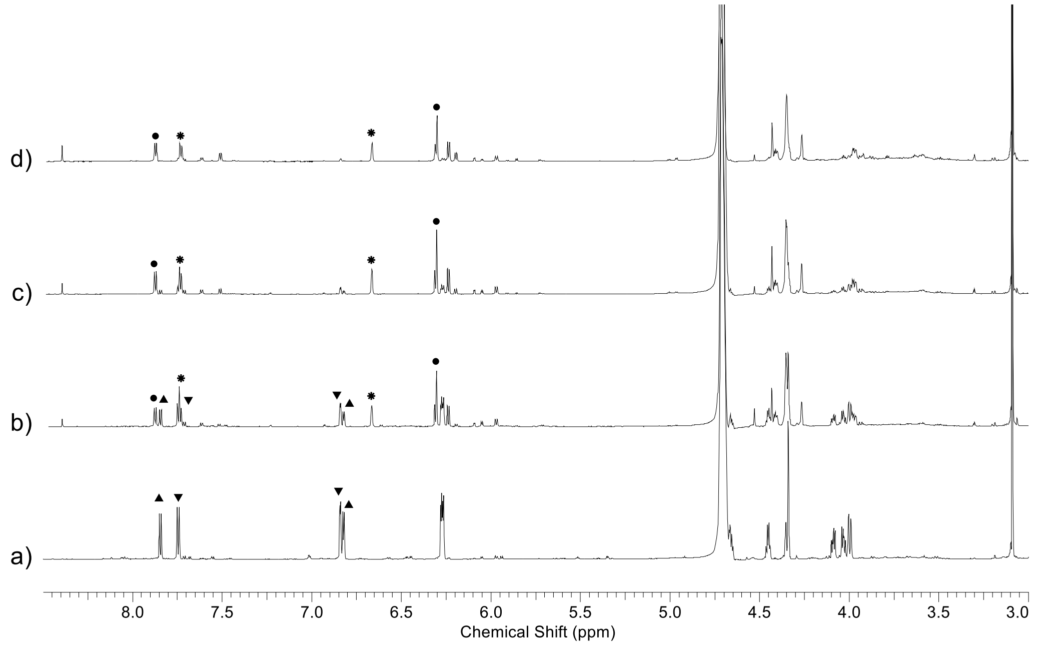

Supplementary Figure 8 *^{1}H NMR (700 MHz, H_2O/D_2O (9:1), noesygppr1d, 3.0-8.5 ppm) spectra to show the irradiation (254 nm) of 18 mM threo-**9** and 16 mM erythro-**10** in H_2O/D_2O (9:1) at pH 7 with NaSH (0.1 mmol, 113 mM), MSM internal standard (18.6 mM, resonance at 3.09 ppm): a) before the irradiation; b) after 1 day of irradiation (**8** (40%), **11** (42%)); c) after 2 days of irradiation (**8** (52%), **11** (51%)); d) after 3 days of irradiation (**8** (40%), **11** (36%)). (C6)–H and (C1')–H protons are labelled: β-threo-**9** (▼), β-erythro-**10** (▲), α-threo-**8** (●), α-erythro-**11** (❋).*

Photoanomerization of β-*threo*-**9** and β-*erythro*-**10** spiked with authentic α-*threo*-**8** α-*erythro*-**11**

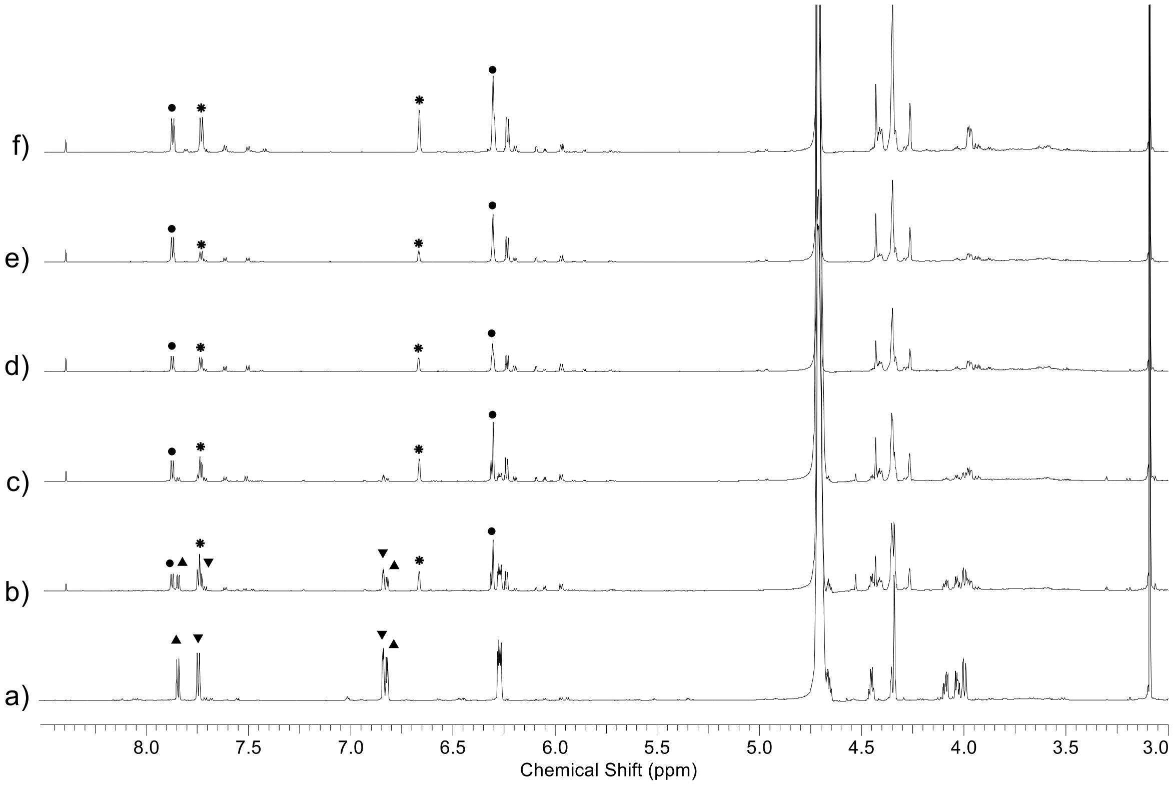

***Supplementary Figure 9** ^{1}H NMR (700 MHz, H_2O/D_2O (9:1), noesygppr1d, 3.0-8.5 ppm) spectra to show the irradiation (254 nm) of 18 mM threo-**9** and 16 mM erythro-**10** in H_2O/D_2O (9:1) at pH 7 with NaSH (0.1 mmol, 113 mM), MSM internal standard (18.6 mM, resonance at 3.09 ppm): a) before the irradiation; b) after 1 day of irradiation; c) after 2 days of irradiation; d) after 3 days of irradiation; e) after 3 days of irradiation and spiked with authentic α-threo-**8** and f) spiked with authentic α-erythro-**11**. (C6)–H and (C1')–H protons are labelled: β-threo-**9** (▼), β-erythro-**10** (▲), α-threo-**8** (●), α-erythro-**11** (❋).*

Photoanomerization of β-*threo*-**9** and β-*erythro*-**10** spiked with authentic α-*threo*-**8** α-*erythro*-**11**

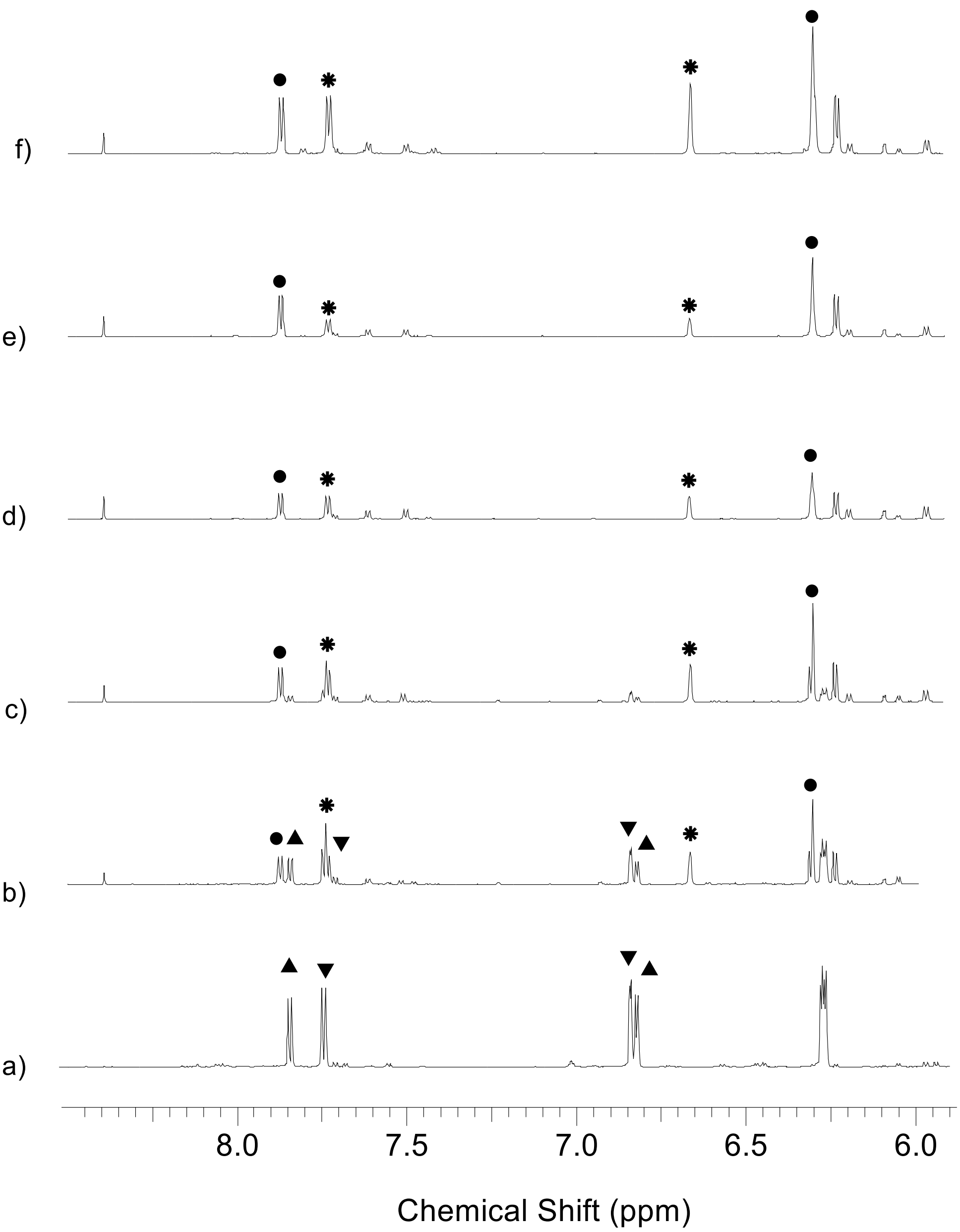

Supplementary Figure 10 *^{1}H NMR (700 MHz, H_2O/D_2O (9:1), noesygppr1d, 5.9-8.5 ppm) spectra to show the irradiation (254 nm) of 18 mM threo-**9** and 16 mM erythro-**10** in H_2O/D_2O (9:1) at pH 7 with NaSH (0.1 mmol, 113 mM), MSM internal standard (18.6 mM, resonance at 3.09 ppm omitted): a) before the irradiation; b) after 1 day of irradiation; c) after 2 days of irradiation; d) after 3 days of irradiation; e) after 3 days of irradiation and spiked with authentic α-threo-**8** and f) spiked with authentic α-erythro-**11**. (C6)–H and (C1')–H protons are labelled: β-threo-**9** (▼), β-erythro-**10** (▲), α-threo-**8** (●), α-erythro-**11** (❋).*

Irradiation of α-*threo*-**8**

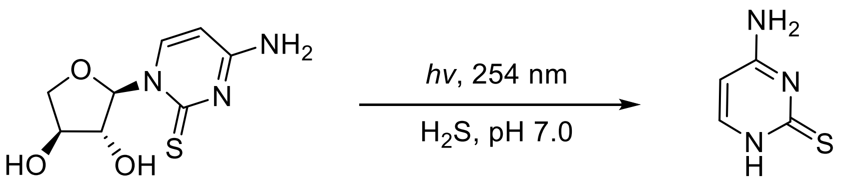

α-*threo*-**8** ▼ **12** ✱

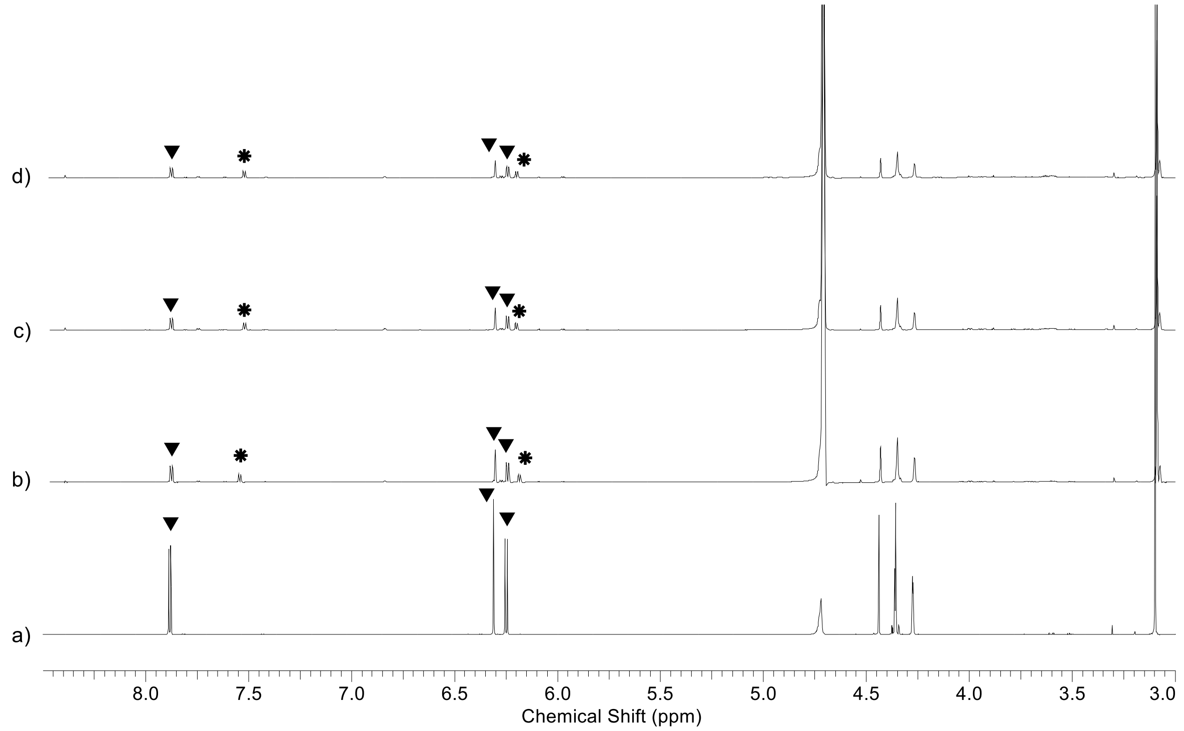

Supplementary Figure 11 *^{1}H NMR (700 MHz, H_2O/D_2O (9:1), noesygppr1d, 3.0-8.5 ppm) spectra to show the irradiation (254 nm) of α-threo-**8** (41 mM) in H_2O/D_2O (9:1) at pH 7 with NaSH (0.11 mmol, 227 mM), MSM internal standard (35 mM, resonance at 3.09 ppm): a) before the irradiation; b) after 1 day of irradiation; c) after 2 days of irradiation and d) after 3 days of irradiation. (C6)–H, (C5)–H and (C1')–H protons (where applicable) are labelled: α-threo-**8** (▼), 2-thiocytosine **12** (✱).*

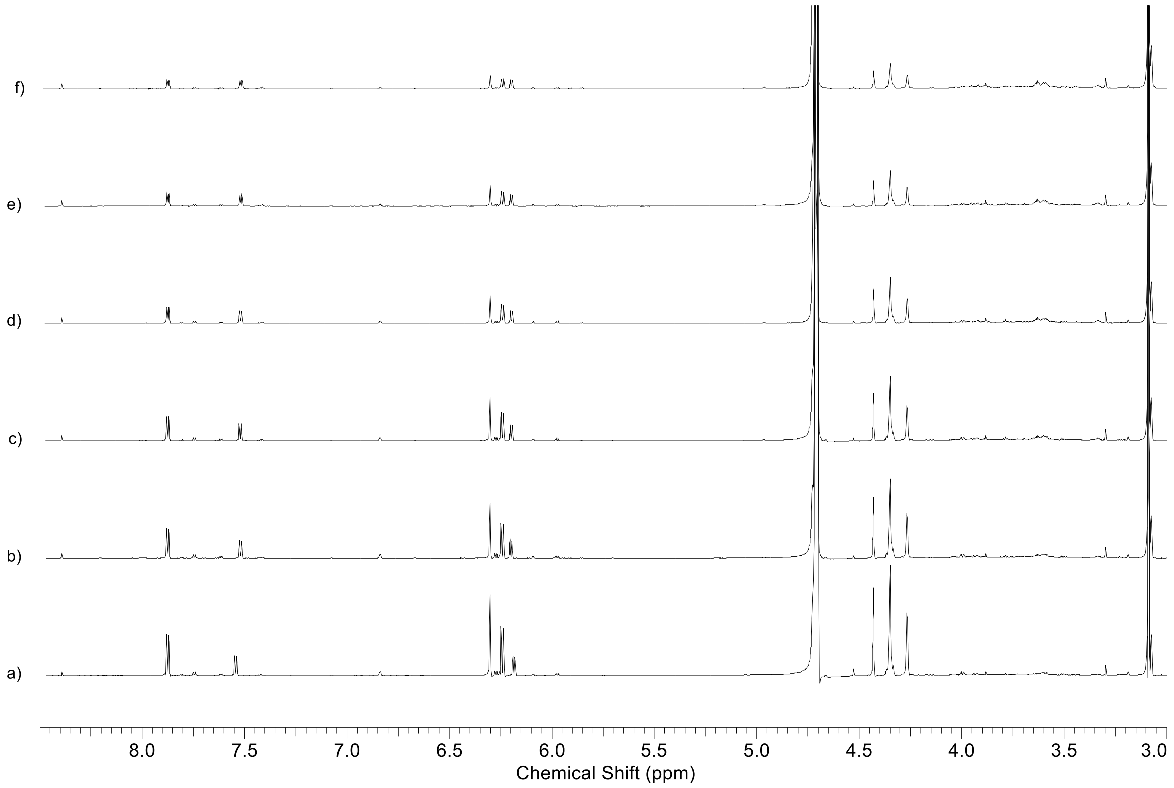

Supplementary Figure 12 *^{1}H NMR (700 MHz, H_2O/D_2O (9:1), noesygppr1d, 3.0-8.5 ppm) spectra to show the irradiation (254 nm) of α-threo-**8** (41 mM) in H_2O/D_2O (9:1) at pH 7 with NaSH (0.11 mmol, 227 mM), MSM internal standard (35 mM, resonance at 3.09 ppm): a) after 1 day of irradiation; b) after 2 days of irradiation; c) after 3 days of irradiation; d) after 4 days of irradiation; e) after 5 days of irradiation and f) after 6 days of irradiation.*

Irradiation of α-*erythro*-**11**

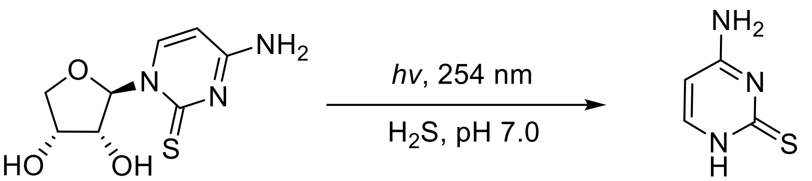

α-*erythro*-**11** ▲ **12** ✱

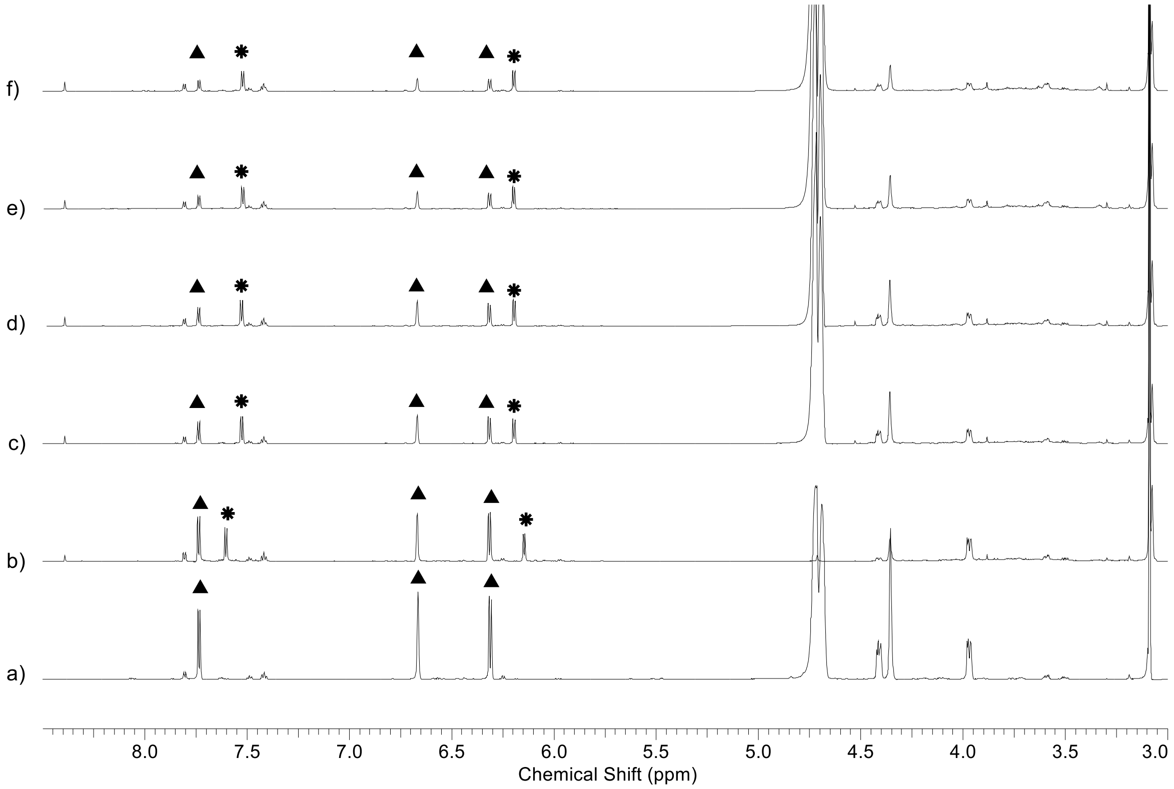

Supplementary Figure 13 *^{1}H NMR (700 MHz, H_2O/D_2O (9:1), noesygppr1d, 3.0-8.5 ppm) spectra to show the irradiation (254 nm) of α-erythro-**11** (41 mM) in H_2O/D_2O (9:1) at pH 7 with NaSH (0.12 mmol, 248 mM), MSM internal standard (43 mM, resonance at 3.09 ppm): a) before the irradiation; b) after 1 day of irradiation; c) after 2 days of irradiation; d) after 3 days of irradiation; e) after 4 days of irradiation and f) after 5 days of irradiation. (C6)–H, (C5)–H and (C1')–H protons (where applicable) are labelled: α -erythro-**11** (▲), 2-thiocytosine **12** (✱).*

Irradiation of α-*threo*-**8** and α-*erythro*-**11**

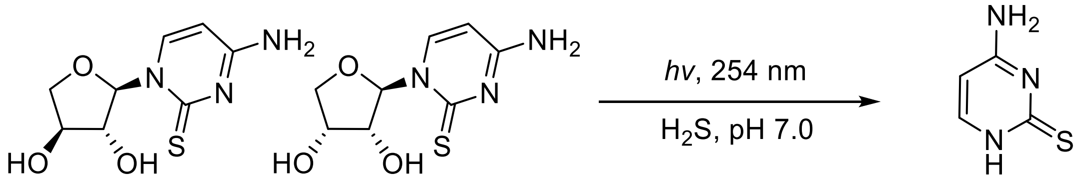

α-*threo*-**8** ▼ α-*erythro*-**11** ▲ **12** ✱

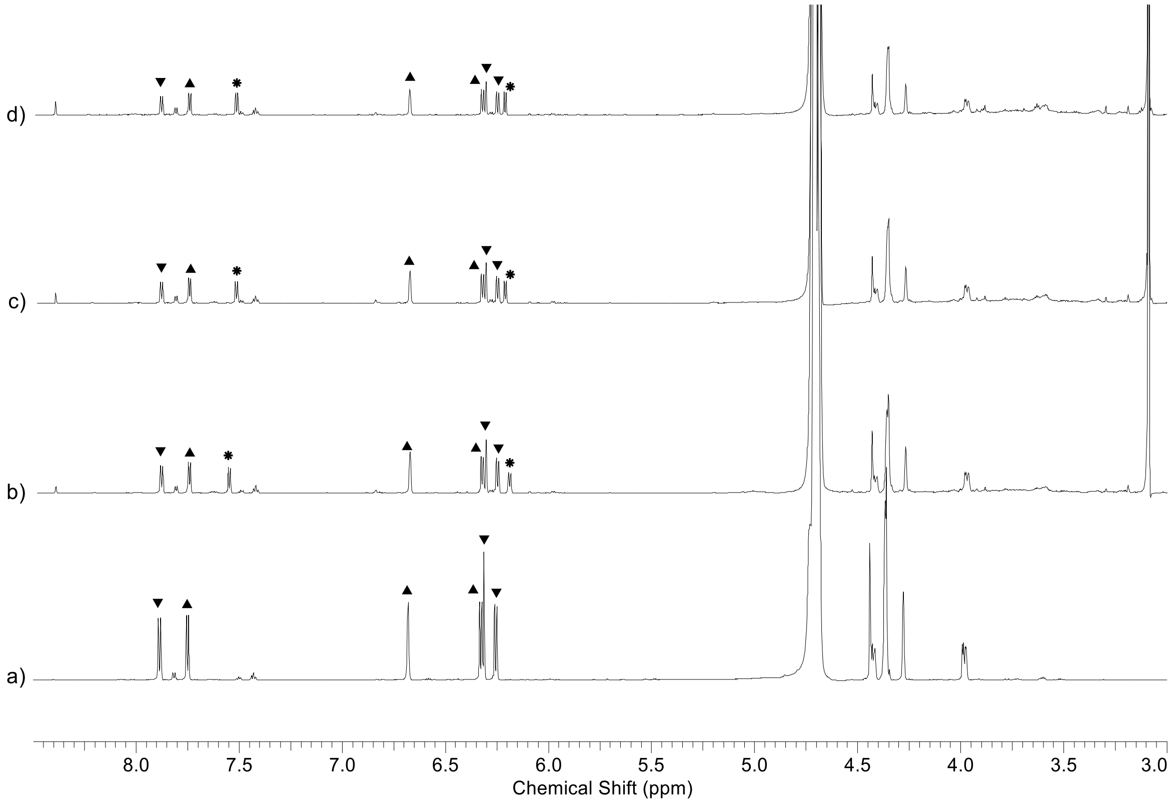

***Supplementary Figure 14** ^{1}H NMR (700 MHz, H_2O/D_2O (9:1), noesygppr1d, 3.0-8.5 ppm) spectra to show the irradiation at 254 nm of α-threo-**8** and α-erythro-**11** (6.5 mM threo: 6.8 mM erythro) in H_2O/D_2O (9:1) at pH 7 with NaSH (0.14 mmol, 139 mM), MSM internal standard (13.3 mM, resonance at 3.09 ppm): a) before the irradiation; b) after 1 day of irradiation; c) after 2 days of irradiation and d) after 3 days of irradiation. (C6)–H, (C5)–H and (C1')–H protons (where applicable) are labelled: α -threo-**8** (▼), α-erythro-**11** (▲), 2-thiocytosine **12** (✱).*

Phosphorylation of β-*erythro*-**10**

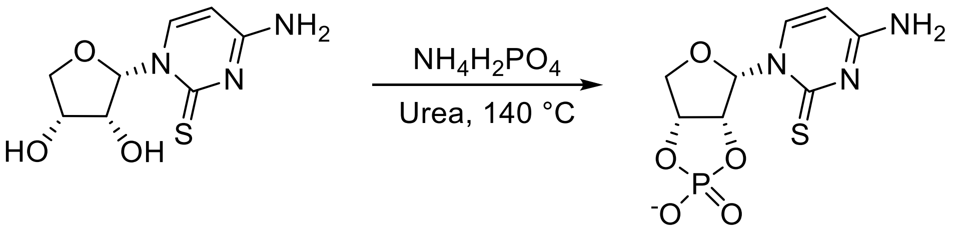

β-*erythro*-**10** β-*erythro*-**13**

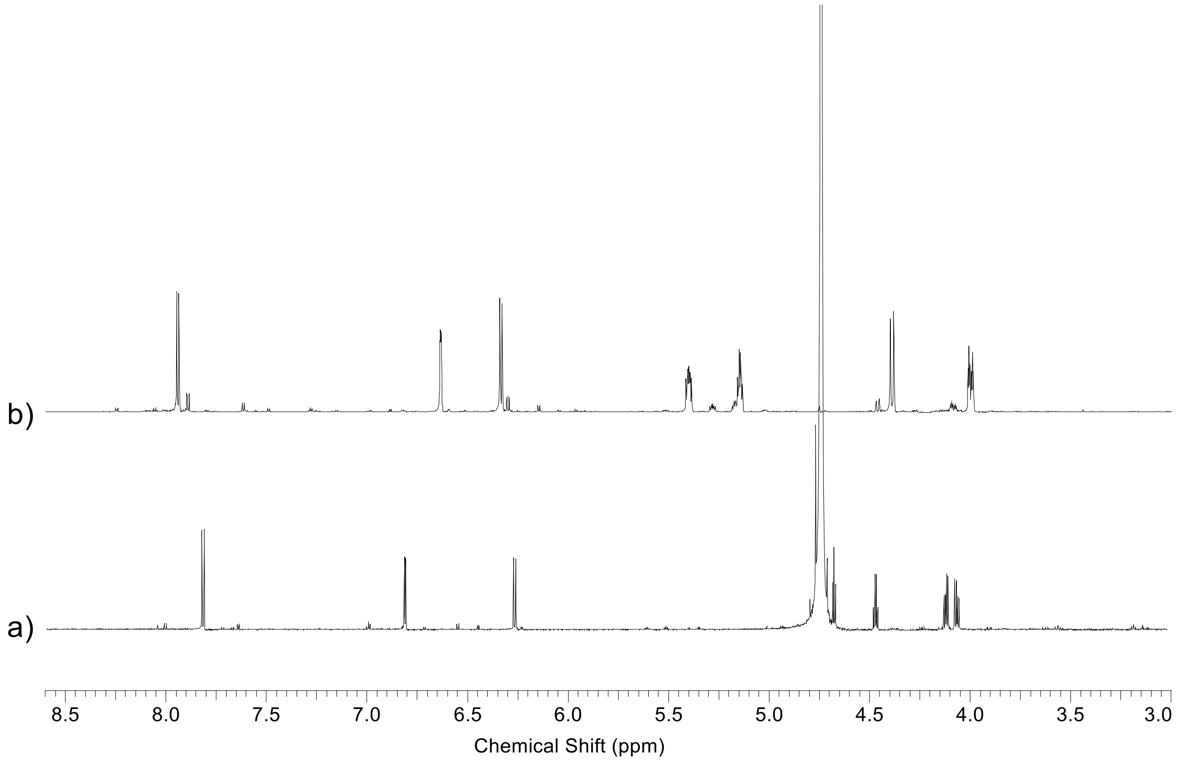

Supplementary Figure 15 *^{1}H NMR (700 MHz, D_2O, (a) zg30, b) lc1pngpf2) 3.0-8.52 ppm) spectra to show the phosphorylation of β-erythro-**10** (0.06 mmol) with ammonium phosphate (0.06 mmol): a) β-erythro-**10**; b) crude products of the reaction of β-erythro-**10** under Phosphorylation Method 1.*

Irradiation of β-*threo*-**9** and β-*erythro*-**13**

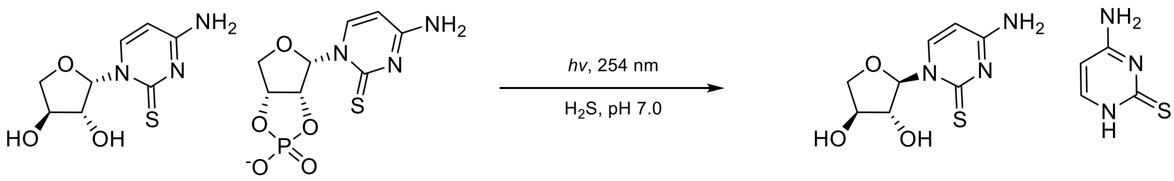

β-*threo*-**9** ▼ β-*erythro*-**13** ▲ α-*threo*-**8** ● **12** ✱

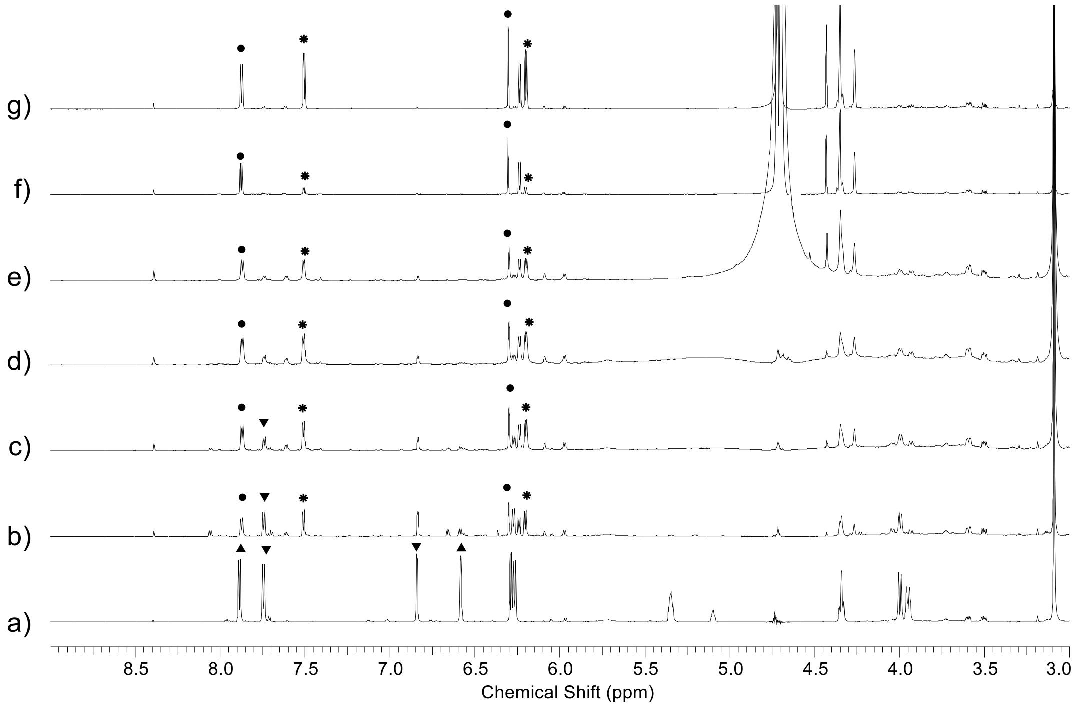

Supplementary Figure 16 *^{1}H NMR (700 MHz, H_2O/D_2O (9:1), noesygppr1d, 3.0-9.0 ppm) spectra to show the irradiation at 254 nm of 12 mM threo-**9** and 12 mM β-2′,3′-cyclic-phosphate-erythro-**13** in H_2O/D_2O (9:1) at pH 7 with NaSH (0.11 mmol, 109 mM), MSM internal standard (18.1 mM, resonance at 3.09 ppm): a) before the irradiation; b) after 1 day of irradiation (**8** (33%), **12** (36%)); c) after 2 days of irradiation (**8** (35%), **12** (38%)); d) after 3 days of irradiation (**8** (47%), **12** (52%)); e) after 4 days of irradiation (**8** (29%), **12** (30%)); f) after 4 days of irradiation and spiked with authentic α-threo-**8** and g) spiked with authentic 2-thiocytosine **12**. (C6)–H, (C1′)–H and (C5)–H (for **12**) protons are labelled: β-threo-**9** (▼), β-2′,3′-cyclic-phosphate-erythro-**13** (▲), α-threo-**8** (●), 2-thiocytosine **12** (✱).*

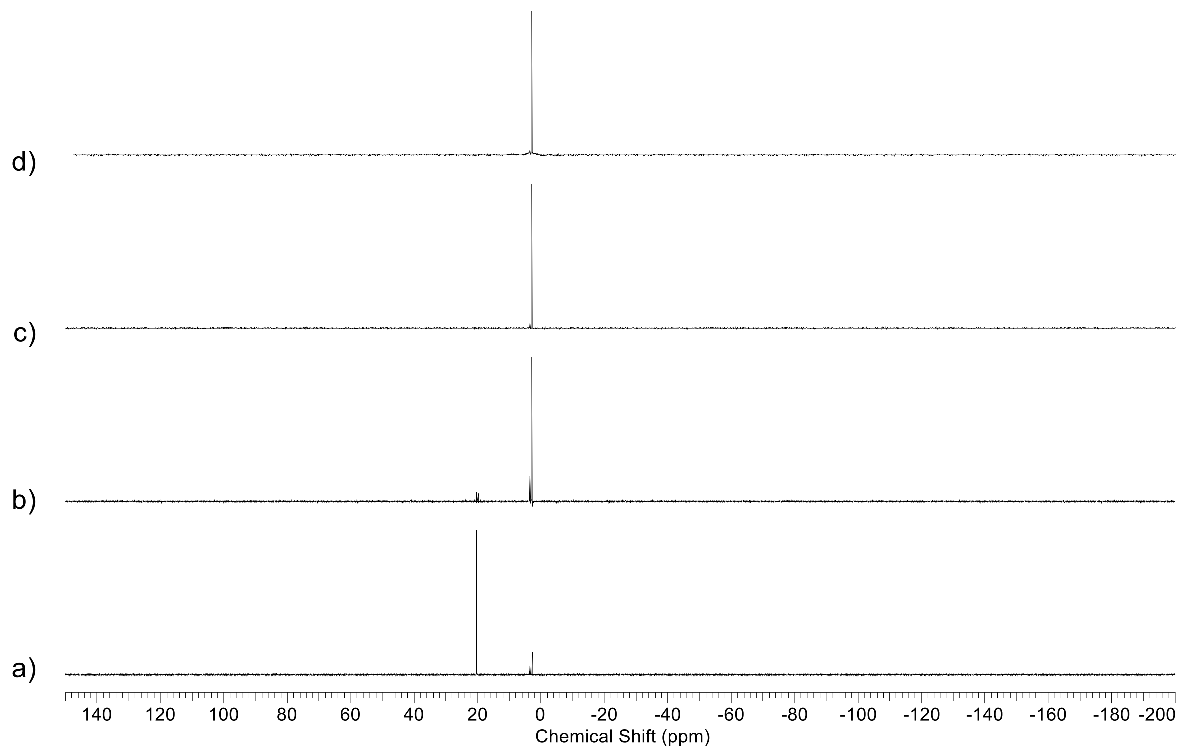

Supplementary Figure 17 *^{31}P NMR (284 MHz, H_2O/D_2O (9:1), zgpg30, -200-150 ppm) spectra to show the irradiation (254 nm) of 12 mM threo-**9** and 12 mM β-2',3'-cyclic-phosphate-erythro-**13** in H_2O/D_2O (9:1) at pH 7 with NaSH (0.11 mmol, 109 mM), MSM internal standard (18.1 mM, resonance at 3.09 ppm): a) before the irradiation; b) after 1 day of irradiation; c) after 2 days of irradiation and d) after 3 days of irradiation.*

Irradiation of β-*erythro*-**13**

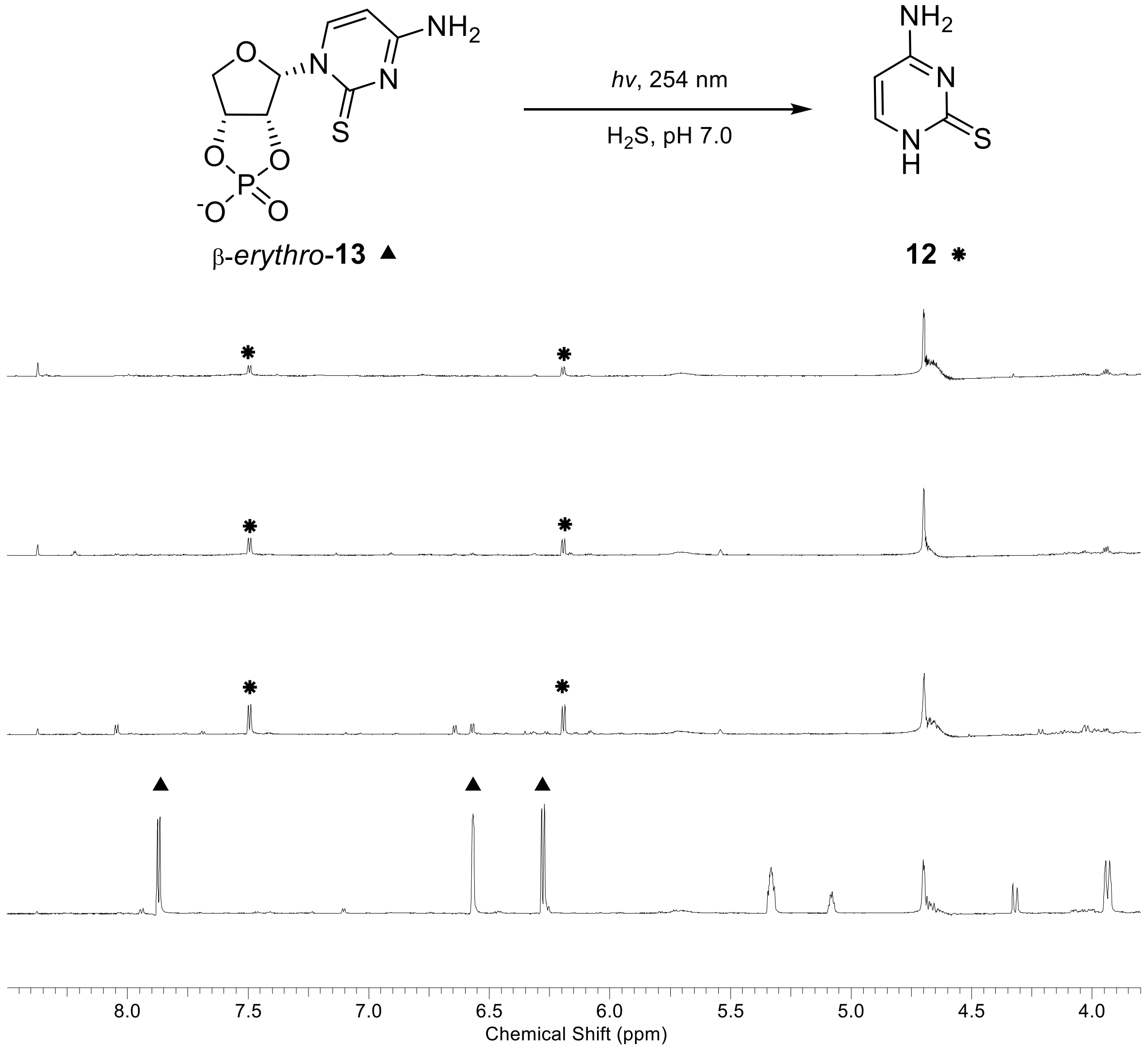

Supplementary Figure 18 *^{1}H NMR (700 MHz, H_2O/D_2O (9:1), Robust5_ver_03b, 3.8-8.5 ppm) spectra to show the irradiation at 254 nm of 7.1 mM β-2′,3′-cyclic-phosphate-erythro-**13** in H_2O/D_2O (9:1) at pH 7 with NaSH (0.09 mmol, 184 mM), MSM internal standard (18.1 mM, resonance at 3.09 ppm omitted): a) before the irradiation; b) after 1 day of irradiation (**12** (31%)); c) after 2 days of irradiation (**12** (18%)); d) after 3 days of irradiation (**12** (18%). (C6)–H, (C5)–H and (C1′)–H protons are labelled for β-2′,3′-cyclic-phosphate-erythro-**13** (▲) and (C6)–H and (C5)–H protons for 2-thiocytosine **12** (❋).*

Phosphorylation and subsequent irradiation of β-*threo*-**9** and treatment by phosphatase

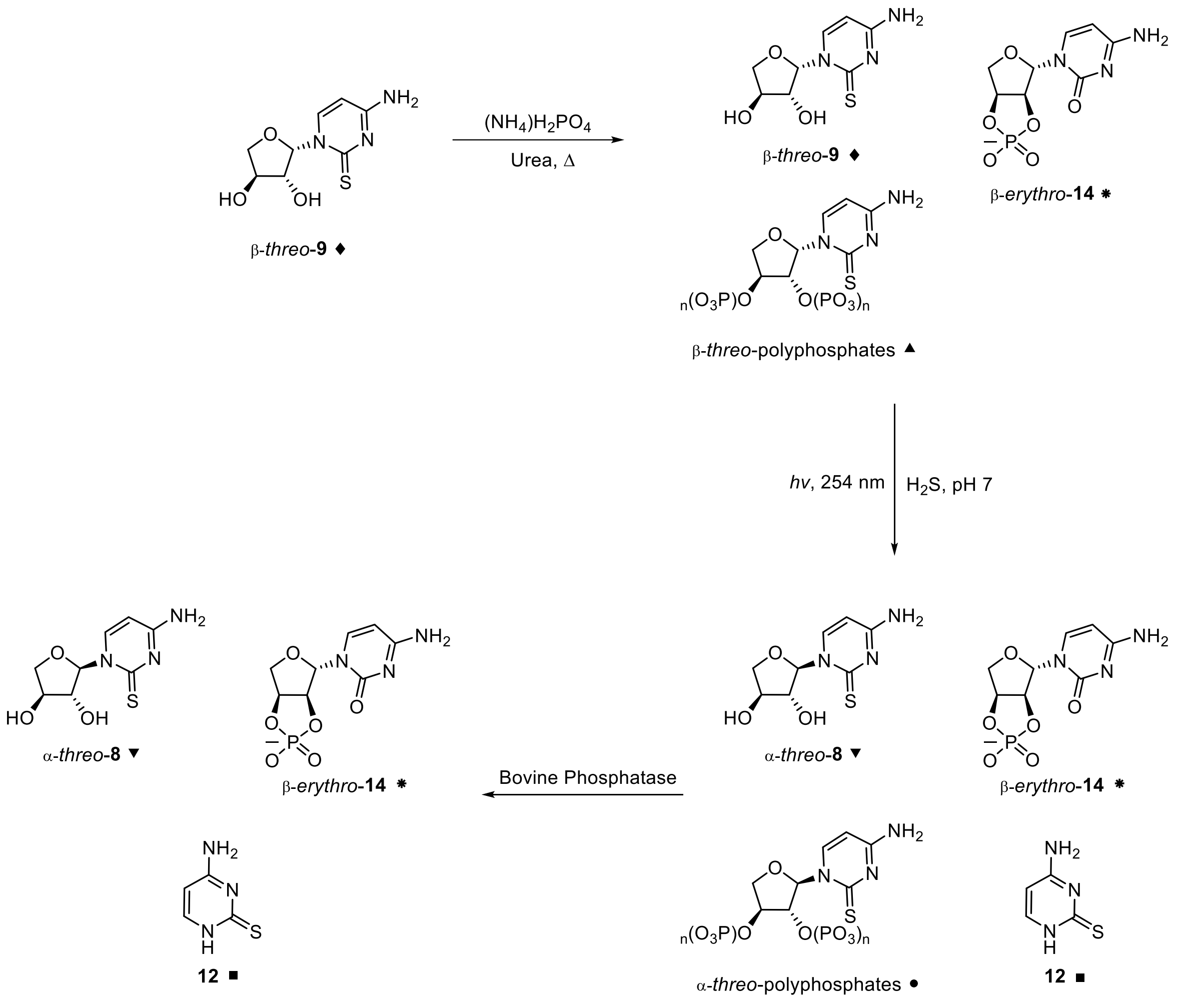

Supplementary Figure 19 *to show the phosphorylation and subsequent irradiation of β-threo-**9** and treatment by phosphatase*

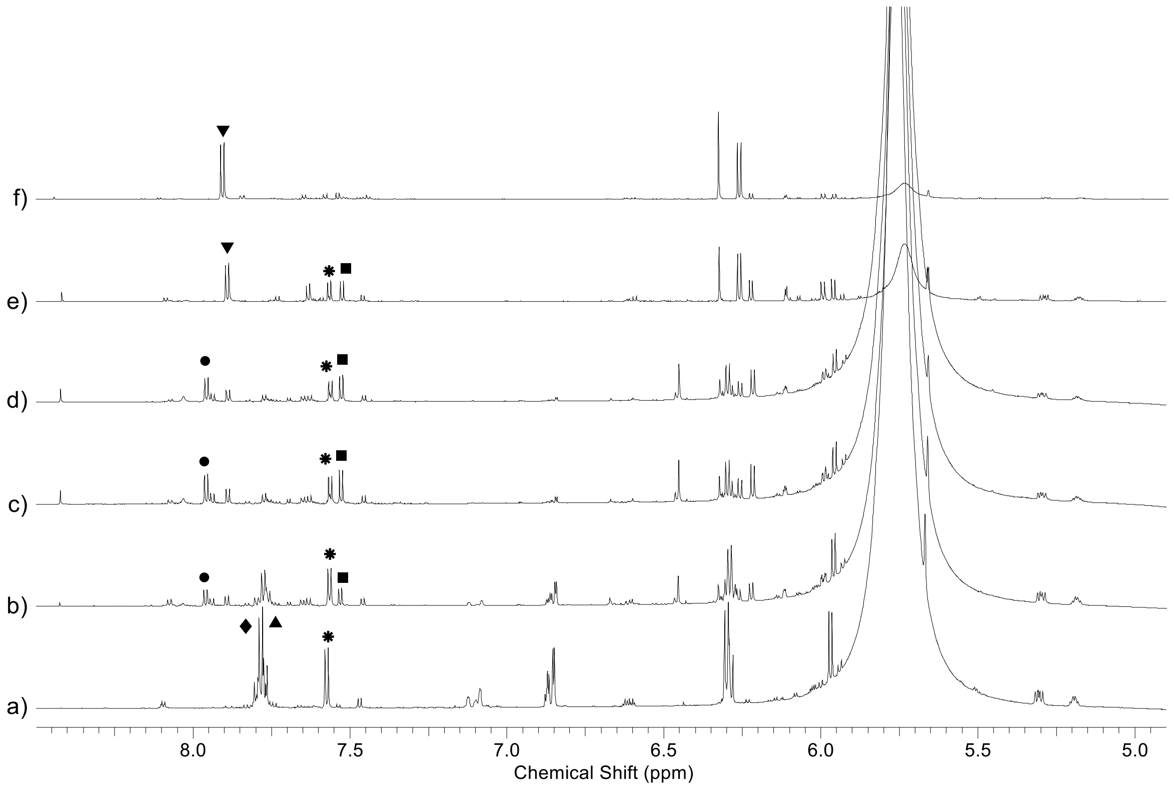

Supplementary Figure 20 *^{1}H NMR (700 MHz, H_2O/D_2O (9:1), Robust5_ver_03b, 4.9-8.5 ppm) spectra to show: a) phosphorylation of β-threo-**9** by Phosphorylation Method 2; b) irradiation (254 nm) of a) with NaSH (0.1 mmol, 199 mM) after 1 day; c) after 2 days of irradiation; d) after 3 days of irradiation; e) spectra d) after undergoing Phosphatase Method 1; f) spectra e) spiked with authentic standard of α-threo-**8**. (C6)–H protons are labelled: β-threo-**9** (◆), β-threo-polyphosphates (▲), β-2′,3′-cyclic-phosphate-erythro-**14** (✱), α-threo-polyphosphates (●), α-threo-**8** (▼), 2-thiocytosine **12** (■).*

Synthesised Compounds

Tetrose-aminooxazolines

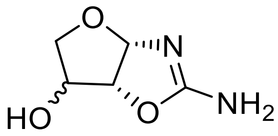

2-Aminooxazole **2AO** (756 mg, 9 mmol) and glycolaldehyde **5** (540 mg, 9 mmol) were dissolved in water (10 mL) and the solution adjusted to pH 7. The solution was stirred at room temperature for 16 h and then lyophilised to afford tetrose aminooxazoline **TAO**/**EAO** (1.02 g, 78%) as an orange crystalline solid.

^{1}H NMR (600 MHz, D_2O)	δ = 5.85 (d, *J* = 5.1 Hz, 1 H, (C1′)–H-*threo*), 5.72 (d, *J* = 5.2 Hz, 1 H, (C1′)–H-*erythro*), 4.85 (t, *J* = 5.2 Hz, 1 H, (C2′)–H-*erythro*), 4.80 (d, *J* = 5.1 Hz, 1 H, (C2′)–H-*threo*), 4.33 (d, *J* = 2.6 Hz, 1 H, (C3′)–H-*threo*), 4.28 (ddd, *J* = 5.1, 6.7, 10.0 Hz, 1 H, (C3′)–H-*erythro*), 3.91 (dd, *J* = 6.7, 9.5 Hz, 1 H, (C4′)–H-*erythro*), 3.80 (dd, *J* = 0.8, 11.0 Hz, 1 H, (C4′)–H-*threo*), 3.57 (dd, *J* = 2.6, 11.0 Hz, 1 H, (C4′′)–H-*threo*), 3.21 (t, *J* = 9.5 Hz, 1 H, (C4′′)–H-*erythro*).
^{13}C NMR (151 MHz, D_2O)	δ = 167.0 (C2-*erythro*), 165.8 (C2-*threo*), 98.8 (C1′-*erythro*), 98.3 (C1′-*threo*), 87.4 (C2′-*threo*), 82.0 (C2′-*erythro*), 74.2 (C3′-*threo*), 71.1 (C3′-*erythro*), 70.5 (C4′-*threo*), 66.1 (C4′-*erythro*).
HRMS	ESI-HRMS (pos. m/z): $[M+H]^+$ calculated for $C_5H_9N_2O_3^+$: 145.0613; found: 145.0615.
IR (solid, cm^{-1})	3328 (broad, weak, O−H), 1661 (strong, sharp C=N).
Melting Point (°C)	82-90[1]

[1] NB: Melting point recorded for diastereomeric mixture of products.

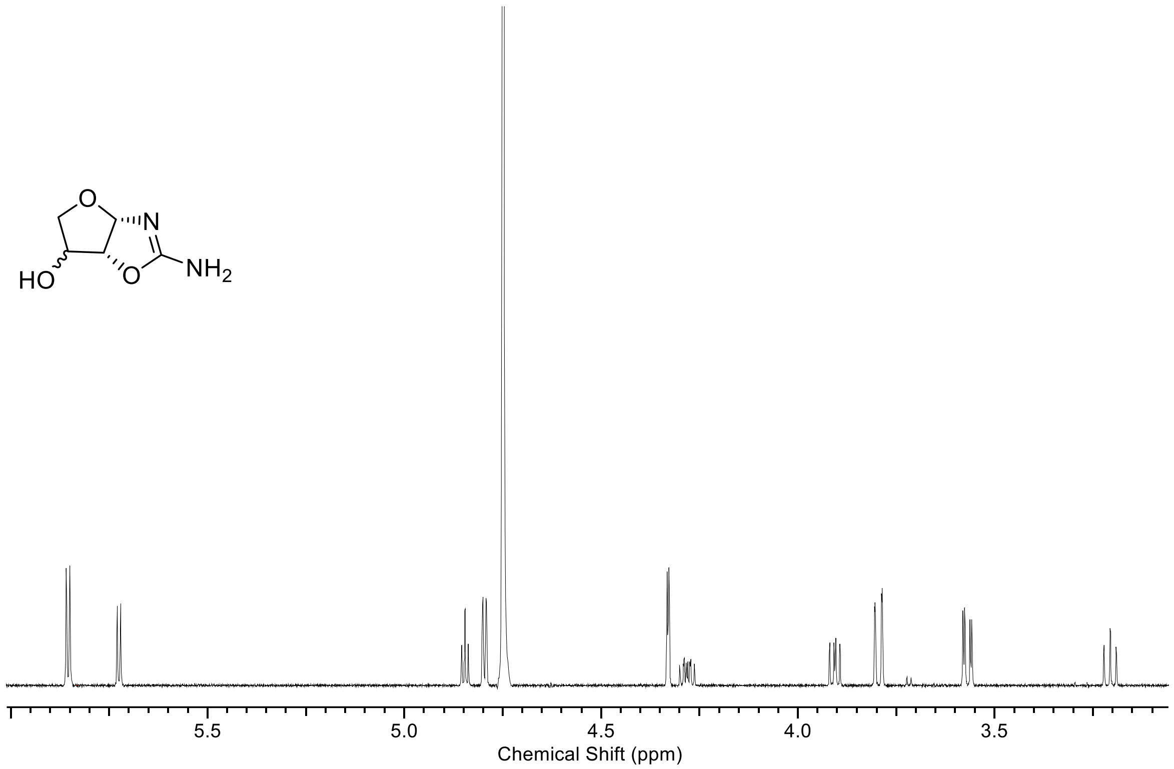

Supplementary Figure 21 *^{1}H NMR (600 MHz, D_2O, zg30, 3.0-6.0 ppm) spectrum to show a mixture of threo-aminooxazoline **TAO** and erythro-aminooxazoline **EAO.***

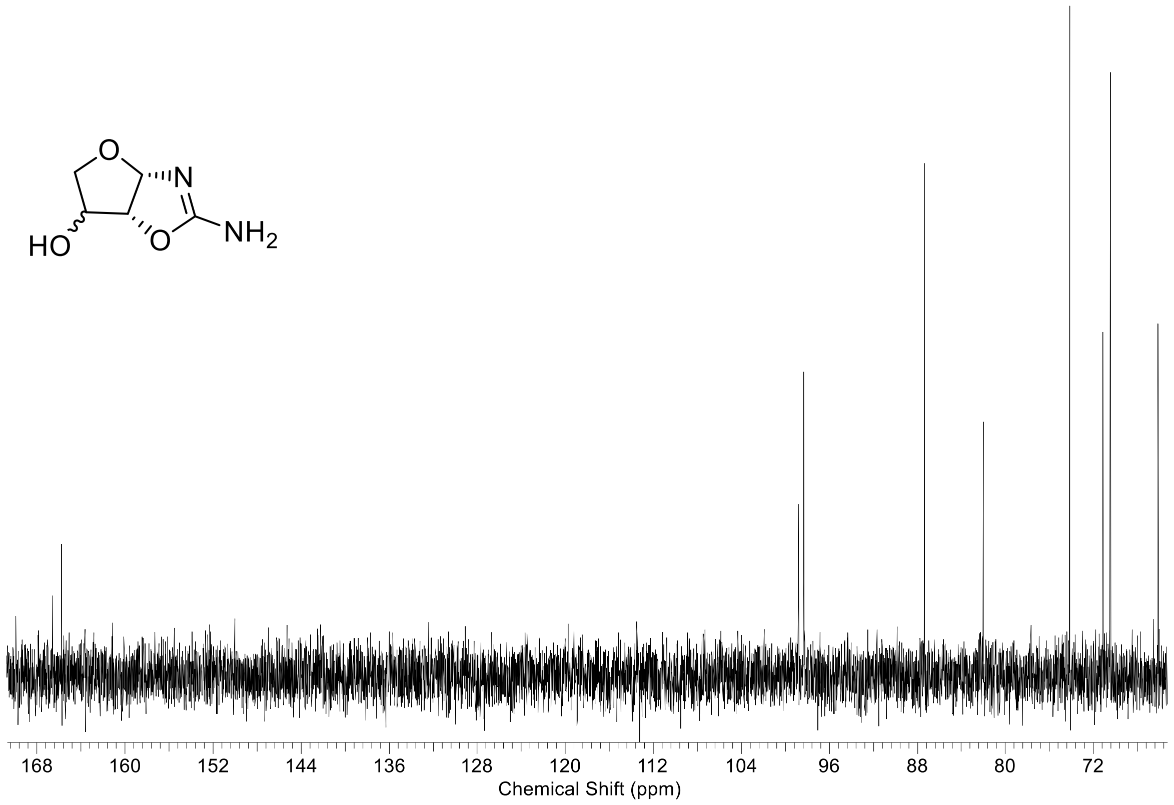

Supplementary Figure 22 *^{13}C NMR (151 MHz, D_2O, 65-170 ppm) spectrum to show a mixture of threo-aminooxazoline **TAO** and erythro-aminooxazoline **EAO**.*

Tetrofuranosyl-2,2′-anhydrocytdine (**6**/**7**)

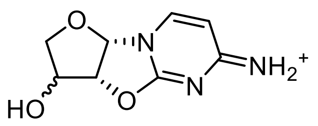

Threo-aminooxazoline **TAO** and *erythro*-aminooxazoline **EAO** (0.359 mg, 2.5 mmol) and sodium dihydrogen phosphate (0.357 mg, 3 mmol) were dissolved in water (6 mL) and the solution was adjusted to pH 6.5. Cyanoacetylene (1.276 g, 25 mmol) was added and the reaction was stirred for 18 h at room temperature. The resulting yellow solution was lyophilised to give a yellow powder (906 mg) that contained tetrofuranosyl-2,2′-anhydrocytdines **6** and **7**. The lyophilisate was dissolved in D_2O (0.5 mL) and analysed by NMR spectroscopy. Cyanovinyl phosphate, but no residual aminooxazoline (**TAO** or **EAO**) was observed. This material was carried forward to the next step without purification.

^{1}H NMR (600 MHz, D_2O)	δ = 8.03 (d, *J* = 7.3 Hz, 1 H, (C6)–H-*threo*), 8.01 (d, *J* = 7.3 Hz, 1 H, (C6)–H-*erythro*), 6.55-6.52 (m, 3 H, (C5)–H-*threo*, (C5)–H-*erythro*, (C1′)–H-*threo*), 6.43 (d, *J* = 5.2 Hz, 1 H, (C1′)–H-*erythro*), 5.47 (t, *J* = 5.2 Hz, 1 H, (C2′)–H-*erythro*), 5.43 (d, *J* = 5.2, 1 H, (C2′)–H-*threo*), 4.64 (d, *J* = 2.7 Hz, 1 H, (C3′)–H-*threo*), 4.59 (ddd, *J* = 5.2, 6.6, 9.5 Hz, 1 H, (C3′)–H-*erythro*), 4.18 (dd, *J* = 6.6, 9.5 Hz, 1 H, (C4′)–H-*erythro*), 4.10 (dd, *J* = 1.0, 10.2 Hz, 1 H, (C4′)–H-*threo*), 3.86 (dd, *J* = 2.7, 10.2 Hz, 1 H, (C4′′)–H-*threo*), 3.60 (t, *J* = 9.5 Hz, 1 H, (C4′′)–H-*erythro*).
^{13}C NMR (151 MHz, D_2O)	δ = 168.2 (C2-*threo*), 167.6 (C2-*erythro*), 160.1 (C4-*threo*), 160.1 (C4-*erythro*) 141.0 (C6-*threo*) 140.8 (C6-*erythro*), 103.9 (C5-*threo*), 103.8 (C5-*erythro*), 91.8 (C1′-*erythro*), 91.7 (C1′-*threo*), 88.7 (C2′-*threo*), 83.6 (C2′-*erythro*), 73.8 (C4′-*threo*), 72.1 (C3′-*threo*), 70.1 (C3′-*erythro*), 69.1 (C4′-*erythro*).
HRMS	ESI-HRMS (pos. m/z): $[M+H]^+$ calculated for $C_8H_{11}N_3O_3^+$: 196.0722; found: 196.0720.
IR (solid, cm^{-1})	3097 (broad, O−H), 1637 (sharp, C=N), 1499 (medium, C=C).
Melting Point (°C)	150-165 °C (decomp.)

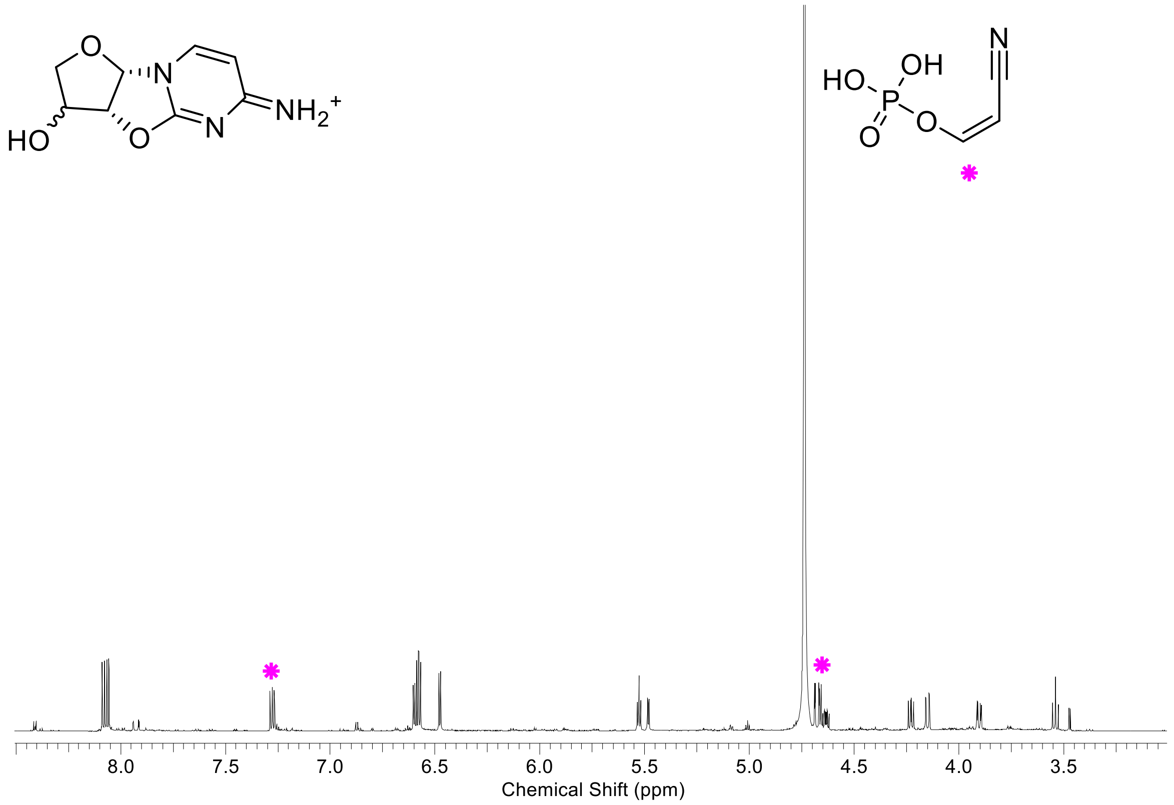

Supplementary Figure 23 *^{1}H NMR (600 MHz, D_2O, zg30, 3.0-8.5 ppm) spectrum to show a mixture of threo-anhydronucleoside **6** and erythro-anhydronucleoside **7** and cyanovinyl phosphate (*).*

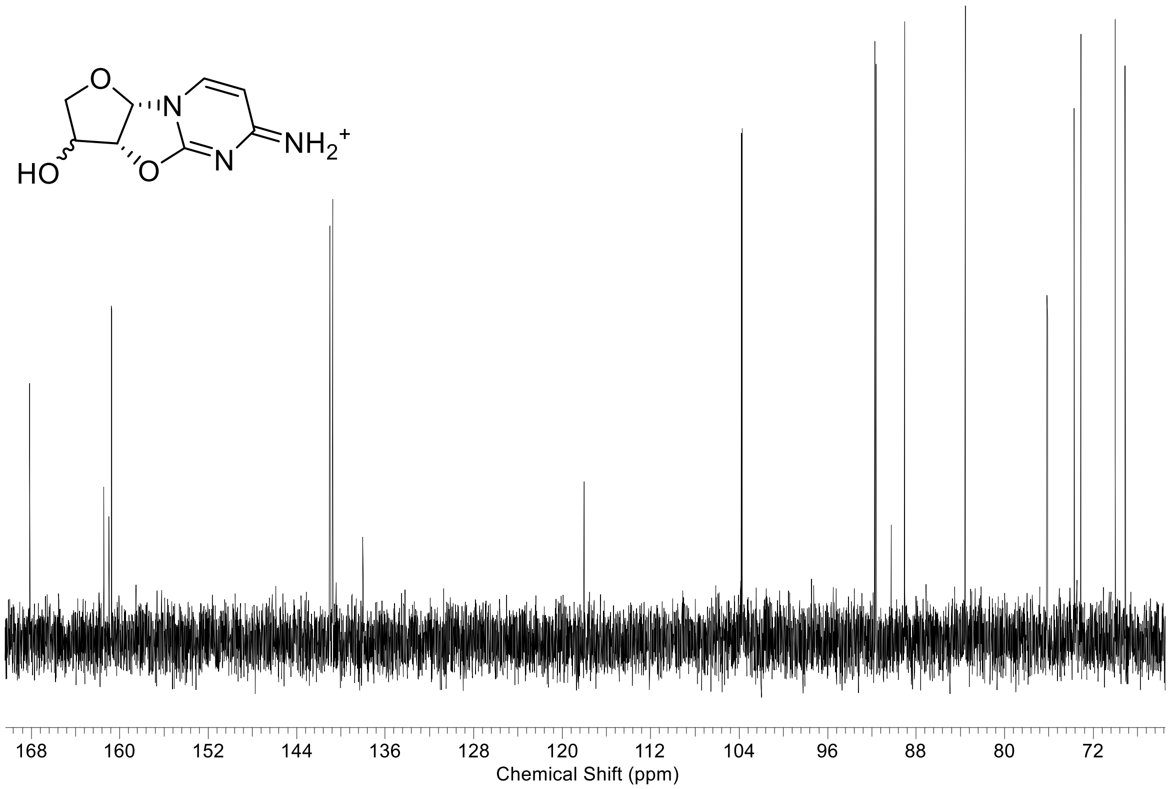

Supplementary Figure 24 *^{13}C NMR (176 MHz, D_2O, 65.5-170 ppm) spectrum to show a mixture of threo-anhydronucleoside* ***6*** *and erythro-anhydronucleoside* ***7****.*

Synthesis of 2-thiocytidine tetroses **9** and **10** in formamide

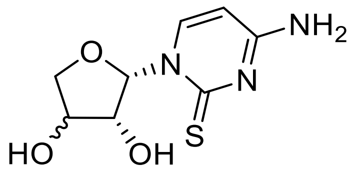

Tetrofuranosyl-2,2'-anhydrocytdines **6** and **7** (5.06 mg, 0.03 mmol) and sodium sulfide hydrate (59.3 mg, 0.63 mmol) were dissolved in formamide (1 mL) and stirred at room temperature overnight under an N_2 atmosphere. Water (5 ml) was added to the resulting solution and HCl under air ebullition until the solution reached pH 5, to degas and sparge H_2S from the solution. The sparged solution was adjusted to pH 7 and lyophilised. The resultant solid was dissolved in D_2O (1 mL) containing methylsulfonylmethane (10.57 mg, 0.11 mmol) as an internal standard and analysed by NMR spectroscopy. (4.63 mg, 78% qNMR against internal standard).

Two step synthesis of β-*tetro*-thiocytidines **9** and **10**

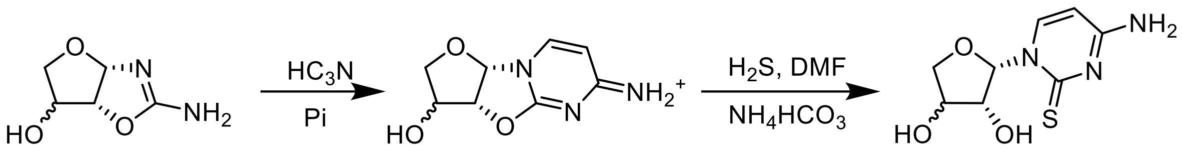

Threo-aminooxazoline **TAO** and *erythro*-aminooxazoline **EAO** (303 mg, 2.1 mmol) and sodium dihydrogen phosphate (252 mg, 2.1 mmol) were dissolved in water (5 mL) and the solution was adjusted to pH 6.5. Cyanoacetylene (9.35 mL, 8.41 mmol) was added and the reaction was stirred for 18 h at room temperature, the resulting yellow solution was lyophilised to give tetrofuranosyl-2,2'-anhydrocytdines **6** and **7** as a yellow powder. The crude mixture of **6** and **7** and ammonium bicarbonate (498 mg, 6.30 mmol) was dissolved in anhydrous DMF (20 mL) and NaSH (612 mg, 6.6 mmol) was added. The resulting blue solution was stirred for 18 h at room temperature. Water (40 ml) was added to the resulting solution and HCl under air ebullition until the solution reached pH 5, to degas and sparge H_2S from the solution. The sparged solution was adjusted to pH 7 and the solvent removed in vacuo. The crude product was purified by reverse phase FCC eluting with water/MeOH to give β-*threo*-**9** and β-*erythro*-**10** (178 mg, 37%).

Characterisation data for β-*threo*-**9**

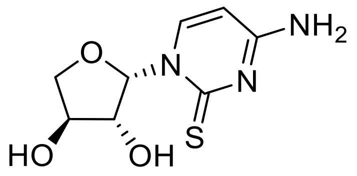

^{1}H NMR (700 MHz, D_2O)	*δ* = 7.78 (d, *J* = 7.6 Hz, 1H, (C6)–H), 6.88 (d, *J* = 3.6 Hz, 1H, (C1′)–H), 6.29 (d, *J* = 7.6 Hz, 1H, (C5)–H), 4.70 (d, *J* = 3.6 Hz, 1H, (C2′)–H), 4.39-4.36 (m, 2, (C3′)–H,(C4′)–H), 4.03 (d, *J* = 9.7 Hz, 1H, (C4′′)–H)
^{13}C NMR (176 MHz, D_2O)	*δ* = 179.0 (C2), 161.8 (C4), 143.7 (C6), 99.3 (C5), 93.0 (C1′), 76.2 (C3′), 74.7 (C4′), 74.2 (C2′)
HRMS	ESI-HRMS (pos. m/z): $[M+H]^+$ calculated for $C_8H_{12}N_3O_3S^+$: 230.0555; found 230.0599.
IR (solid, cm^{-1})	3171 (broad, weak, O-H), 1659 (strong, sharp, C-N), 1081 (strong, sharp, C-N)
Melting Point (°C)	168-172 (decomp.)

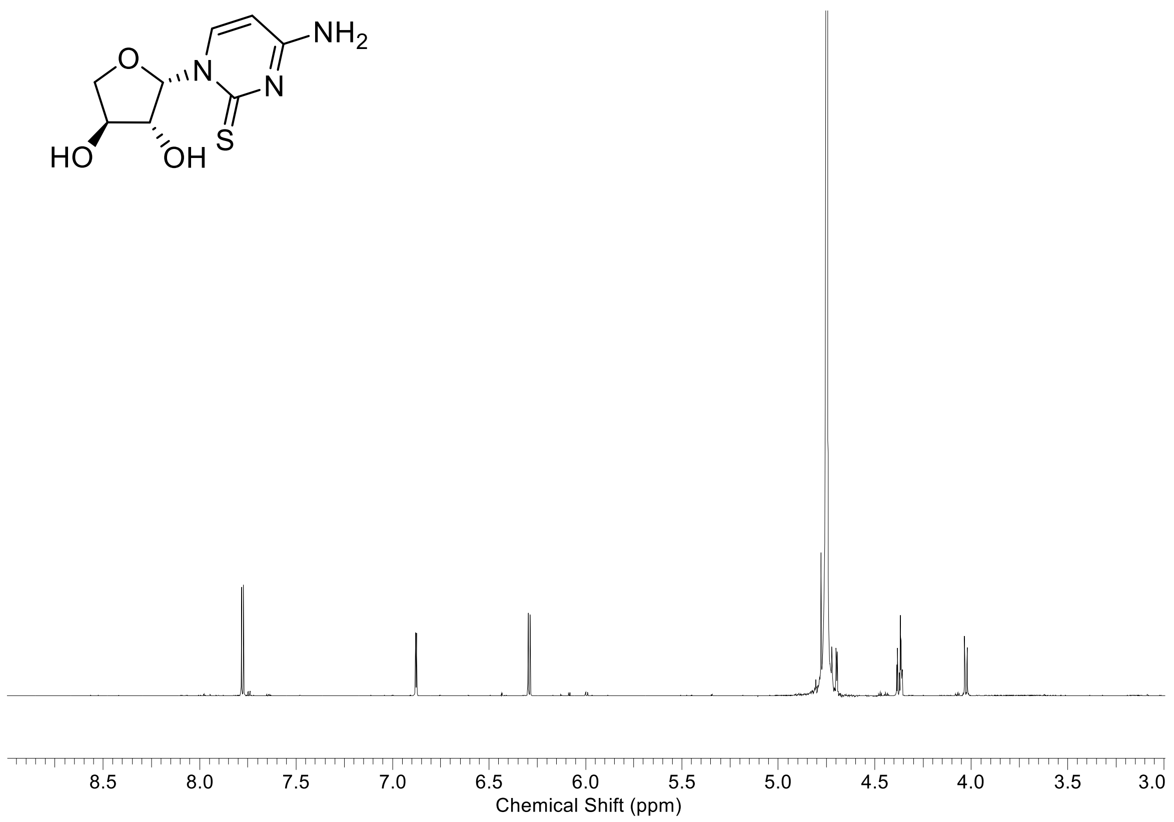

Supplementary Figure 25 *^1H NMR (700 MHz, D_2O, zg30, 3-9 ppm) spectrum to show β-threo-thiocytidine **9**.*

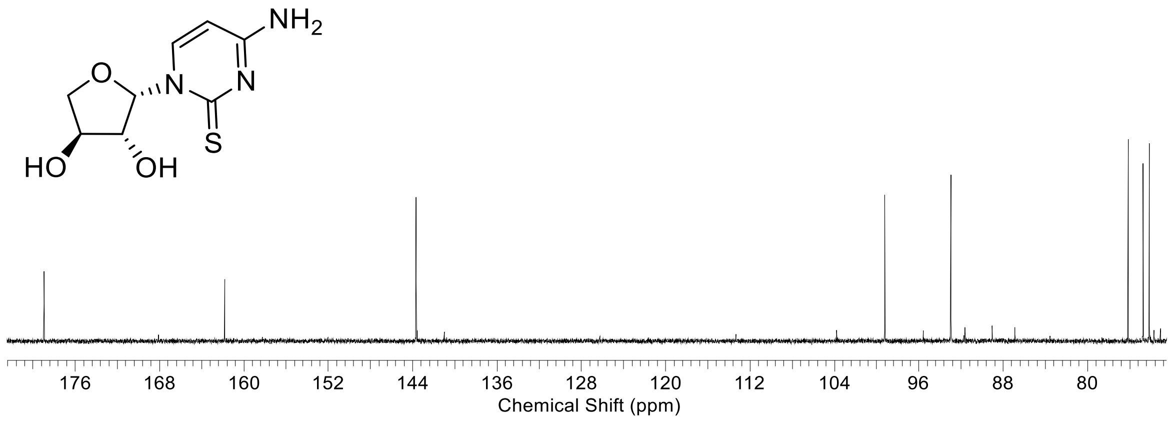

Supplementary Figure 26 *^{13}C NMR (176 MHz, D_2O, 72-182 ppm) spectrum to show β-threo-thiocytidine **9**.*

Characterisation data for β-*erythro*-**10**

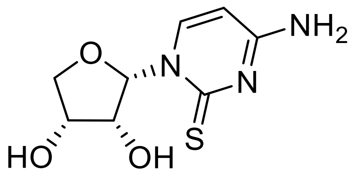

^{1}H NMR (700 MHz, D_2O)	*δ* = 7.88 (d, *J* = 7.6 Hz, 1H, (C6)–H), 6.86 (d, *J* = 5.0 Hz, 1H, (C1′)–H), 6.30 (d, *J* = 7.6 Hz, 1H, (C5)–H), 4.69 (t, *J* = 5.0 Hz, 1H, (C2′)–H), 4.48 (app. q, *J* = 5.5 Hz, 1H, (C3′)–H), 4.12 (dd, *J* = 5.5, 9.3 Hz, 1H, (C4′)–H), 4.06 (dd, *J* = 5.5, 9.3 Hz, 1H, (C4′′)–H)
^{13}C NMR (151 MHz, D_2O)	*δ* = 179.3 (C2), 161.8 (C4) 144.0 (C6), 99.4 (C5), 91.5 (C1′), 72.1 (C4′), 70.6 (C2′), 70.1 (C3′)
HRMS	ESI-HRMS (pos. m/z): $[M+H]^+$ calculated for $C_8H_{12}N_3O_3S^+$: 230.0555; found 230.0599.
IR (solid, cm^{-1})	3474-3197 (broad, weak, O-H), 1636 (strong, sharp, C-N), 1076 (strong, sharp, C-N)
Melting Point (°C)	210 (decomp.)

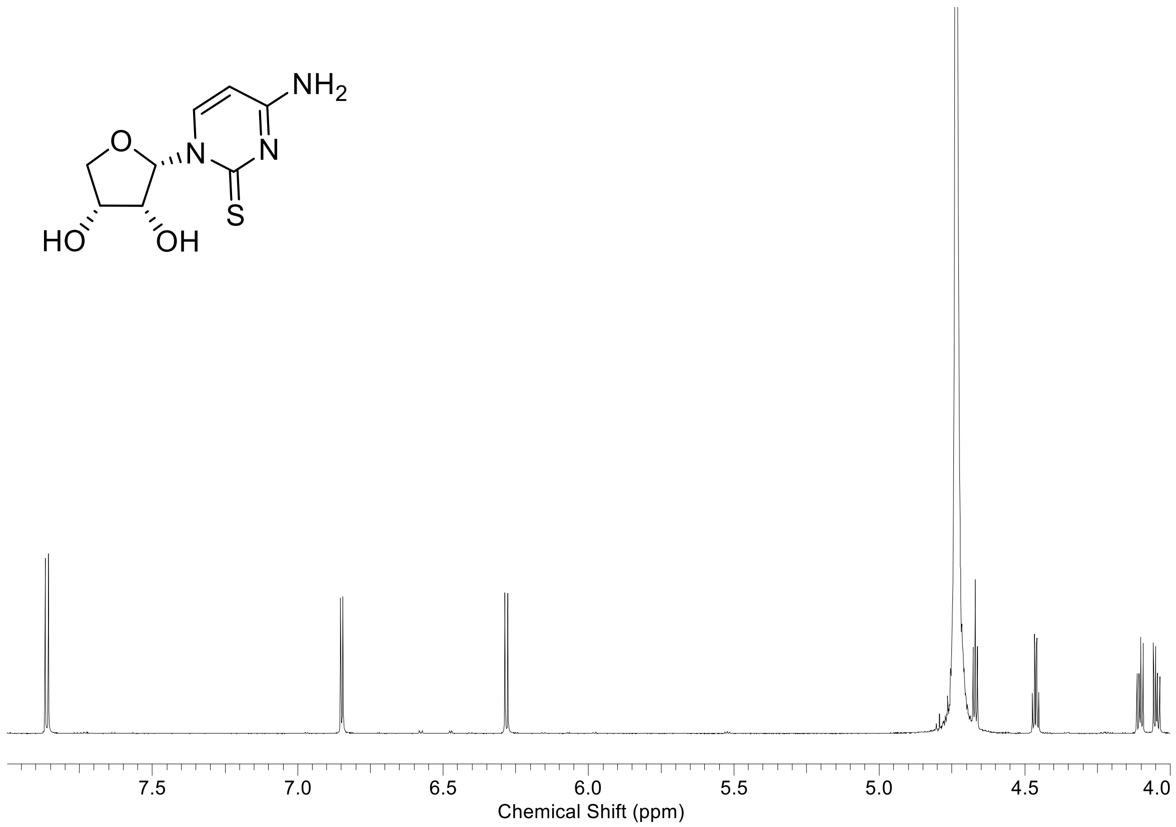

Supplementary Figure 27 *^1H NMR (700 MHz, D_2O, zg30, 4-8.0 ppm) spectrum to show β-erythro-thiocytidine* ***10****.*

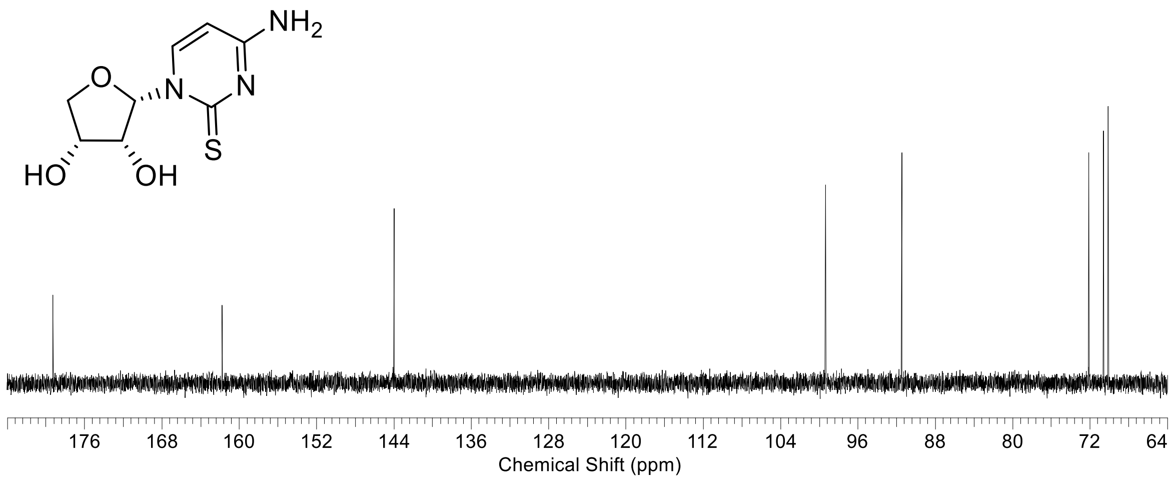

Supplementary Figure 28 *^{13}C NMR (176 MHz, D_2O, 64-184 ppm) spectrum to show β-erythro-thiocytidine* ***10****.*

Synthetic route for authentic standard of α-*threo*-**8**

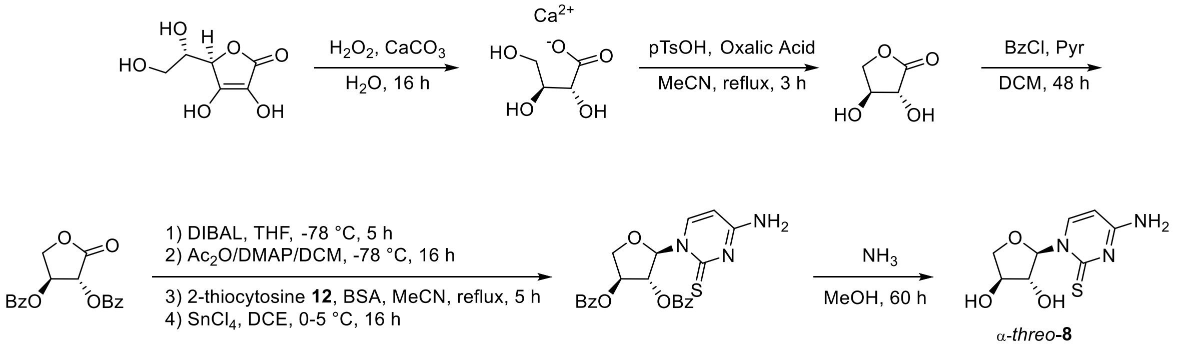

Supplementary Figure 29 *Synthetic route for the synthesis of α-threo-**8** as an authentic standard.*

Synthesis of calcium L-threonate

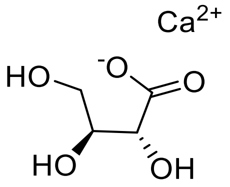

Procedure adapted from *Sau et al.*[2]

To a solution of L-ascorbic acid (25.1 g, 0.14 mol) in H_2O (200 mL) was slowly added of $CaCO_3$ (25.2 g, 0.25 mol) over 30 min, whilst the solution temperature was maintained between 0−5 °C. H_2O_2 (30%, 50 mL) was added dropwise to the resultant slurry over 1 h whilst stirring at 0−5 °C. The reaction mixture was allowed to warm to room temperature and stirred overnight. The mixture was filtered, and the solids washed with H_2O (2 × 20 mL). To the filtrate, activated carbon (5 g) was added and stirred at 50 °C, until peroxide was no longer detected by peroxide test strips (Quantofix® Peroxide 100). The hot suspension was filtered, and the solids washed with H_2O (2 × 10 mL). The volume of the filtrate was then reduced in vacuo to 20 mL. The resulting solution was precipitated in ice cold MeOH and filtered to give calcium L-threonate as a white powder (26.7 g, 60%).

^{1}H NMR (600 MHz, D_2O)	δ = 3.96 (d, J = 2.4 Hz, 1H, (C2′)–H), 3.89 (m, 1H, (C3′)–H), 3.52 (ABX, J = 5.2, 11.5 Hz, 1H, (C4′)–H) 3.62 (ABX, J = 7.7, 11.5 Hz, 1H, (C4′′)–H).
^{13}C NMR (151 MHz, D_2O)	δ = 179.5 (C1′), 73.4 (C3′), 73.1(C2′), 63.5 (C4′).
HRMS	ESI-HRMS (pos. m/z): $[M+H]^+$ calculated for $C_4H_9O_5^+$: 137.0405; found: 137.0444
IR (solid, cm^{-1})	3218.59 (broad, weak, O-H (acid)), 1581.59 (strong, C=O)
Melting Point (°C)	> 380

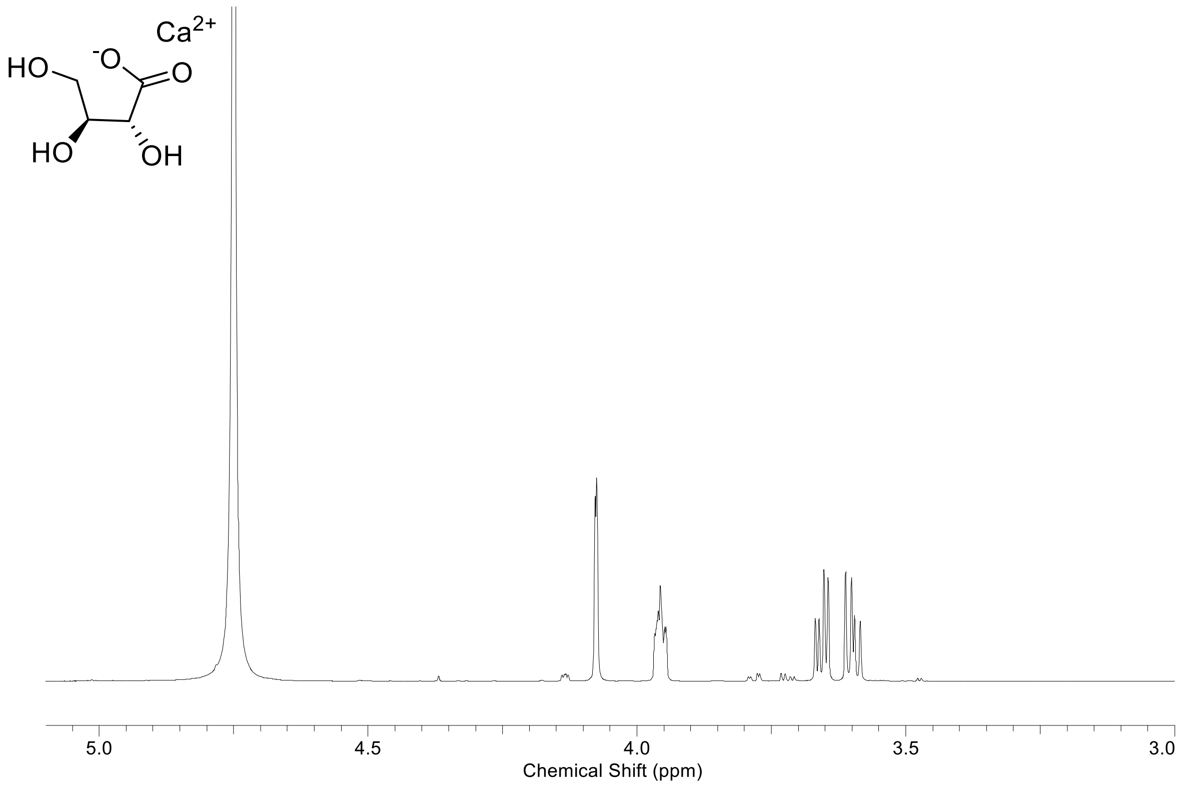

Supplementary Figure 30 *^{1}H NMR (700 MHz, D_2O, zg30, 3.0-5.1 ppm) spectrum to show calcium L-threonate.*

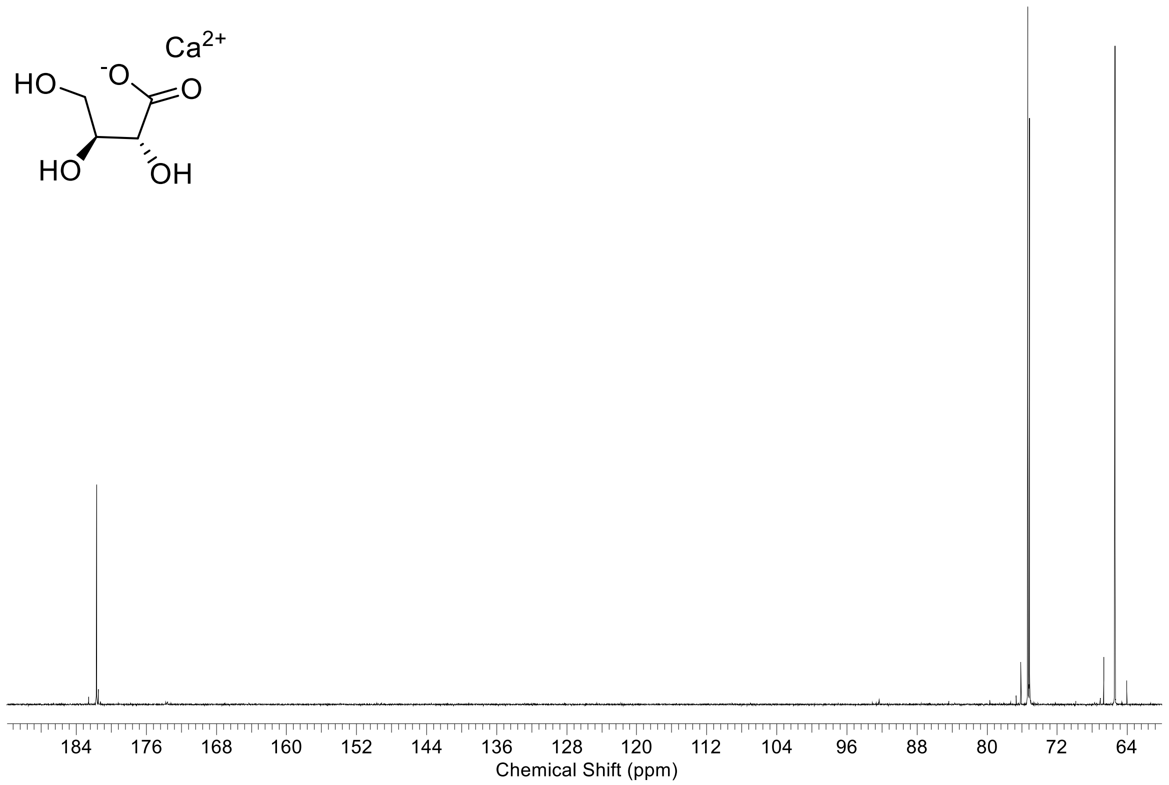

Supplementary Figure 31 *^{13}C NMR (176 MHz, D_2O, 60-192 ppm) spectrum to show calcium L-threonate.*

Synthesis of L-threonolactone

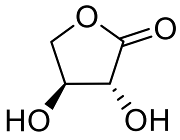

Procedure adapted from *Sau et al.*[2]

To a suspension of calcium L-threonate (25 g, 0.081 mol) in dry acetonitrile (125 mL) were added anhydrous oxalic acid (7.26 g, 0.081 mol) and *para*-toluenesulfonic acid monohydrate (0.258 g). The heterogeneous mixture was stirred at reflux for 3 h. The hot mixture was allowed to cool to room temperature and filtered. The solids were washed with 20 mL of acetonitrile, and the combined filtrate was evaporated under reduced pressure to produce a colourless syrup. The residue was dissolved in EtOAc (25 mL) and evaporated to dryness to give L-threonolactone as a white solid (12 g, 94%) which was used without further purification.

^{1}H NMR (600 MHz, MeOD)	δ = 4.41 (dd, J = 6.8, 8.9 Hz, 1H, (C4′)–H), 4.29 (q, J = 7.2, 1H, (C3′)–H), 4.19 (d, J = 7.2 Hz, 1H, (C2′)–H), 3.94 (dd, J = 7.2, 8.9 Hz, 1H, (C4′′)–H)
^{13}C NMR (100 MHz, MeOD)	δ = 177.4 (C1), 74.8 (C3), 74.1 (C2), 71.2 (C4).
HRMS	ESI-HRMS (pos. m/z): $[M+H]^+$ calculated for $C_4H_7O_4^+$: 119.0339; found 119.0687.
IR (solid, cm^{-1})	3344 (strong, broad, O-H), 1732 (strong, C=O)
Melting Point (°C)	66-70 (61-76[3])

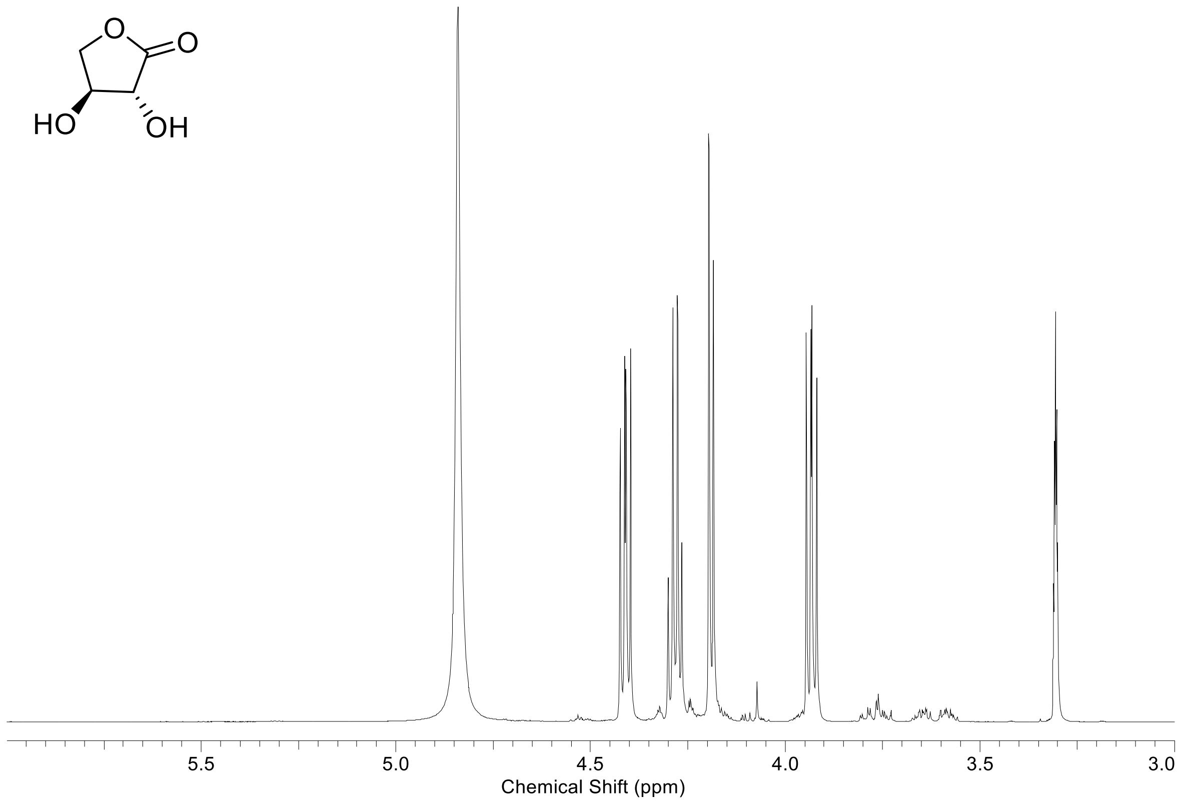

Supplementary Figure 32 *^{1}H NMR (600 MHz, MeOD, zg30, 3.0-5.1 ppm) spectrum to show L-threonolactone.*

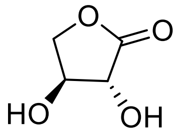

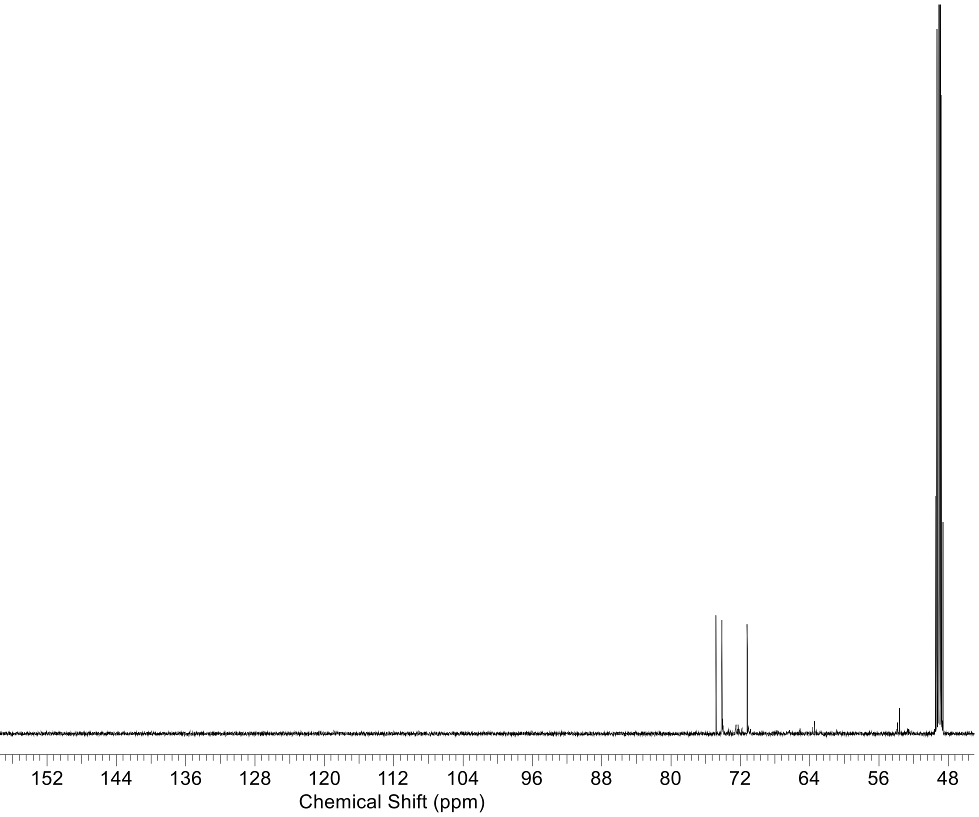

Supplementary Figure 33 *^{13}C NMR (151 MHz, MeOD, 45-180 ppm) spectrum to show L-threonolactone.*

Synthesis of 2′,3′-di-*O*-benzoyl-threonolactone

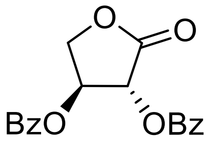

Procedure adapted from *Schöning et al.*[4]

L-Threonolactone (4.17 g, 35 mmol) and 4-dimethylaminopyridine (DMAP) (30 mg, 0.25 mmol) were dissolved in anhydrous pyridine (30 mL) and anhydrous dichloromethane (DCM) (10 mL) at 0 °C. Benzoyl chloride (10 mL, 86 mmol) was added dropwise under an N_2 atmosphere over 30 min. The homogenous mixture was allowed to warm to room temperature and then stirred for 2 d. The heterogenous mixture was then filtered and the filtrate concentrated in vacuo. The residue was diluted with DCM (25 mL) and washed with 1 M HCl (3 x 25 mL), water (25 mL), $NaHCO_3$ sat. (25 mL), and brine (25 mL). The organics were then dried over $MgSO_4$, filtered and concentrated in vacuo. The crude product was purified by normal phase FCC eluting with hexane/EtOAc (80:20) to give 2′,3′-di-*O*-benzoyl-threonolactone (3.01 g, 26 %).

^{1}H NMR (700 MHz, $CDCl_3$)	*δ* = 8.10 (d, *J* = 7.3 Hz, 2H, (C2′)-Ar-H), 8.05 (d, *J* = 7.3 Hz, 2H, (C3′)-Ar-H), 7.62 (t, *J* = 7.5 Hz, 2H, Ar-H), 7.48 (td, *J* = 1.6, 8.1 Hz, 4H, Ar-H), 5.81 (m, 2H, (C2′)–H, (C3′)–H) 5.02, (dd, *J* = 6.7, 10.2 Hz, 1H, (C4′)–H), 4.37 (dd, *J* = 5.2, 10.2 Hz, 1H, (C4′′)–H)
^{13}C NMR (176 MHz, $CDCl_3$)	*δ* = 169.7 (C1′), 165.8 (CO<u>C</u>OAr), 165.5 (CO<u>C</u>OAr), 134.2 (Ar-*para*), 134.2 (Ar-*para*), 130.4 (2C, Ar-*ortho*), 130.1 (2C, Ar-*ortho*), 128.8 (2C, Ar-*meta*), 128.8 (2C, Ar-*meta*), 128.4 (Ar-*ipso*), 128.2 (Ar-*ipso*), 73.4, 72.3, 69.6 (C4′)
HRMS	ESI-HRMS (pos. m/z): $[M+H]^+$ calculated for $C_{18}H_{15}O_6^+$: 327.0863; found 327.0865.
IR (solid, cm^{-1})	1678 (strong, C=O), 1287 (strong), 703 (strong)
Melting Point (°C)	114 – 119 (114[5])

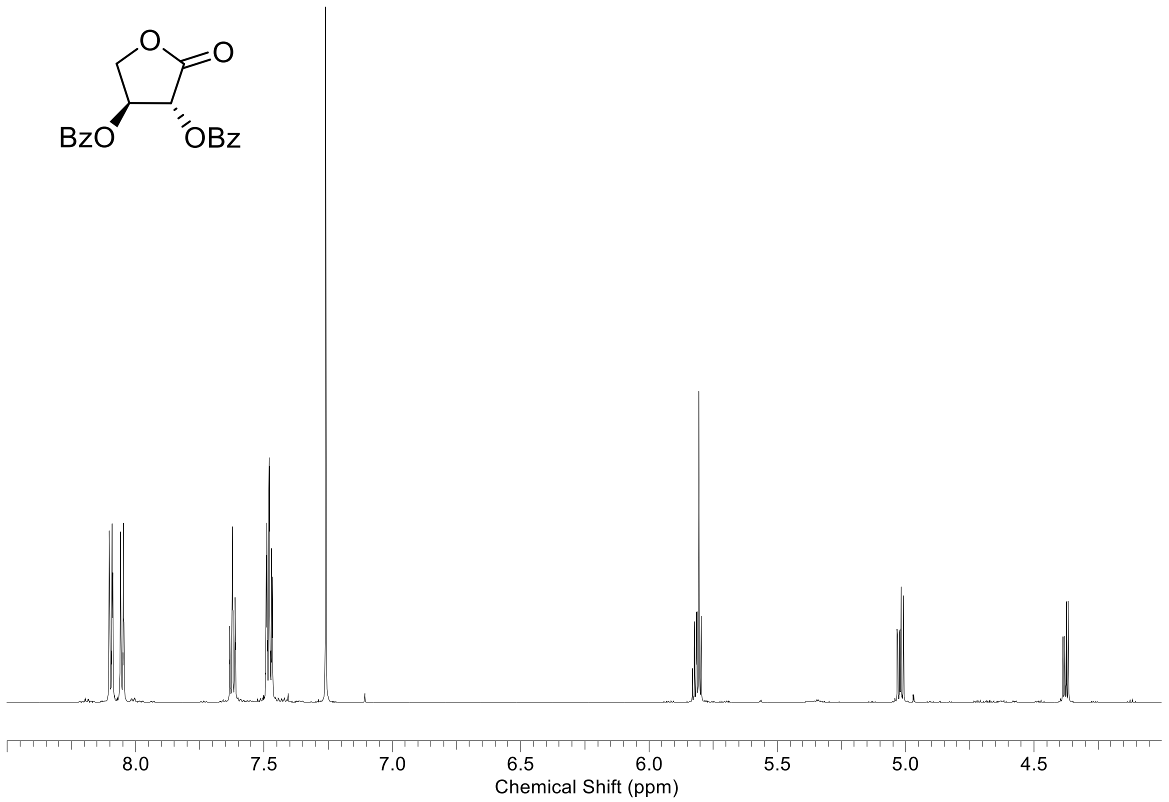

Supplementary Figure 34 *^{1}H NMR (700 MHz, $CDCl_3$, zg30, 4.0-8.5 ppm) spectrum to show 2',3'-di-O-benzoyl-threonolactone.*

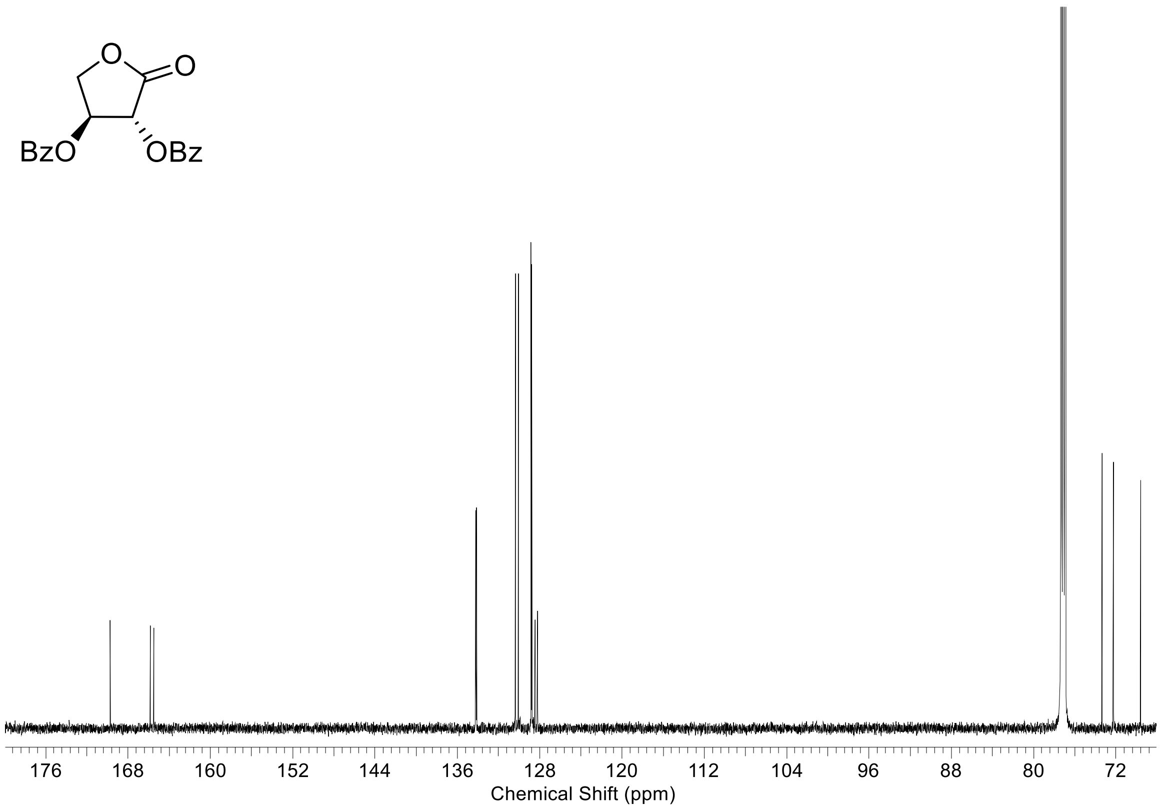

Supplementary Figure 35 *^{13}C NMR (176 MHz, $CDCl_3$, 68-180 ppm) spectrum to show 2',3'-di-O-benzoyl-threonolactone.*

Silylation of 2-thiocytosine **12**

2-Thiocytosine **12** (402 mg, 3.16 mmol) was added to a flame dried flask under N_2 atmosphere and dissolved in Bis(trimethylsilyl)acetamide (BSA) (1.58 mL, 6.46 mmol) and anhydrous acetonitrile (22.5 mL). The heterogenous mixture was refluxed for 2 h or until homogenous. The mixture was evaporated to an orange residue, redissolved in anhydrous dichloroethane (DCE) (5 mL) under N_2 atmosphere and used immediately without purification.

Synthesis of 2′,3′-di-*O*-benzoyl-2-thio-threocytidine

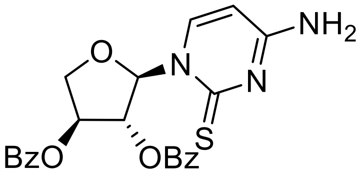

2′,3′-Di-*O*-benzoyl-threonolactone (700 mg, 2.15 mmol) was added under a N_2 atmosphere and dissolved in anhydrous THF (10 mL). The mixture was cooled to -78 °C and DIBAL-H (1.2 M in toluene, 2.5 mL, 3 mmol) added dropwise over 30 minutes. The reaction was maintained at -78 °C for 10 h and monitored by TLC. When the starting material was consumed Ac_2O/DCM/DMAP (1.2 ml, 12.7 mmol/1.5 ml/ 57 mg, 0.47 mmol) was added at -78 °C, over 20 mins, under a N_2 atmosphere. The reaction was allowed to warm to room temperature over 16 h. Hexane (30 mL) was added and the mixture was added to rapidly stirring 1 M HCl (50 mL) and stirred vigorously for 1 h. The aqueous layer was separated and washed with EtOAc (3 x 50 mL). The combined organic layers were separated and washed with water (50 mL), $NaHCO_3$ sat. (50 mL) and brine (50 mL), dried over $MgSO_4$ and evaporated to give a crude yellow oil. To this, DCM (15 mL) was added and co-evaporated 3 times before anhydrous dichloroethane (DCE) (3 mL) was added under N_2 atmosphere. To this mixture, freshly prepared, silylated 2-thiocytosine **12** (402 mg, 3.16 mmol, dissolved in 5 mL anhydrous DCE) was added and the mixture cooled to 0 °C before $SnCl_4$ (1.2 mL, 10.3 mmol) was added over 20 minutes. The now orange homogenous mixture was allowed to come to room temperature over 16 h and monitored by TLC. When the reaction was judged complete the reaction was quenched with $NaHCO_3$ sat. (15 mL) and stirred vigorously for 2 h. The mixture was filtered through Celite® 545, washed with DCM (30 mL) and $NaHCO_3$ sat. (15 mL) and the organics separated. The aqueous phase was washed with DCM (3 x 10 mL). The combined organics were washed with brine (30 mL) and dried over $MgSO_4$ before the solvent was removed in vacuo. The crude product was purified with FCC eluting with a mixture of DCM/MeOH to give 2′,3′-di-*O*-benzoyl-2-thiothreocytidine (581.2 mg, 62%) as a brown solid.

^{1}H NMR (700 MHz, $CDCl_3$)	δ = 8.14 (d, *J* = 7.1 Hz, 2H, (C2′)-Ar-H-*ortho*), 7.85 (d, *J* = 7.1 Hz, 2H, (C3′)-Ar-H-*ortho*), 7.81 (d, *J* = 7.5 Hz, 1H, (C6)–H), 7.63 (dd, *J* = 7.4, 7.5 Hz, 1H, (C2′)-Ar-H-*para*), 7.60 (dd, *J* = 7.4, 7.5 Hz, 1H, (C3′)-Ar-H-*para*), 7.50 (dd, *J* =7.6, 7.9 Hz, 2H, (C2′)-Ar-H-*meta*), 7.43 (dd, *J* =7.6, 7.9 Hz, 2H, (C3′)-Ar-H-*meta*), 6.91 (s, 1H, (C1′)–H), 6.03 (s, 1H, (C2′)–H), 5.95 (d, *J* = 7.5 Hz, 1H, (C5)–H), 5.51 (d, *J* = 3.3 Hz, 1H, (C3′)–H), 4.62 (ABX, *J* = 3.3, 11.4 Hz, 1H, (C4′)–H,) 4.59 (ABX, *J* = 11.4 Hz, 1H, (C4′′)–H)
^{13}C NMR (176 MHz, $CDCl_3$)	δ = 180.5 (C2), 165.0 (C3′-OC̲OAr), 164.9 (C2′-OC̲OAr), 160.2 (C4), 141.6 (C6), 134.0 (C3′-Ar-*para*), 133.8 (C2′-Ar-*para*), 130.2 (2C, C2′-Ar-*ortho*), 130.0. (2C, C3′-Ar-*ortho*), 129.1 (C2′-Ar-*ipso*), 128.7 (2C, C3′-Ar-*meta*), 128.7 (2C, C2′-Ar-*meta*), 128.6 (C3′-Ar-*ipso*), 97.2 (C5), 94.8 (C1′) 80.3 (C2′), 75.9 (C3′), 75.6 (C4′).
HRMS	ESI-HRMS (pos. m/z): $[M+H]^+$ calculated for $C_{22}H_{20}N_3O_5S^+$: 438.1124; found 438.1153.
IR (solid, cm^{-1})	1720 (strong, C=O), 1644 (strong, C=C), 1095 (medium, broad C-N)
Melting Point (°C)	185-190 (decomp.)

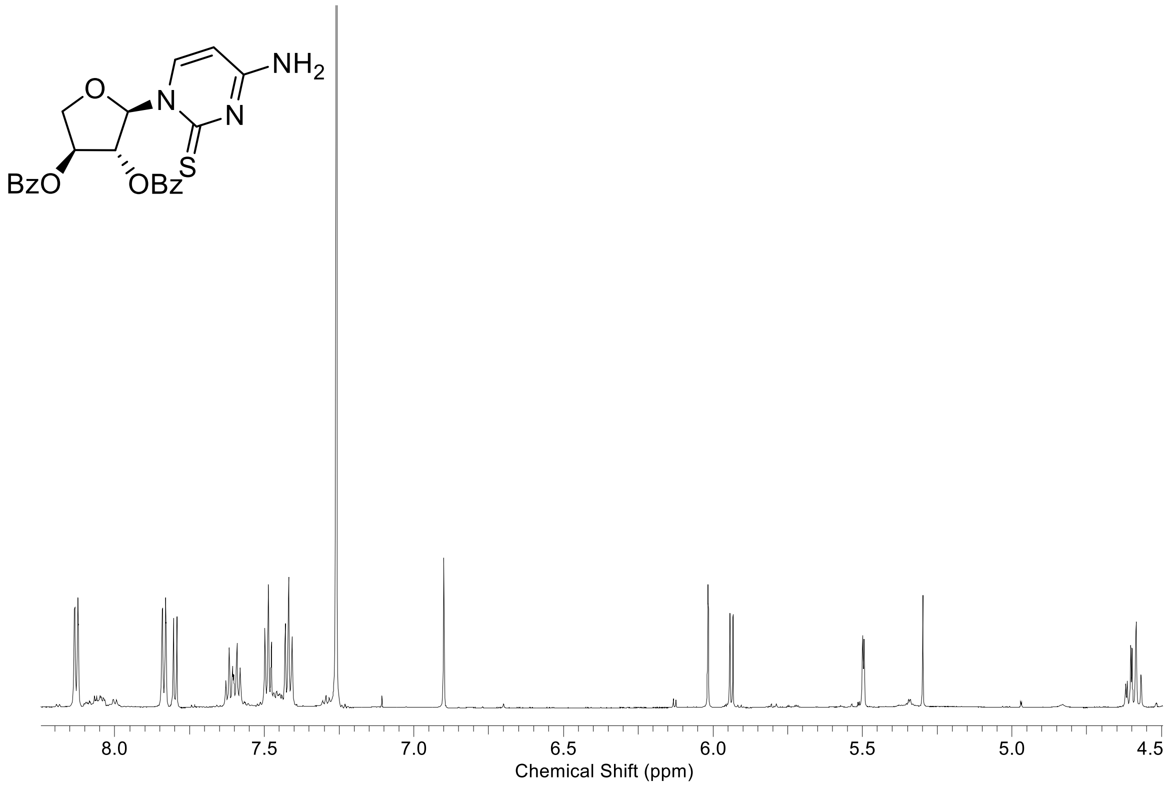

Supplementary Figure 36 *^{1}H NMR (600 MHz,$CDCl_3$, zg30, 4.5-8.25 ppm) spectrum to show 2',3'-di-O-benzoyl-2-thio-threocytidine.*

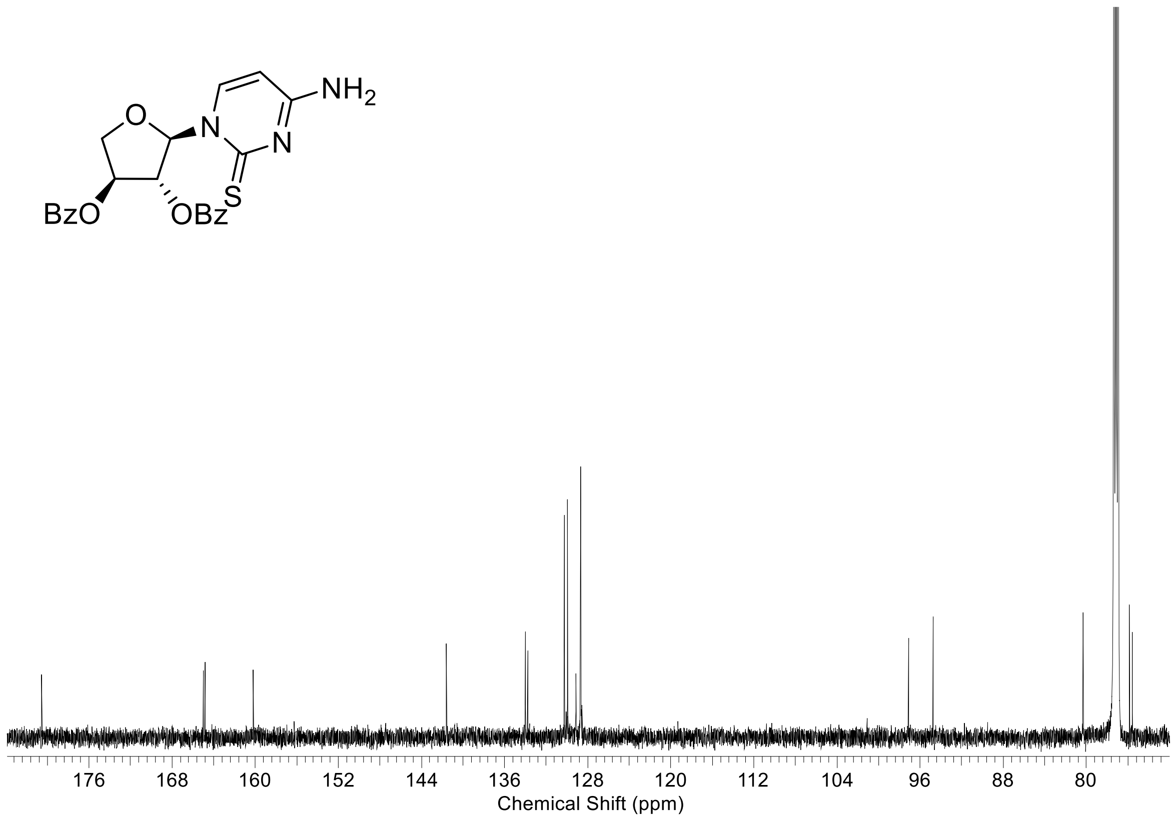

Supplementary Figure 37 *^{13}C NMR (176 MHz, $CDCl_3$, 72-183 ppm) spectrum to show 2',3'-di-O-benzoyl-2-thio-threocytidine.*

Synthesis of authentic standard of α-*threo*-thiocytidine **8**

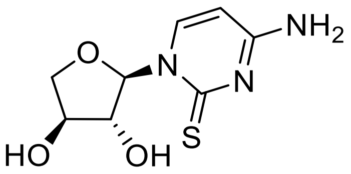

2′,3′-Di-*O*-benzoyl-2-thio-threocytidine (200 mg, 0.46 mmol) was dissolved in 7 M NH_3/MeOH (3.15 mL) and MeOH (12.6 mL) and the heterogenous mixture stirred at room temperature for 2.5 days. The solvent was evaporated and the residue redissolved in D_2O (7 mL) and washed DCM (2 x 7 mL). The aqueous layer was separated and lyophilised giving α-*threo*-thiocytidine **8** (98.2 mg, 94%) as a white powder. A sample was removed for NMR analysis.

^{1}H NMR (400 MHz, D_2O)	*δ* = 7.89 (d, J = 7.6 Hz, 1H, (C5)–H), 6.32 (s, 1H, (C1′)–H), 6.24 (d, J = 7.6 Hz, 1H, (C6)–H), 4.45 (app. s, 1H, (C2′)–H), 4.42 (app. td, J = 2.7 , 10.3 Hz, 2H, (C4′)–H, (C4′′)–H), 4.28 (app. dt, J = 1.1, 2.7 Hz, 1H, (C3′)–H)
^{13}C NMR (100.61 MHz, D_2O)	*δ* = 178.8 (C2), 162.0 (C4), 143.0 (C5), 99.1 (C1′), 97.4 (C6), 80.9 (C2′), 77.7 (C4′), 75.1 (C3′)
HRMS	ESI-HRMS (pos. m/z): $[M+H]^+$ calculated for $C_8H_{12}N_3O_3S^+$: 230.0592; found 230.0599.
IR (solid, cm^{-1})	3396 (broad), 3103 (broad), 1634 (strong), 1476 (medium, C=C)

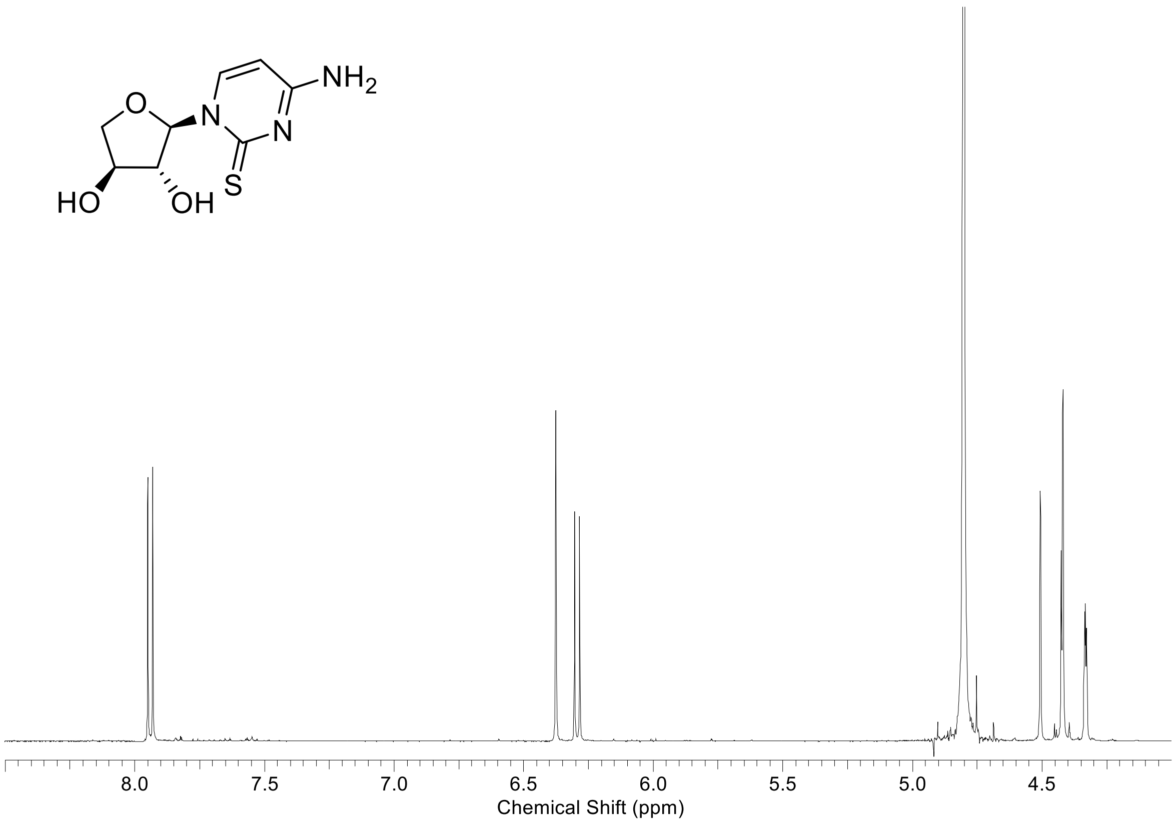

Supplementary Figure 38 *^{1}H NMR (400 MHz, D_2O, zg30, 4.0-8.5 ppm) spectrum to show α-threo-thiocytidine* ***8****.*

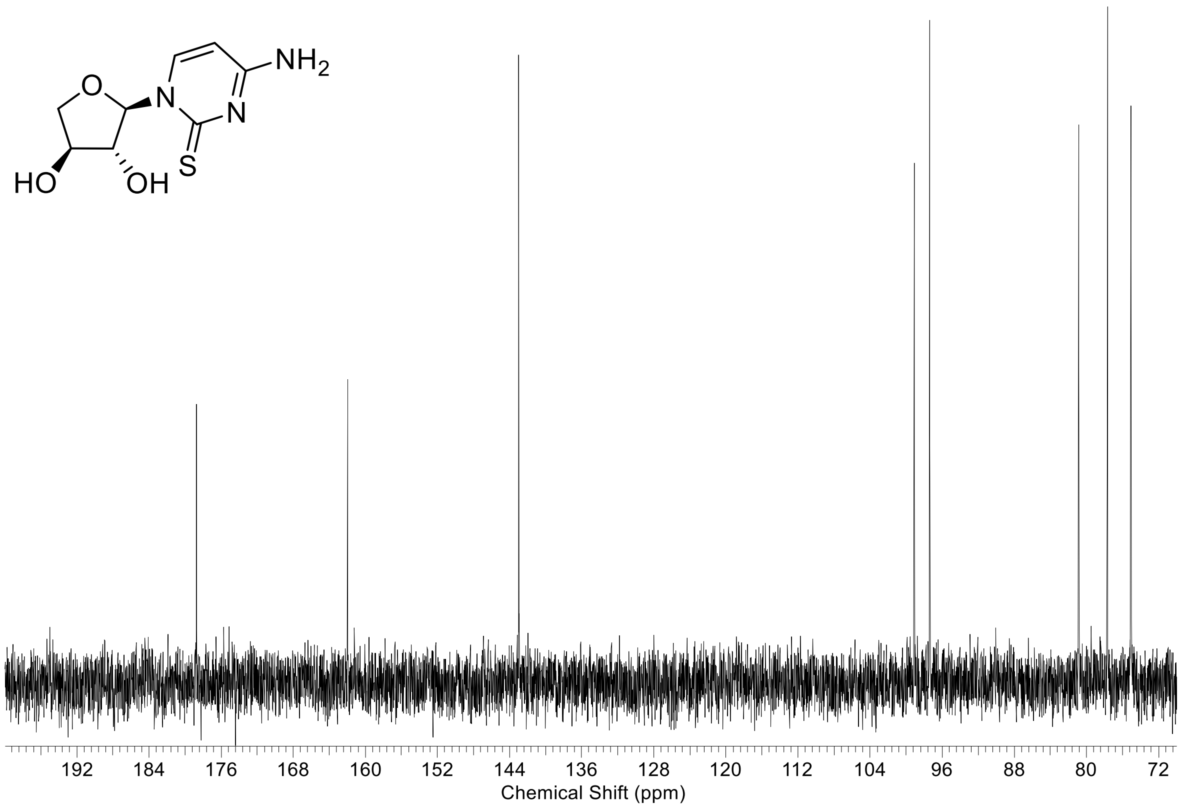

Supplementary Figure 39 *^{13}C NMR (100 MHz, D_2O, 70-200 ppm) spectrum to show α-threo-thiocytidine* ***8****.*

Synthetic route for authentic standard of α-*erythro*-**11**

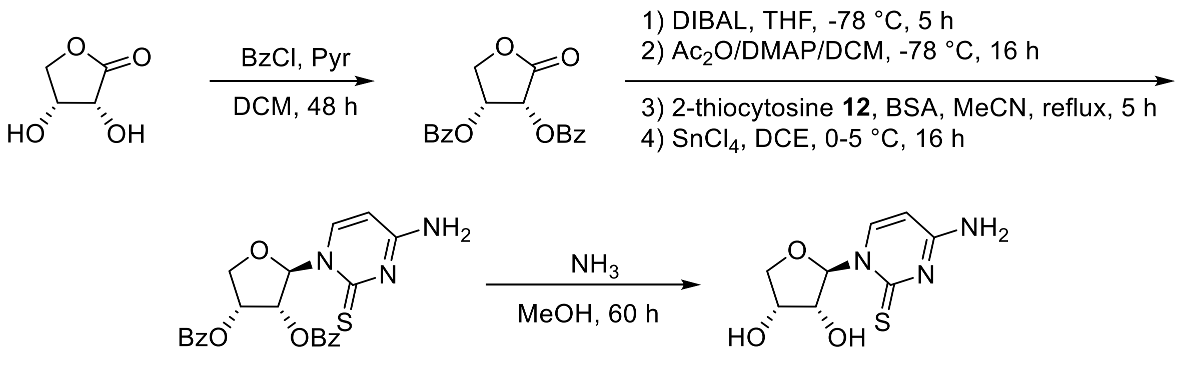

α-*erythro*-**11**

Synthesis of 2′,3′-di-*O*-benzoyl-erythronolactone

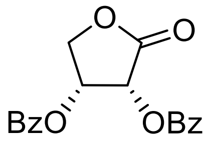

L-Erythronolactone (5 g, 42.3 mmol) and DMAP (100 mg, 0.82 mmol) were dissolved in anhydrous pyridine (80 mL) at 0 °C. Benzoyl chloride (12 mL, 103 mmol) was added dropwise under a N_2 atmosphere over 30 min. The mixture was allowed to come to room temperature and stirred for 2 days. The now heterogenous mixture was filtered and solvent removed in vacuo. The residue was co-evaporated with DCM (4 x 50 mL) and washed with 1 M HCl (3 x 50 mL), water (50 mL), $NaHCO_3$ sat. (50 mL), and brine (50 mL) before drying over $MgSO_4$, filtration and removing the solvent in vacuo to give 2,3-di-*O*-benzoyl-erythronolactone (12.2 g, 88.3 %) as an amorphous off-white solid.

^{1}H NMR (700 MHz, $CDCl_3$)	δ = 7.98 (app. d, *J* = 7.5 Hz, 4H, Ar-H), 7.59 (tt, *J* = 1.2, 7.4 Hz, 1H, (C3′)-Ar-H), 7.55 (tt, *J* =1.2, 7.4 Hz, 1H, (C2′)-Ar-H), 7.43 (app. t, *J* = 7.5 Hz, 2H, (C3′)-Ar-H), 7.37 (app. t, *J* = 7.5 Hz, 2H, (C2′)-Ar-H), 5.99 (dd, *J* = 3.4, 5.4 Hz, 1H, (C3′)–H), 5.95 (d, *J* = 5.4 Hz, 1H, (C2′)–H), 4.71 (dd, *J* = 3.4, 11.4 Hz, 1H, (C4′)–H), 4.66 (d, *J* = 11.4 Hz, 1H, (C4′′)–H)
^{13}C NMR (176 MHz, $CDCl_3$)	δ = 170.4 (C1), 165.4 (C3′O$\underline{C}$OAr), 165.1 (C2′O$\underline{C}$OAr), 134.0 (C2′/3′-Ar-*para*), 134.0 (C2′/3′-Ar-*para*), 130.2 (2C, C2′/3′-Ar-*ortho*), 130.0 (2C, C2′/3′-Ar-*ortho*), 128.7 (2C, C3′-Ar-*meta*), 128.7 (C3′-Ar-*ipso*), 128.6 (2C, C2′-Ar-*meta*), 128.2 (C2′-Ar-*ipso*), 70.1 (C4), 69.8 (C3), 67.9 (C2)
HRMS	ESI-HRMS (pos. m/z): $[M+H]^+$ calculated for $C_{18}H_{15}O_6^+$: 327.0863; found 327.0865
IR (solid, cm^{-1})	1794 (strong, C=O), 1723 (strong, C=O)
Melting Point (°C)	100-108 (110 - 111[6])

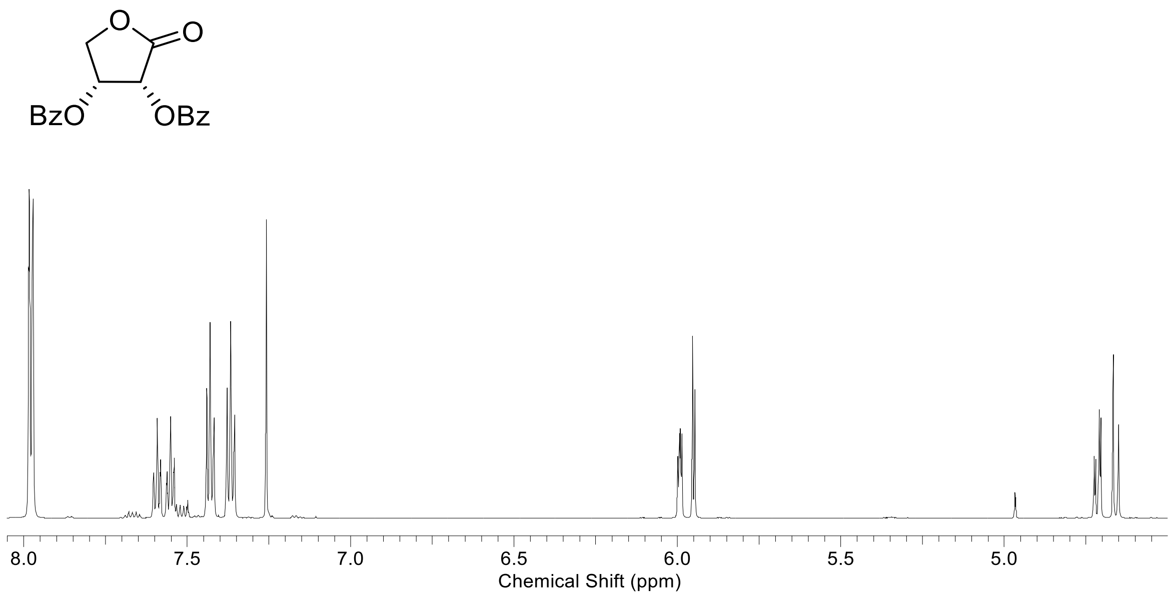

Supplementary Figure 40 *^{1}H NMR (700 MHz, $CDCl_3$, zg30, 4.0-8.5 ppm) spectrum to show 2',3'-di-O-benzoyl-erythronolactone.*

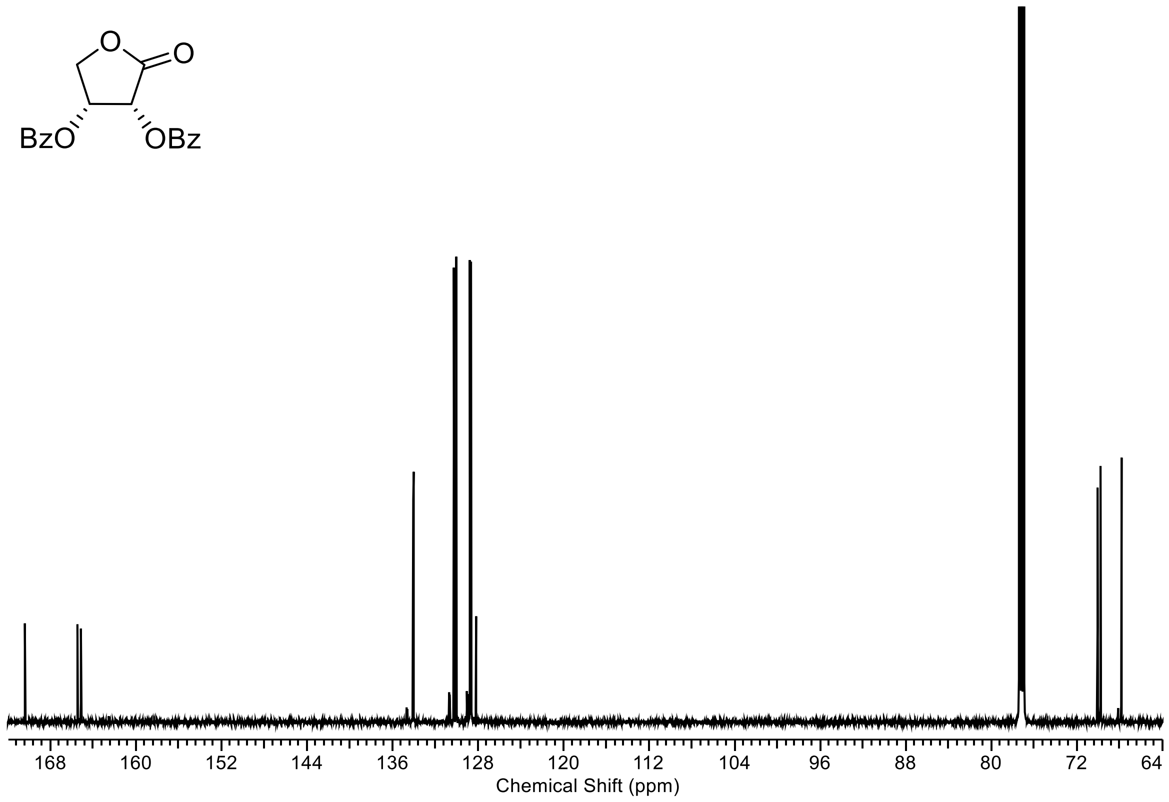

Supplementary Figure 41 *^{13}C NMR (176 MHz, $CDCl_3$, 64-172 ppm) spectrum to show 2’,3’-di-O-benzoyl-erythronolactone.*

Synthesis of 2′,3′-di-*O*-benzoyl-erythrothiocytidine

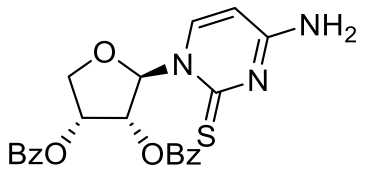

2′,3′-Di-*O*-benzoyl-erythronolactone (1.63 g, 5.0 mmol) was added under a N_2 atmosphere and dissolved in anhydrous THF (50 mL). The mixture was cooled to -78 °C and DIBAL-H (1.2 M in toluene, 11.5 mL, 13.8 mmol) added dropwise over 2 h. The reaction was maintained at -78 °C for 10 h and monitored by TLC. When the starting material was consumed Ac_2O/DCM/DMAP (4.7 ml, 49.7 mmol/3.5 ml/ 100 mg, 0.82 mmol) was added at -78 °C, over 20 mins, under a N_2 atmosphere. The reaction was allowed to warm to room temperature over 16 h. Hexane (100 mL) was added and the mixture was added to rapidly stirring 1 M HCl (100 mL) and stirred vigorously for 1 h. The aqueous layer was separated and washed with EtOAc (3 x 50 mL). The combined organic layers were separated and washed with water (50 mL), $NaHCO_3$ sat. (50 mL) and brine (50 mL), dried over $MgSO_4$ and evaporated to give a crude yellow oil (1.66 g, 90%). A portion (1 g, 2.7 mmol) of this was dissolved in DCM (15 mL) and co-evaporated 3 times before anhydrous dichloroethane (DCE) (15 mL) was added under N_2 atmosphere. To this mixture, freshly prepared silylated 2-thiocytosine **12** (700 mg, 5.5 mmol, dissolved in 15 mL anhydrous DCE) was added and the mixture cooled to 0 °C before $SnCl_4$ (2 mL, 17.1 mmol) was added over 30 minutes. The now orange homogenous mixture was allowed to come to room temperature for 16 h and monitored by TLC. When the reaction was judged complete the reaction was quenched with $NaHCO_3$ sat. (25 mL) and stirred vigorously for 2 h. The mixture was twice filtered through a Celite® 545, washed with DCM (50 mL) and $NaHCO_3$ sat. (25 mL) and the organics separated. The aqueous phase was washed with DCM (3 x 20 mL). The combined organics were washed with brine (50 mL) and dried over $MgSO_4$ before the solvent was removed in vacuo. The crude product was purified with FCC eluting with a mixture of DCM/MeOH to give 2′,3′-di-*O*-benzoyl-2-thiothreocytidine (547.9 mg, 56% (2 steps)) as a dark brown solid.

^{1}H NMR (700 MHz, $CDCl_3$)	*δ* = 8.08 (d, *J* = 1.3, 8.4 Hz, 2H, (C2′)-Ar-H-*ortho*), 7.90 (d, *J* = 1.3, 8.4 Hz, 2H, (C3′)-Ar-H-*ortho*), 7.66 (d, *J* = 7.5 Hz, 1H, (C6)–H), 7.57 (tt, *J* = 1.3, 7.4 Hz, 1H, (C2′)-Ar-H-*para*), 7.53 (tt, 1H, J = 1.3, 7.4 Hz, (C3′)-Ar-H-*para*), 7.50 (app tt, *J* = 1.7, 7.5 Hz, 2H, (C2′)-Ar-H-*meta*), 7.35 (app tt, *J* =1.76, 7.5 Hz, 2H, (C3′)-Ar-H-*meta*), 7.32 (d, *J* = 3.6 Hz 1H, (C1′)–H), 6.08 (d, 1H, *J* = 7.5 Hz, (C5)–H), 5.90 (dd, *J* = 3.6, 5.2, 1H, (C2′)–H), 5.64 (q, *J* = 6.1 Hz, 1H, (C3′)–H), 4.62 (ABX, *J* = 6.3, 9.9 Hz, 1H, (C4′)–H) 4.28 (ABX, *J* = 6.3, 9.9 Hz, 1H, (C4′′)–H)
^{13}C NMR (151 MHz, $CDCl_3$)	*δ* = 180.2 (C2), 165.7 (C′3-OC̲OAr), 165.4 (C′2-OC̲OAr), 160.0 (C4), 140.2 (C6), 133.7 (C3′-Ar-*para*), 133.7 (C2′-Ar-*para*), 130.2 (2C, C3′-Ar-*ortho*), 130.0 (2C, C2′-Ar-*ortho*), 129.1 (*ipso*), 128.9 (*ipso*), 128.6 (2C, C3′-Ar-*meta*), 128.6 (2C, C2′-Ar-*meta*), 99.6 (C5), 92.3 (C1′) 75.7 (C2′), 71.6 (C4′), 70.6 (C3′)
HRMS	ESI-HRMS (pos. m/z): $[M+H]^+$ calculated for $C_{22}H_{20}N_3O_5S^+$: 438.1124; found 438.1153.
IR (solid, cm^{-1})	1720 (strong, C=O), 1648 (strong, C=C), 1247 (medium, broad C-N)
Melting Point (°C)	128 (decomp.)

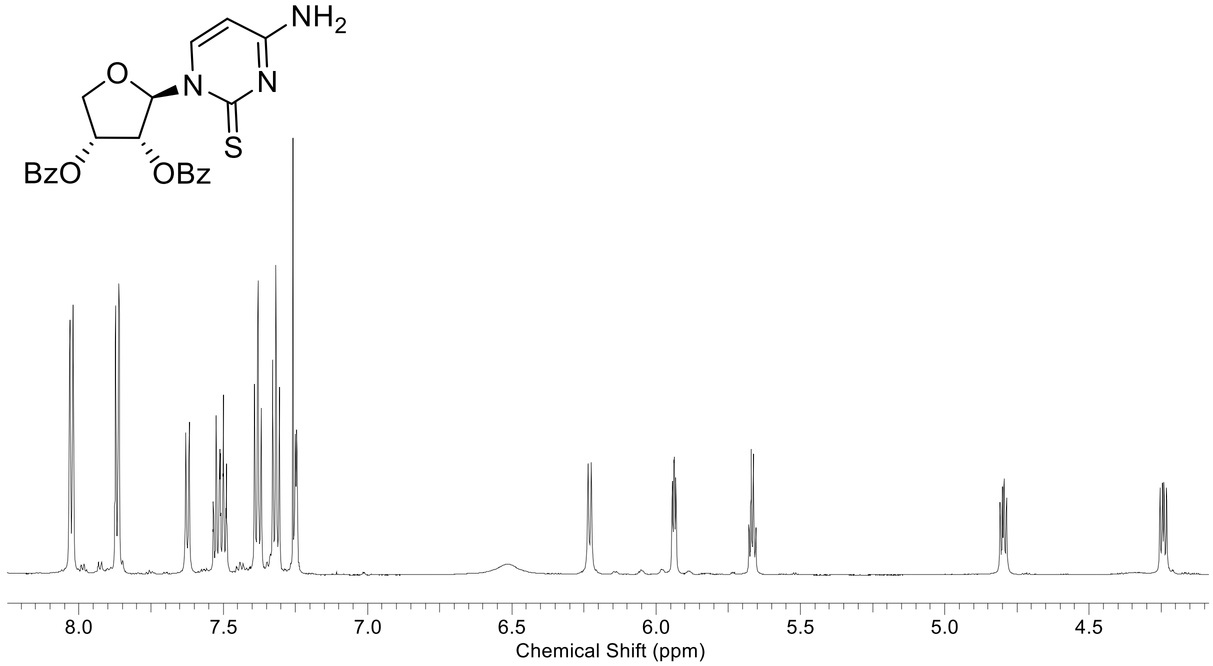

Supplementary Figure 42 *^{1}H NMR (700 MHz, $CDCl_3$, zg30, 4-8.25 ppm) spectra to show 2',3'-di-O-benzoyl-erythrothiocytidine.*

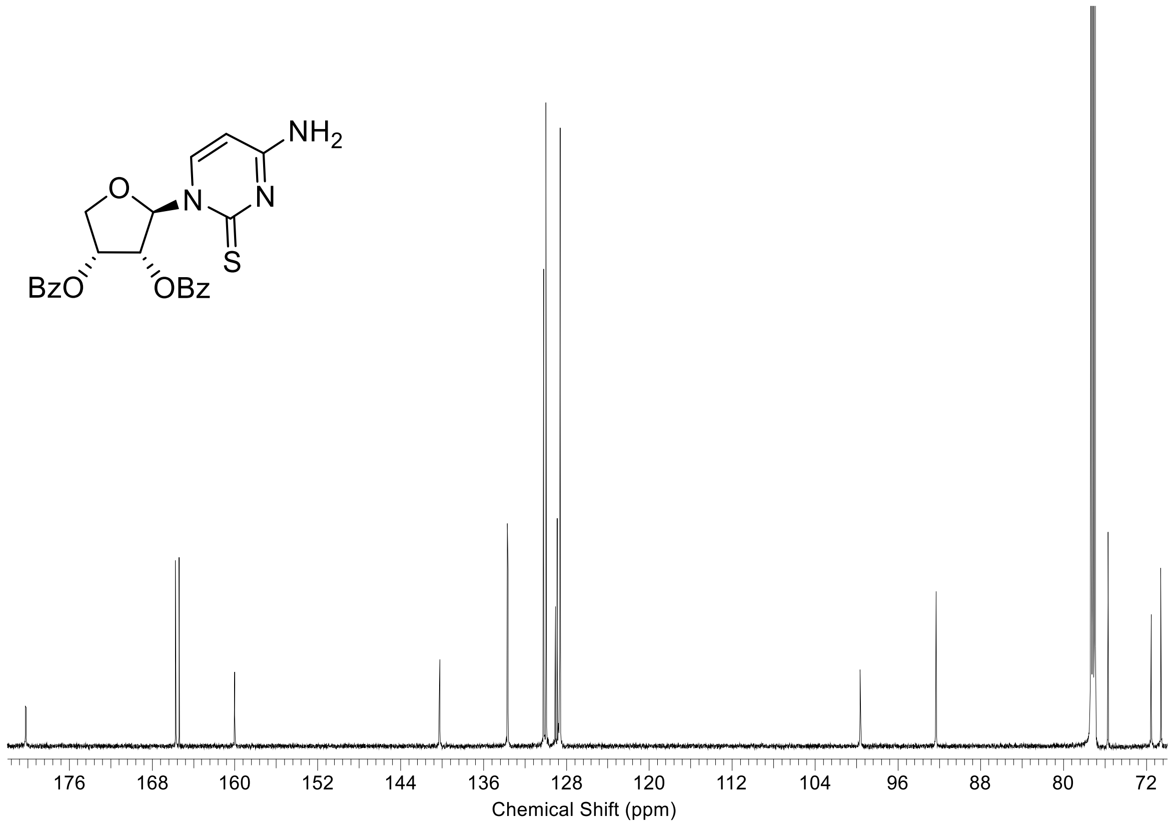

Supplementary Figure 43 *^{13}C NMR (176 MHz, $CDCl_3$, 70-182 ppm) spectrum to 2',3'-di-O-benzoyl-erythrothiocytidine.*

Synthesis of α-*erythro*-thiocytidine **11**

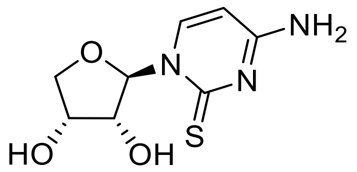

2,3-O-benzoyl-erythrothiocytidine (109.1 mg, 0.25 mmol) was dissolved in 7 M NH_3/MeOH (2 mL) and MeOH (8 mL) and the heterogenous mixture stirred at room temperature for 2.5 days. The solvent was evaporated and the residue redissolved in D_2O (7 mL) and washed DCM (3 x 7 mL). The aqueous layer was separated and lyophilised giving α-*erythro*-thiocytidine **11** (53.4 mg, 93 %) as a white powder. A sample was removed for NMR analysis.

^{1}H NMR (700 MHz, D_2O)	δ = 7.76 (d, J = 7.6 Hz, 1H, (C5)–H), 6.70 (d, J = 3.4, 1H, (C1′)–H), 6.33 (d, J = 7.6 Hz, 1H, (C6)–H), 4.44 (dd, J = 5.2, 9.8 Hz, 1H, (C4′)–H), 4.38 (m, 2H, (C2′)–H, (C3′)–H), 4.00 (dd, J = 4.7, 9.8 Hz, 1H, (C4′′)–H)
^{13}C NMR (176 MHz, D_2O)	δ = 179.7 (C2), 161.5 (C4), 141.7 (C6), 100.5 (C1′), 95.2 (C5), 76.4 (C2′), 73.4 (C4′), 70.0 (C3′)
HRMS	ESI-HRMS (pos. m/z): $[M+H]^+$ calculated for $C_8H_{12}N_3O_3S^+$: 230.0594; found 230.0597.
IR (solid, cm^{-1})	3201 (broad, weak O-H), 1640 (strong, sharp, C-N), 1045 (strong, sharp, C-N)
Melting Point (°C)	149 (decomp.)

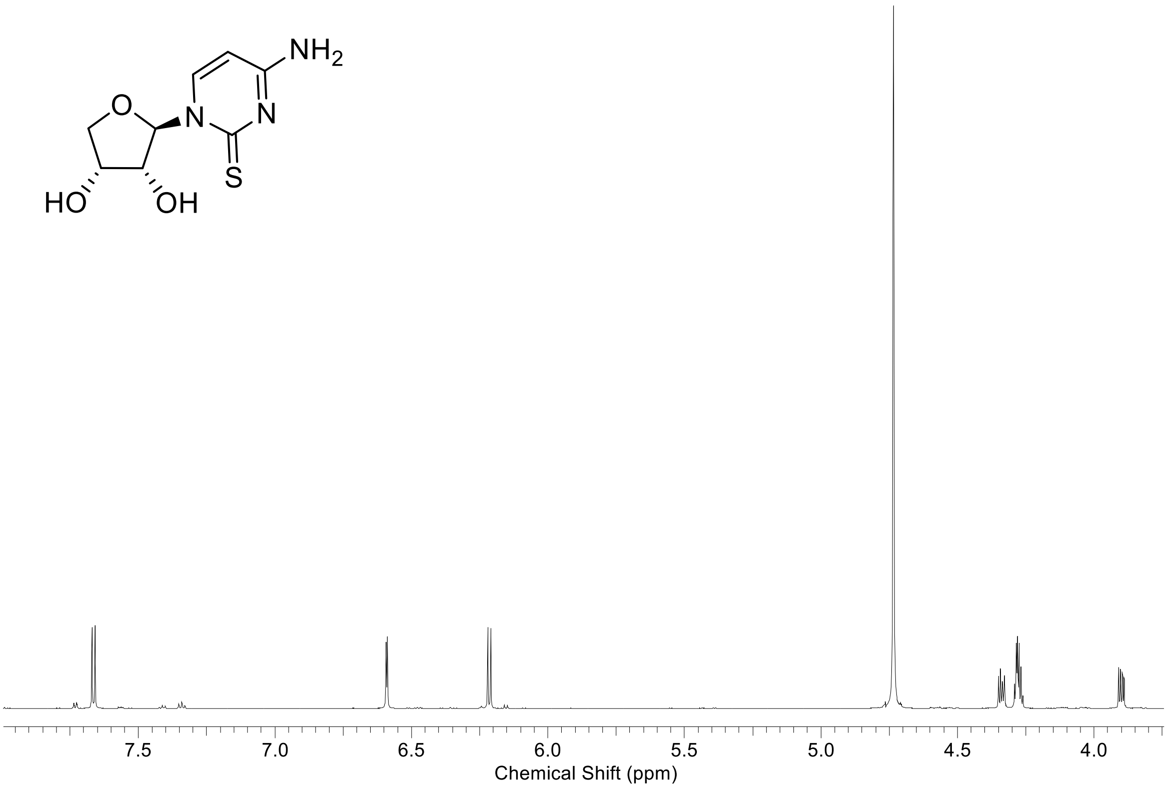

Supplementary Figure 44 *^{1}H NMR (700 MHz, D_2O, zg30, 3.75-8.0 ppm) spectrum to show α-erythrothiocytidine* ***11****.*

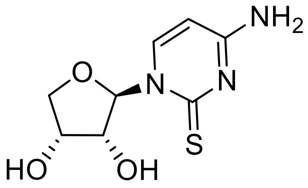

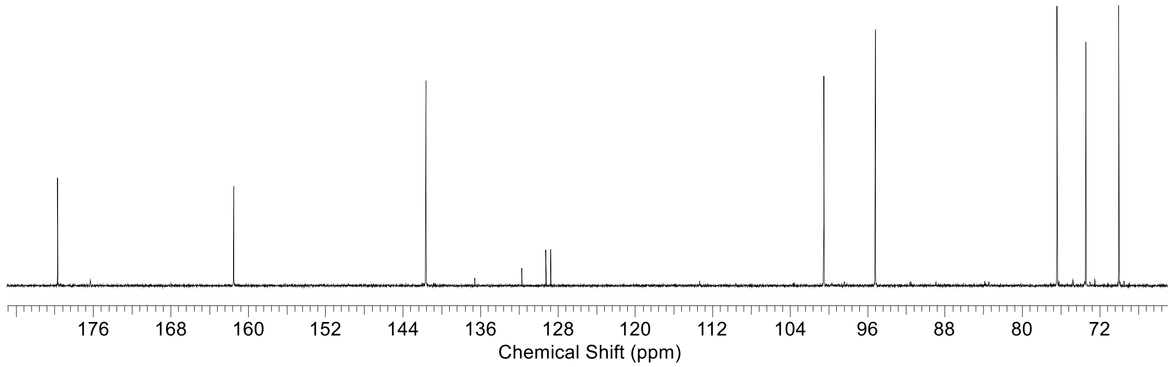

Supplementary Figure 45 *^{13}C NMR (176 MHz, D_2O, 65-185 ppm) spectrum to show α-erythrothiocytidine* ***11***.

Synthesis of β-*erythro*-thiocytidine-2′,3′-cyclic phosphate **13**

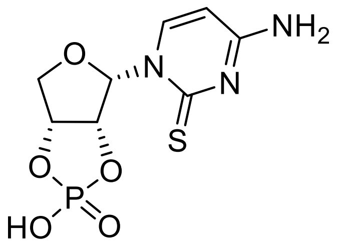

β-*Erythro*-thiocytidine (4.45 mg, 0.02 mmol), urea (96 mg, 1.6 mmol) and ammonium phosphate (12 mg, 0.1 mmol) were heated at 140 °C for 30 mins, allowed to cool and dissolved in H_2O (3 mL) and lyophilised. The resultant brown residue was dissolve in triethyl ammonium carbonate (TEAB, 1 mL 10 mM, pH 9) and purified by FCC (C18) eluting with TEAB/MeOH. Multiple lyophilisations gave β-*erythro*-thiocytidine 2′,3′-cyclic phosphate **13** as white powder (2.8 mg, 50 %).

^{1}H NMR (700 MHz, D_2O)	δ = 7.92 (d, J = 7.7 Hz, 1H, (C6)–H), 6.61 (app. dd, J = 1.9, 4.0 Hz, 1H, (C1′)–H), 6.32 (d, J = 7.7 Hz, 1H, (C5)–H), 5.37 (m, 1H, (C2′)–H), 5.12 (td, J = 4.1, 6.5 Hz, 1H, (C3′)–H), 4.36 (d, J = 11.6 Hz, 1H, (C4′)–H), 3.98 (dt, J = 11.6, 3.6 Hz, 1H, (C4′′)–H)
^{13}C NMR (176 MHz, D_2O)	δ = 178.8 (C2), 161.9 (C4), 144.0 (C5), 99.5 (C1′), 90.6 (C5), 78.2(C2′), 76.4(C3′), 72.0 (C4′)
^{31}P NMR (283 MHz, D_2O, ^{1}H-decoupled)	δ = 20.33 (s)
^{31}P NMR (283 MHz, D_2O)	δ = 20.33 (t, J = 7.3 Hz)
HRMS	ESI-HRMS (neg. m/z): $[M+H]^+$ calculated for $C_8H_{11}N_3O_5PS^+$: 292.0151; found 292.0150.

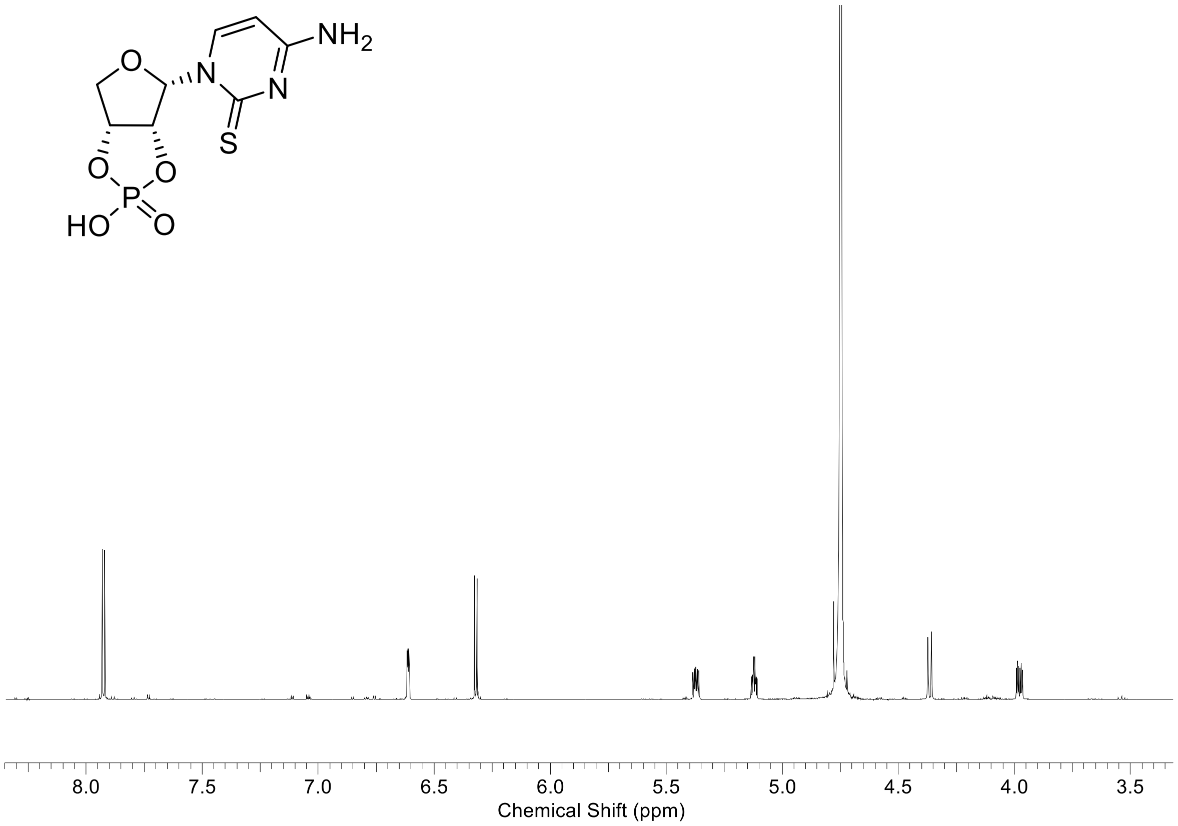

Supplementary Figure 46 *^{1}H NMR (700 MHz, D_2O, zg30, 3.3-8.3 ppm) spectrum to show β-*erythro-*thiocytidine 2',3'-cyclic phosphate* ***13****.*

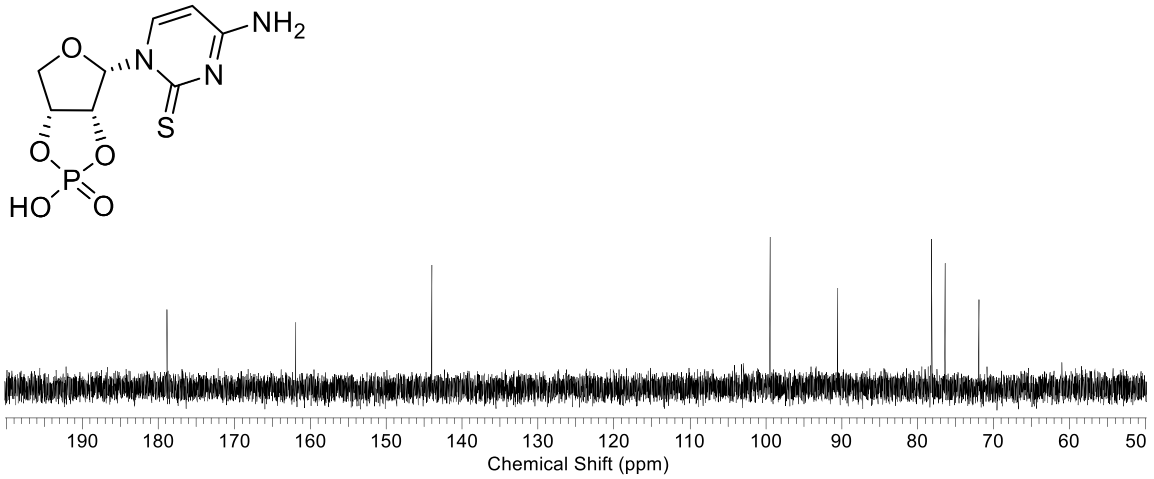

Supplementary Figure 47 *^{13}C NMR (176 MHz, D_2O, 50-200 ppm) spectrum to show β-*erythro-*thiocytidine 2',3'-cyclic phosphate* ***13****.*

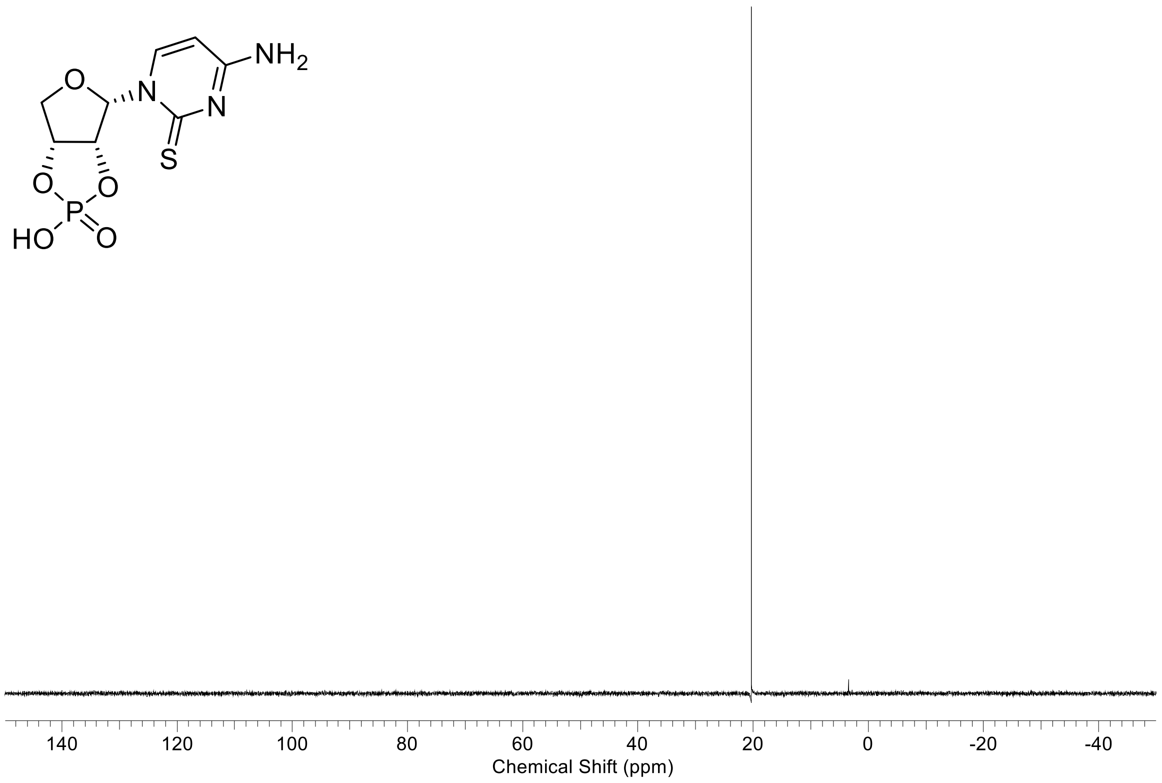

Supplementary Figure 48 *^{31}P NMR (283 MHz, D_2O, ^{1}H-decoupled, -50-150 ppm) spectrum to show β-*erythro*-thiocytidine2′,3′-cyclic phosphate* ***13****.*

References

[1] S. Stairs, A. Nikmal, D.-K. Bučar, S. L. Zheng, J. W. Szostak, M. W. Powner, D. K. Bŭcar, *Nat. Commun.* **2017**, *8*, 15270.

[2] S. P. Sau, N. E. Fahmi, J. Y. Liao, S. Bala, J. C. Chaput, *J. Org. Chem.* **2016**, *81*, 2302–2307.

[3] J. Zeng, Q. Zhang, H.-K. Zhang, A. Chen, *RSC Adv.* **2013**, *3*, 20298.

[4] K.-U. Schöning, P. Scholz, X. Wu, S. Guntha, G. Delgado, R. Krishnamurthy, A. Eschenmoser, *Helv. Chim. Acta* **2002**, *85*, 4111–4153.

[5] F. Micheel, W. Peschke, *Chem. Ber.* **1942**, *75B*, 1603–1607.

[6] V. C. Jelinek, F. W. Upson, *J. Am. Chem. Soc.* **1938**, *60*, 355–357.
